# Supplementary material for: Novel mechanisms of MITF regulation identified in a mouse suppressor screen
Source: EMBO Rep. 2024 Aug 21;25(10):4252–80. doi: 10.1038/s44319-024-00225-3 (PMC11467436; doi:10.1038/s44319-024-00225-3)
Supplement: Supplementary file 1 — Appendix [file 44319_2024_225_MOESM1_ESM.pdf]

Table of Contents

Appendix Figure S1: MITF-sl can dimerize with non-DNA-binding mutant MITF proteins. 2

Appendix Figure S2: Exon 6A and the tags did not affect MITF stability. 3

Appendix Figure S3: Exon 6A and the tags did not affect MITF nuclear localization. . . 4

Appendix Figure S4: MITF-sl enables nuclear localization of its dimer partner 5

Appendix Figure S5: The four Individual phosphorylation sites at C-terminus (S384, S397, S401, and S405) did not affect MITF localization 7

Appendix Figure S6: The carboxyl-domains of Mitf also affect MITF-mi and MITF-ew nuclear localization 9

Appendix Figure S7: The four Individual phosphorylation sites at C-terminus (S384, S397, S401, and S405) did not affect MITF stability 10

Appendix Figure S8: The carboxyl-domains of Mitf do not affect MITF-mi and MITF-ew nuclear stability 11

Appendix Figure S9: MITF is mainly degraded through the proteasome pathway in the nucleus 12

Appendix Figure S10: Mutation at four individual phosphorylation sites at C-end (S384, S397, S401, and S405) in combination with K316R and E318K do not affect MITF localization. 13

Appendix Figure S1: MITF-sl can dimerize with non-DNA-binding mutant MITF proteins.

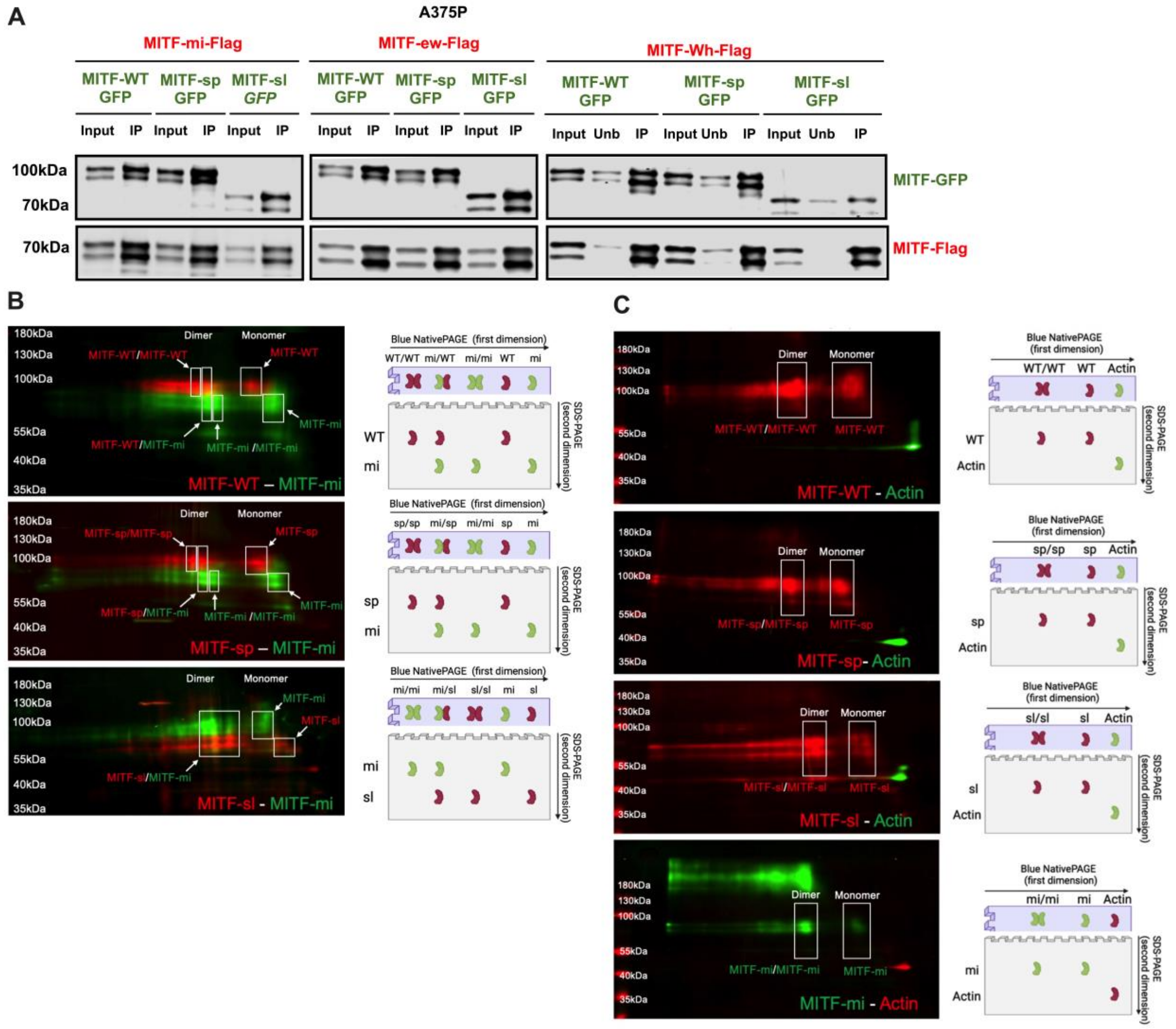

(A) Western blot analysis showing the results of a co-immunoprecipitation experiment. MITF-Wh-Flag, MITF-mi-Flag, or MITF-ew-Flag construct was cotransfected with either MITF-WT-GFP, MITF-sp-GFP, or MITF-sl-GFP construct in A375P melanoma cells. Co-immunoprecipitation (co-IP) of the whole cell lysate using FLAG-antibodies was performed, and proteins were visualized using FLAG and GFP antibodies. Input fraction (Input), Unbound fraction (Unb), and Immunoprecipitated fraction (IP) are indicated on the western blot.

(B) and **(C)** Wild type and mutant MITF proteins visualized after a Blue native PAGE followed by the second dimension of SDS-PAGE. A375P melanoma cells transiently (B) co-expressing the MITF-mi-Flag protein (green) together with either MITF WT-GFP, MITF-sp-GFP, or MITF-sl-GFP proteins (red) or (C) expressing MITF-WT-Flag, MITF-sp-Flag, MITF-sl-Flag (red) or MITF-mi-Flag (green) were generated. We then performed Blue native PAGE electrophoresis followed by a second dimension of SDS-PAGE. Actin was visualized by using mouse or rabbit Actin antibodies (C) and then anti-mouse (red) or anti-rabbit (green) secondary antibodies. The cartoon (on the left) illustrates the migration patterns of dimeric and monomeric proteins after undergoing Blue Native PAGE and SDS-PAGE, demonstrating their relative mobility and separation on the gel.

Appendix Figure S2: Exon 6A and the tags did not affect MITF stability.

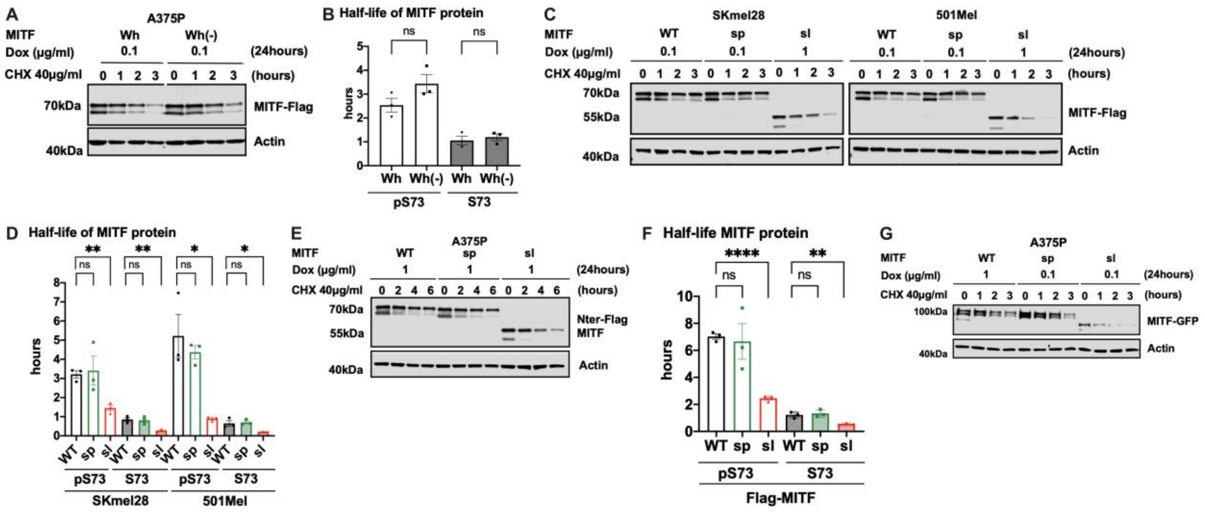

(A), (C), (E), and **(G)** Western blot analysis of wild type and mutant MITF proteins with different tags at either N-terminus or C-terminus. The dox-inducible A375P cells were treated with doxycycline for 24h to express the indicated MITF proteins before treating them with 40 µg/ml CHX for 0, 1, 2, and 3 hours. The MITF proteins were then compared by western blot using the FLAG antibody. Actin was used as a loading control. The band intensities were quantified using ImageJ software.

(B), (D), and **(F)** Half-life analysis of the pS73- and S73-MITF proteins over time after CHX treatment. The MITF protein levels relative to T0 were calculated, and non-linear regression analysis was performed. Error bars represent SEM of at least three independent experiments. Statistically significant differences (Student's t-test) are indicated by *p< 0.05, ** p < 0.01, **** p < 0.0001, and ns not significant.

Appendix Figure S3: Exon 6A and the tags did not affect MITF nuclear localization.

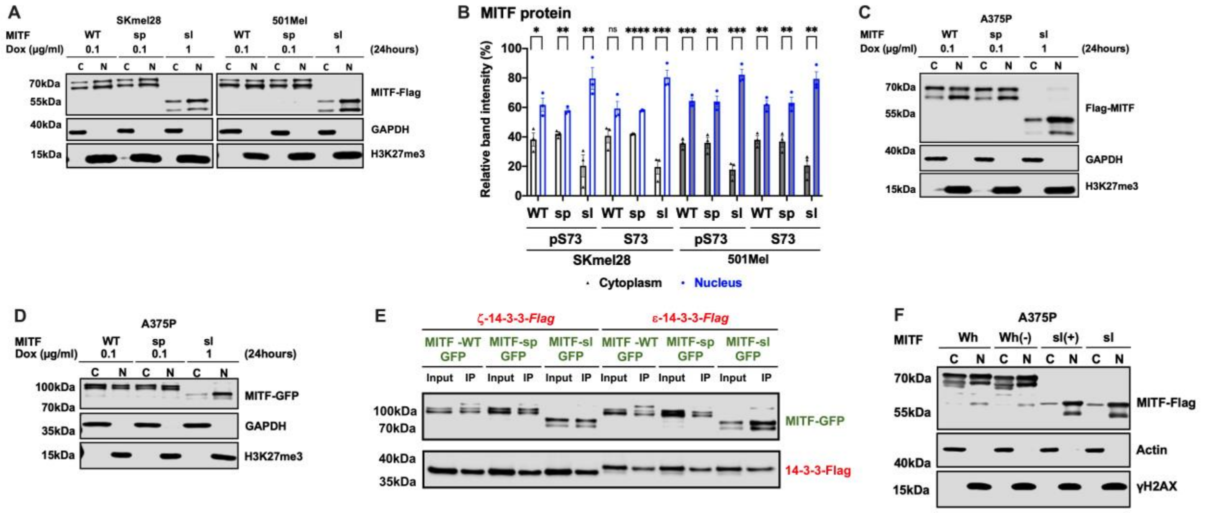

(A), (C), (D), and **(F)** Western blot analysis of cytoplasmic (C) and nuclear (N) fractions from either 501Mel (A), SKMel28 (A) or A375P (C, D, and F) melanoma cells induced for 24 hours to overexpress the MITF mutant proteins with either Flag-tag at C-terminus (A and F) or Flag-tag at N-terminus (C) or GFP-tag at C-terminus (D) visualized using either FLAG or GFP antibody. GAPDH and H3K27me3 were loading controls for cytoplasmic and nuclear fractions, respectively.

(B) The intensities of the pS73- and S73-MITF proteins from the Western blots in (A) were quantified separately with *ImageJ* software and are depicted as percentages of the total amount of protein present in the two fractions. Error bars represent SEM of three independent experiments. Statistically significant differences (Student's t-test) are indicated by *p< 0.05, ** p < 0.01, *** p < 0.001, **** p < 0.0001, and ns not significant.

(E) Western blot analysis showing the results of a co-immunoprecipitation experiment. MITF^{mi-sp}-GFP, MITF^{mi-sl}-GFP, or MITF-WT-GFP constructs were cotransfected with either e-14-3-3-Flag or z-14-3-3-Flag in A375P melanoma cells. Co-immunoprecipitation (co-IP) of whole cell lysates using FLAG-antibodies was performed, and proteins were visualized using FLAG and GFP antibodies. Input fraction (Input), unbound fraction (Unb), and an immunoprecipitated fraction (IP) are indicated on the Western blot.

Appendix Figure S4: MITF-sl enables nuclear localization of its dimer partner

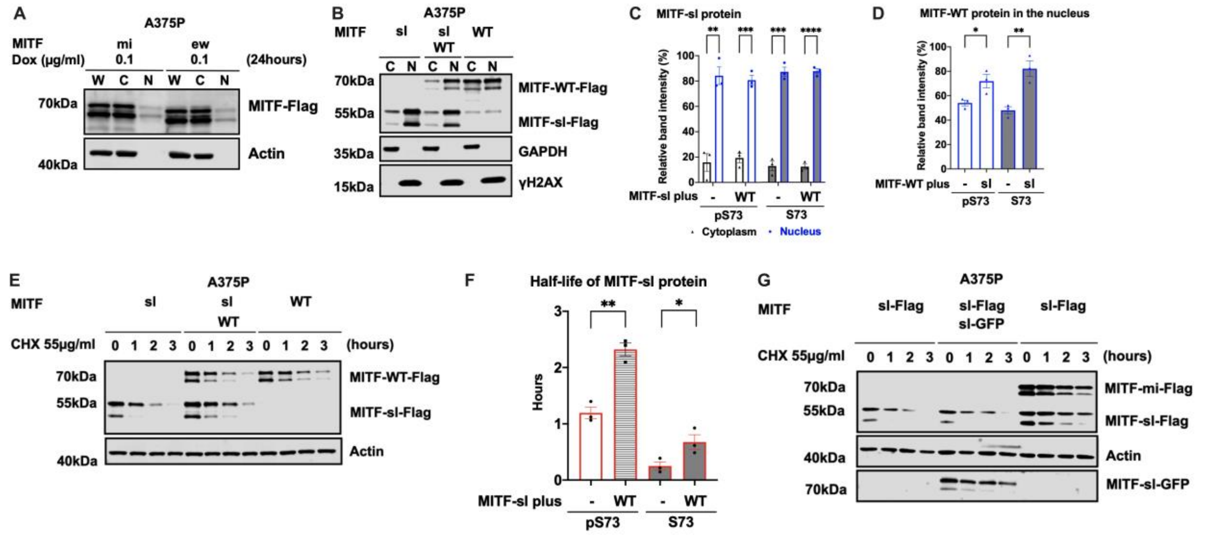

(A) Western blot analysis of subcellular fractions isolated from A375P melanoma cells induced for 24 hours to overexpress different MITF mutant proteins. MITF-mi and MITF-ew proteins in whole cell lysate (W), cytoplasmic (C) and nuclear (N) fractions were visualized using FLAG antibody. Actin was used as a loading control for the cytoplasm.

(B) Western blot analysis of subcellular fractions isolated from A375P cells transiently co-overexpressing the MITF-sl protein with MITF-WT. MITF proteins in cytoplasmic (C) and nuclear (N) fractions were visualized using FLAG antibody. GAPDH and gH2AX were loading controls for cytoplasmic and nuclear fractions, respectively.

(C) Intensities of the pS73- and S73-MITF-sl proteins in the cytoplasmic and nuclear fractions of A375P cells transiently co-overexpressing the MITF-sl protein together with MITF-WT were quantified separately with ImageJ software and are depicted as percentages of the total amount of protein present in the two fractions. Error bars represent SEM of three independent experiments. Statistically significant differences (Student's t-test) are indicated by ** $p < 0.01$, *** $p < 0.001$, and **** $p < 0.0001$.

(D) Intensities of the pS73- and S73- MITF-WT proteins in the nuclear fraction of A375P cells transiently co-overexpressing the MITF-sl protein together with MITF-WT proteins were quantified with ImageJ software and are depicted as percentages of the total amount of protein present in the two fractions. Error bars represent SEM of three independent experiments. Statistically significant differences (Student's t-test) are indicated by *$p< 0.05$ and ** $p < 0.01$.

(E) Western blot analysis of the MITF-sl protein in the presence of MITF-WT. The A375P cells were transiently co-transfected with MITF-sl and MITF-WT for 24 hours before being treated with 55 µg/ml CHX. The amount of MITF protein was then compared by western blot using FLAG antibody. Actin was used as a loading control. The band intensities were quantified using ImageJ software.

(F) Half-life analysis of the pS73- and S73-MITF proteins over time after CHX treatment. The MITF protein levels relative to T0 were calculated, and non-linear regression analysis was performed. Error bars represent SEM of at least three independent experiments. Statistically significant differences (Student's t-test) are indicated by *$p< 0.05$ and ** $p < 0.01$.

(G) Western blot analysis of the MITF-sl protein with Flag-tag at C terminus in the presence of either MITF-sl-GFP tagged at C-end or MITF-mi-Flag at C-end. The A375P cells were transiently co-transfected with MITF-sl-Flag and the MITF mutant proteins for 24 hours before being treated with 55 μg/ml CHX. The MITF protein was then compared by western blot using FLAG antibody. Actin was used as a loading control. The band intensities were quantified using ImageJ software.

Appendix Figure S5: The four Individual phosphorylation sites at C-terminus (S384, S397, S401, and S405) did not affect MITF localization

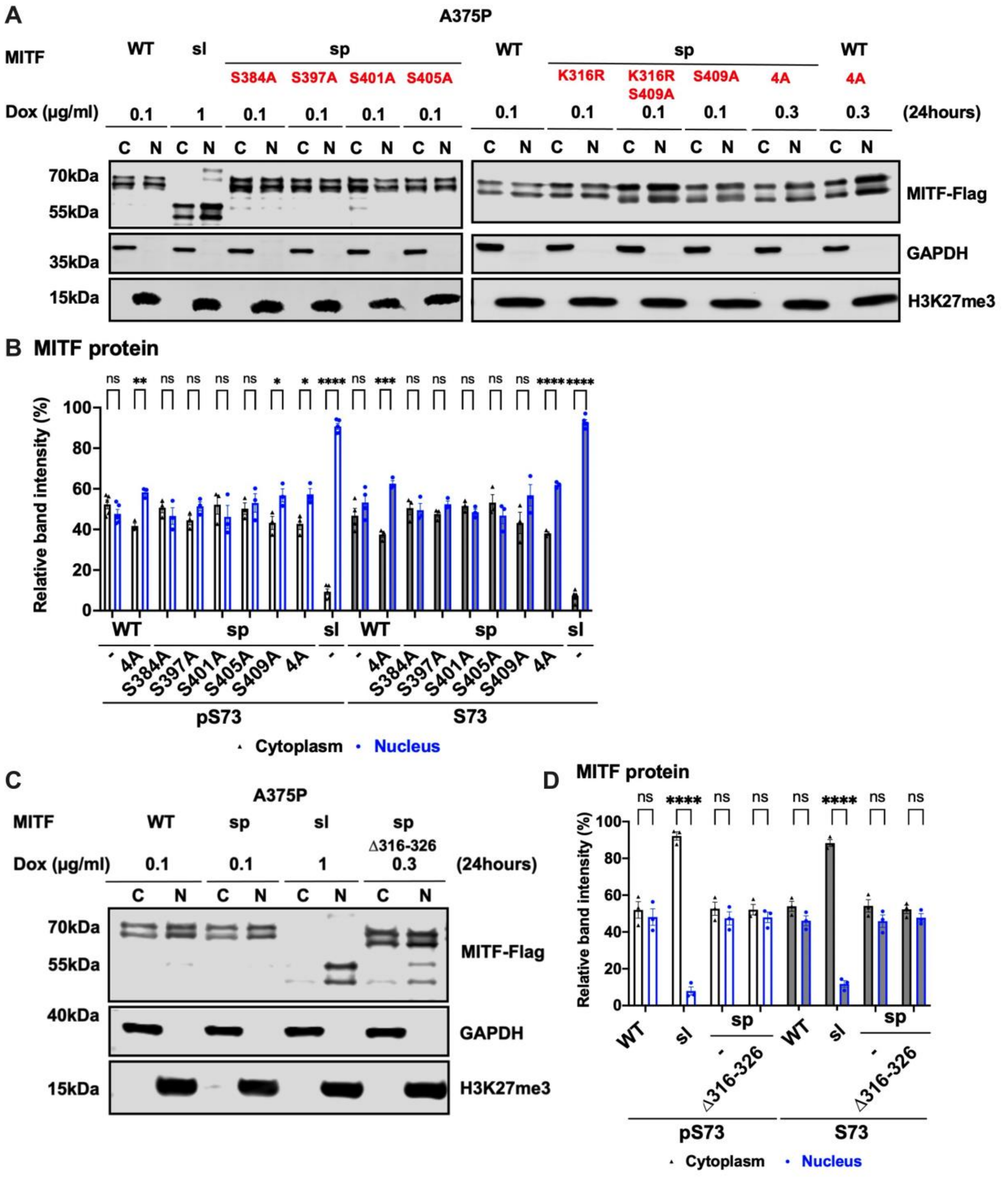

(A) and **(C)** Western blot analysis of cytoplasmic (C) and nuclear (N) fractions from A375P melanoma cells induced for 24 hours to overexpress the indicated MITF mutant proteins with or without treatment with 200nM TPA for 1 hour. MITF was visualized using FLAG antibody. GAPDH and H3K27me3 were loading controls for cytoplasmic and nuclear fractions, respectively.

(B) and **(D)** MITF band intensities in the cytoplasmic and nuclear fractions from western blot analysis (A, C, E, H, and J, respectively) were quantified separately with *ImageJ* software and are depicted as

percentages of the total amount of protein present in the two fractions. Error bars represent SEM of three independent experiments. Statistically significant differences (Student's t-test) are indicated by *p< 0.05, *** p < 0.001, **** p < 0.0001, and ns not significant.

Appendix Figure S6: The carboxyl-domains of Mitf also affect MITF-mi and MITF-ew nuclear localization

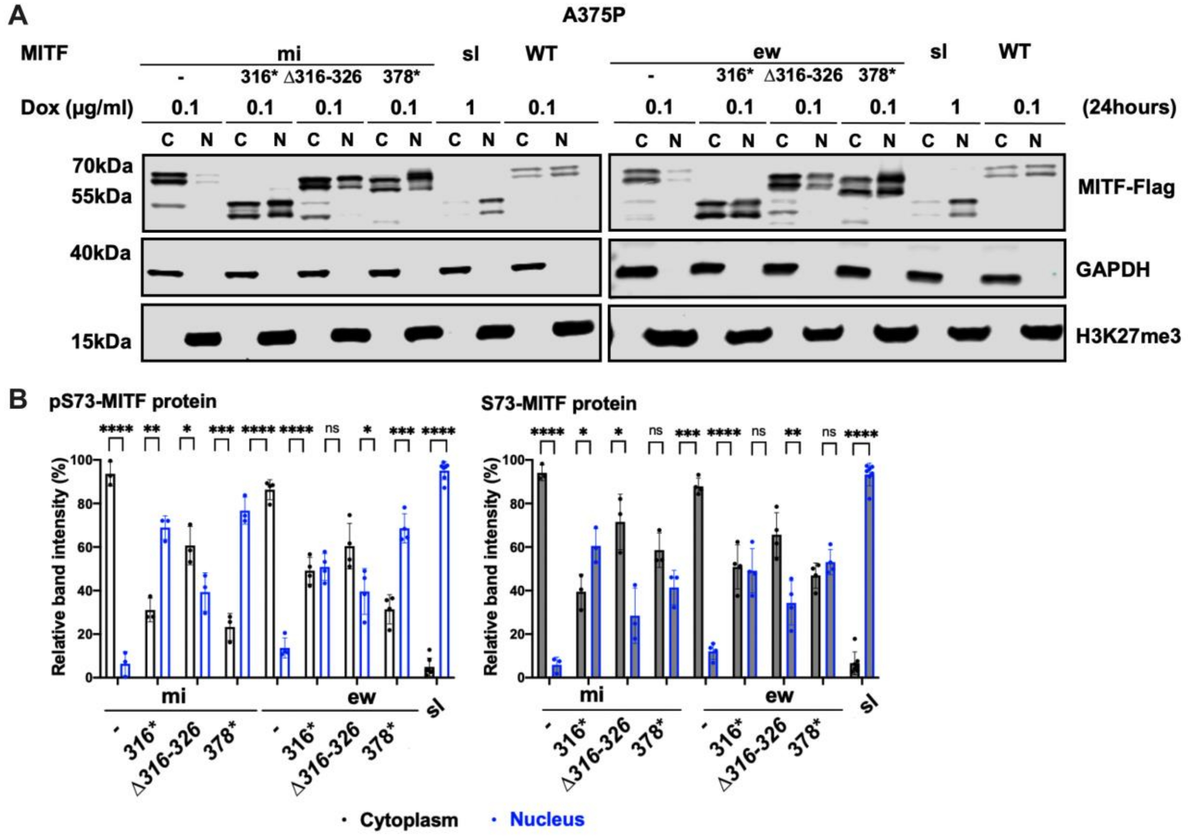

(A) Western blot analysis of cytoplasmic (C) and nuclear (N) fractions from A375P melanoma cells induced for 24 hours to overexpress the indicated MITF mutant proteins with or without treatment with 200nM TPA for 1 hour. MITF was visualized using FLAG antibody. GAPDH and H3K27me3 were loading controls for cytoplasmic and nuclear fractions, respectively.

(B) MITF band intensities in the cytoplasmic and nuclear fractions from western blot analysis (A, C, E, H, and J, respectively) were quantified separately with *ImageJ* software and are depicted as percentages of the total amount of protein present in the two fractions. Error bars represent SEM of three independent experiments. Statistically significant differences (Student's t-test) are indicated by *p< 0.05, ** p < 0.01, *** p < 0.001, **** p < 0.0001, and ns not significant.

Appendix Figure S7: The four Individual phosphorylation sites at C-terminus (S384, S397, S401, and S405) did not affect MITF stability

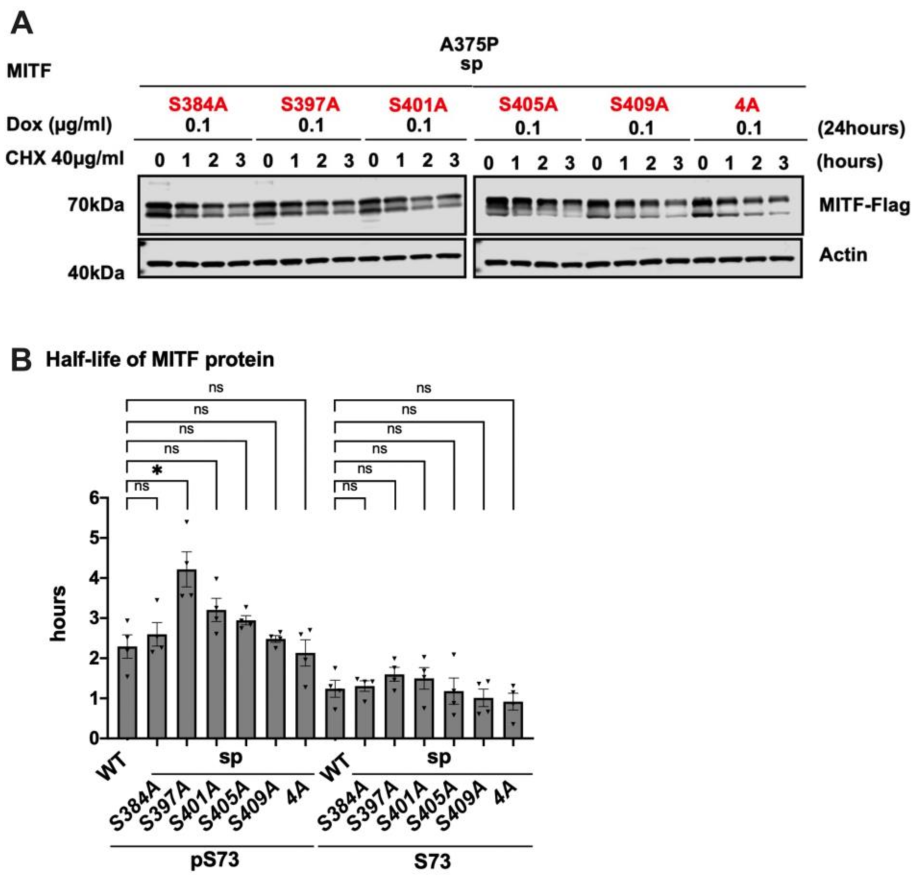

(A) The dox-inducible A375P cells were treated with doxycycline for 24h to express the indicated MITF proteins before treating them with 40 µg/ml CHX for 0, 1, 2, and 3 hours. The amount of MITF proteins were then visualized by western blot using FLAG antibody. Actin was used as a loading control. The band intensities were quantified using ImageJ software.

(B) Half-life analysis of the pS73- and S73-MITF proteins over time after CHX treatment. The MITF protein levels relative to T0 were calculated, and non-linear regression analysis was performed. Error bars represent SEM of at least three independent experiments. Statistically significant differences (Student's t-test) are indicated by *p< 0.05 and ns not significant.

Appendix Figure S8: The carboxyl-domains of Mitf do not affect MITF-mi and MITF-ew nuclear stability

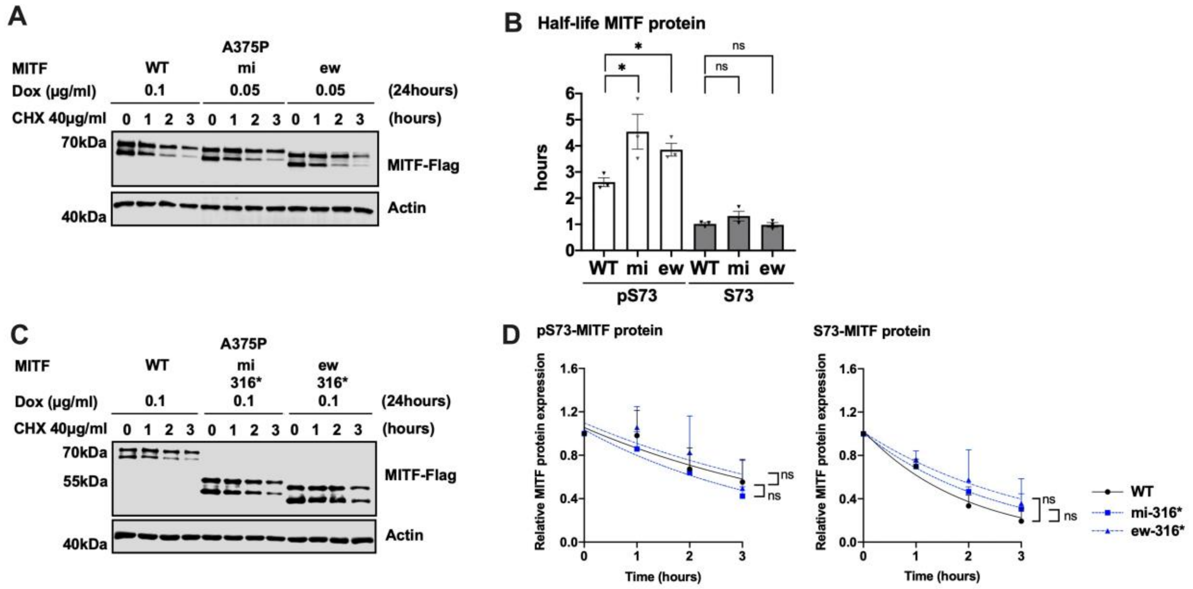

(A) and **(C)** The dox-inducible A375P cells were treated with doxycycline for 24h to express the indicated MITF proteins before treating them with 40 µg/ml CHX for 0, 1, 2, and 3 hours. The amount of MITF proteins were then visualized by western blot using FLAG antibody. Actin was used as a loading control. The band intensities were quantified using ImageJ software.

(B) Half-life analysis of the pS73- and S73-MITF proteins over time after CHX treatment. The MITF protein levels relative to T0 were calculated, and non-linear regression analysis was performed. Error bars represent SEM of at least three independent experiments. Statistically significant differences (Student's t-test) are indicated by *p< 0.05 and ns not significant.

(D) Non-linear regression (one-phase decay) analysis of the indicated pS73- and S73-MITF proteins over time after CHX treatment in A375P melanoma cells. The relative MITF protein levels to T0 were calculated, and non-linear regression analysis was performed. Error bars represent SEM of at least three independent experiments. Statistically significant differences (Student's t-test) are indicated by ns not significant.

Appendix Figure S9: MITF is mainly degraded through the proteasome pathway in the nucleus

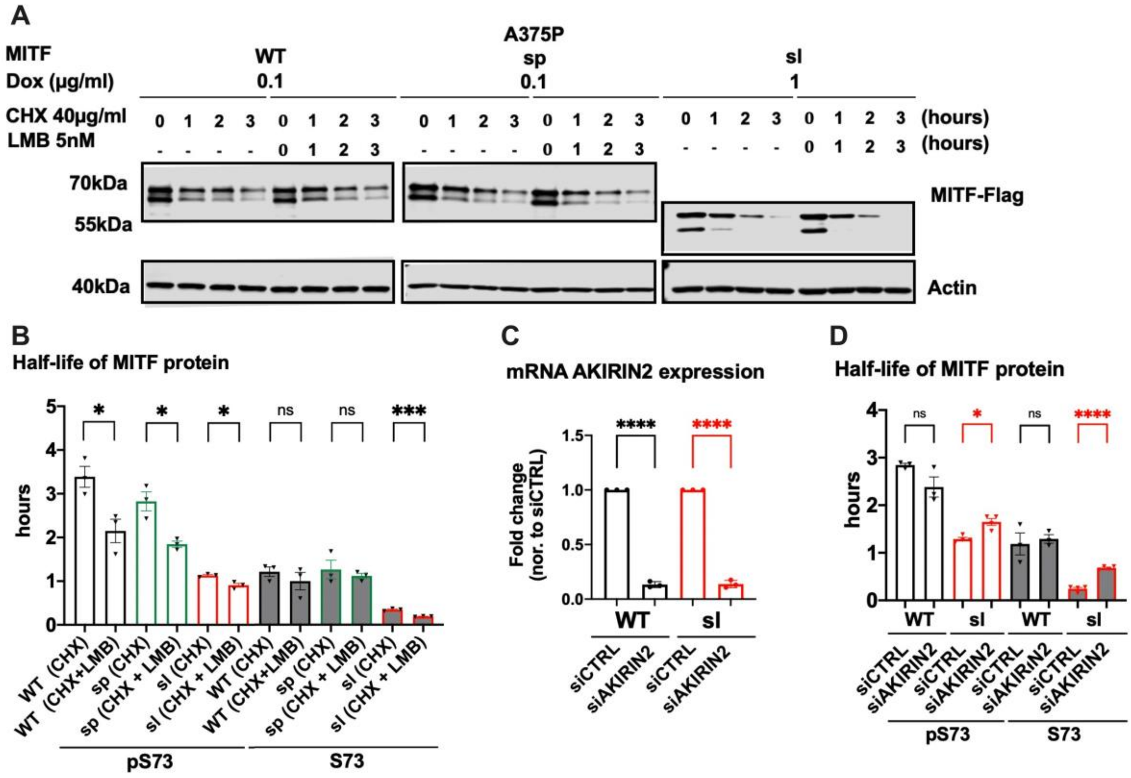

(A) Western blot analysis of the indicated MITF mutant proteins. The A375P cells were induced for 24 hours and treated with 40 µg/ml CHX in the presence of 5nM LMB for 0, 1, 2, and 3 hours. The MITF protein was visualized using FLAG antibody. Actin was used as a loading control. The band intensities were quantified using ImageJ software.

(B) Half-life analysis of the pS73- and S73-MITF proteins over time after CHX plus LMB treatment. The MITF protein levels relative to T0 were calculated, and non-linear regression analysis was performed. Error bars represent SEM of at least three independent experiments. Statistically significant differences (Student's t-test) are indicated by * p< 0.05, *** p < 0.001, and ns not significant.

(C) RT-qPCR analysis of AKIRIN2 gene expression in A375P cells treated with siAKIRIN2 for 24 hours and then induced by dox to induce MITF overexpression for 6 hours. The expression was normalized to siCTRL-treated cells. Error bars represent SEM of at least three independent experiments. Statistically significant differences (Student's t-test) are indicated by **** p < 0.0001.

(D) Half-life analysis of the pS73- and S73-MITF proteins in siAKIRIN2-treated A375P cells which are induced to overexpress MITF. The MITF protein levels relative to T0 were calculated, and non-linear regression analysis was performed. Error bars represent SEM of at least three independent experiments. Statistically significant differences (Student's t-test) are indicated by * p< 0.05, **** p < 0.00, and ns not significant.

Appendix Figure S10: Mutation at four individual phosphorylation sites at C-end (S384, S397, S401, and S405) in combination with K316R and E318K do not affect MITF localization.

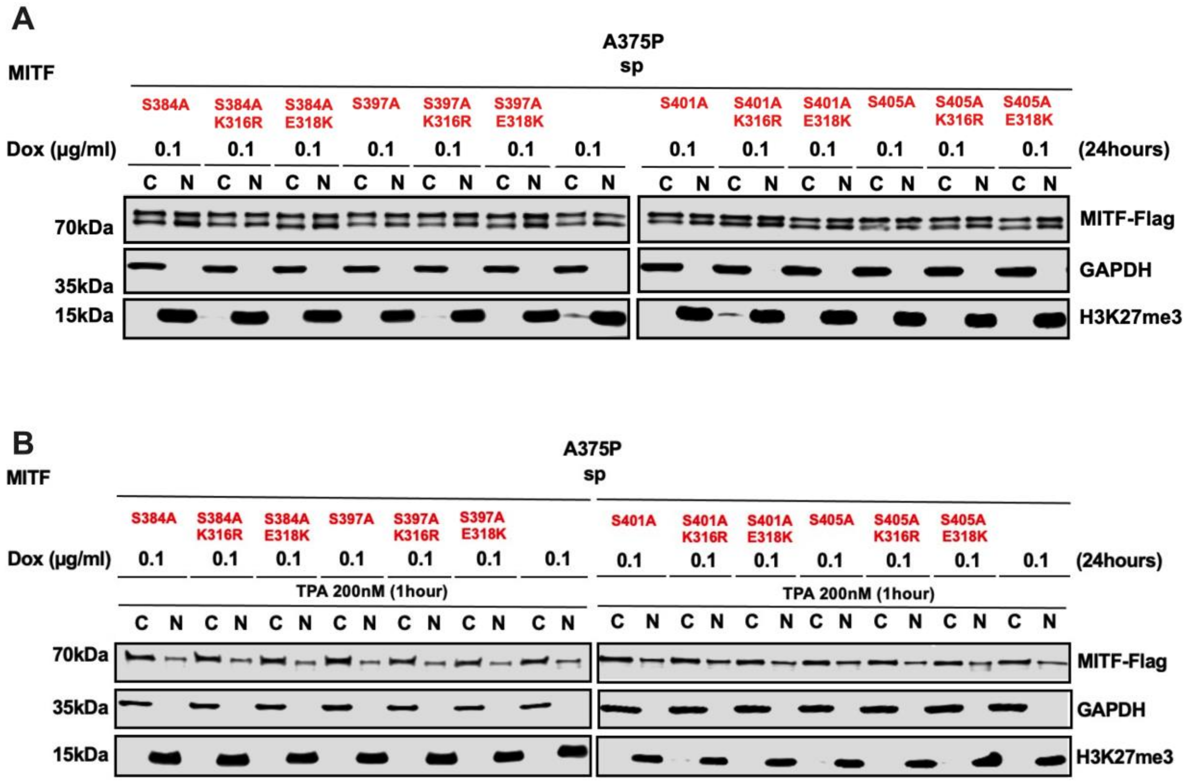

(A) Western blot analysis of subcellular fractions isolated from A375P melanoma cells induced to overexpress the indicated MITF mutant proteins. MITF protein in cytoplasmic (C) and nuclear (N) fractions were visualized using FLAG antibody. GAPDH and H3K27me3 were loading controls for cytoplasmic and nuclear fractions, respectively.

(B) Western blot analysis of subcellular fractions isolated from A375P melanoma cells induced to overexpress the indicated MITF mutant proteins before treatment with TPA at 200nM for 1 hour. MITF protein in cytoplasmic (C) and nuclear (N) fractions were visualized using FLAG antibody. GAPDH and H3K27me3 were loading controls for cytoplasmic and nuclear fractions, respectively.
