# Supplementary figures and images for: Novel mechanisms of MITF regulation identified in a mouse suppressor screen
Source: EMBO Rep. 2024 Aug 21;25(10):4252–80. doi: 10.1038/s44319-024-00225-3 (PMC11467436; doi:10.1038/s44319-024-00225-3)

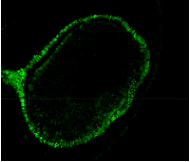

Supplement: Supplementary file 4 — Source data Fig. 1 [file 44319_2024_225_MOESM4_ESM.zip › Figure 1K/eyesection_rabbitantimitf_mitfsl.png]

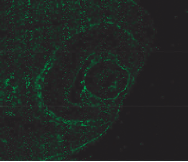

Supplement: Supplementary file 4 — Source data Fig. 1 [file 44319_2024_225_MOESM4_ESM.zip › Figure 1K/eyesections_6A5_mitfsl.png]

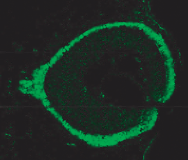

Supplement: Supplementary file 4 — Source data Fig. 1 [file 44319_2024_225_MOESM4_ESM.zip › Figure 1K/eyesections_6A5_mitfsp.png]

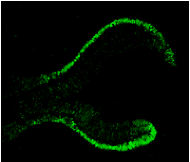

Supplement: Supplementary file 4 — Source data Fig. 1 [file 44319_2024_225_MOESM4_ESM.zip › Figure 1K/eyesections_rabit anitmitf_mitfsp.png]

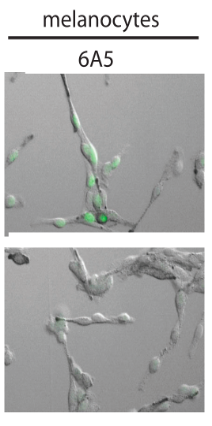

Supplement: Supplementary file 4 — Source data Fig. 1 [file 44319_2024_225_MOESM4_ESM.zip › Figure 1K/Melanocyte 6A5_SPSL.png]
