# Supplementary material for: Novel mechanisms of MITF regulation identified in a mouse suppressor screen
Source: EMBO Rep. 2024 Aug 21;25(10):4252–80. doi: 10.1038/s44319-024-00225-3 (PMC11467436; doi:10.1038/s44319-024-00225-3)
Supplement: Supplementary file 5 — Source data Fig. 2 [file 44319_2024_225_MOESM5_ESM.zip › 2A/Figure 2A.pptx]

## Slide 1
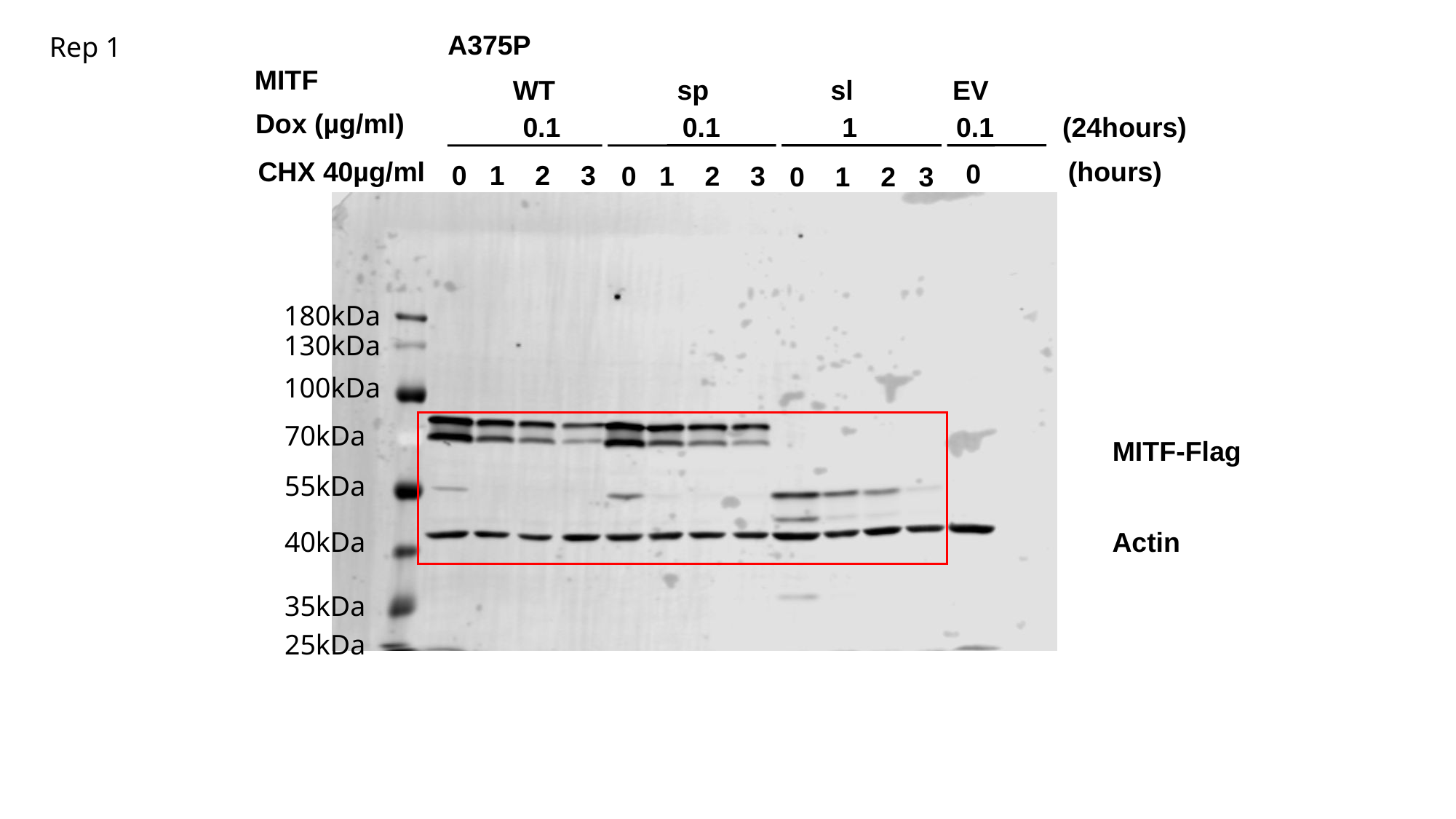

A375P
Rep 1
MITF
 WT sp sl EV
Dox (µg/ml)
 0.1 0.1 1 0.1 (24hours)
CHX 40µg/ml
 (hours)
 0
 0 1 2 3
 0 1 2 3
 0 1 2 3
180kDa
130kDa
100kDa
70kDa
MITF-Flag
55kDa
Actin
40kDa
35kDa
25kDa

## Slide 2
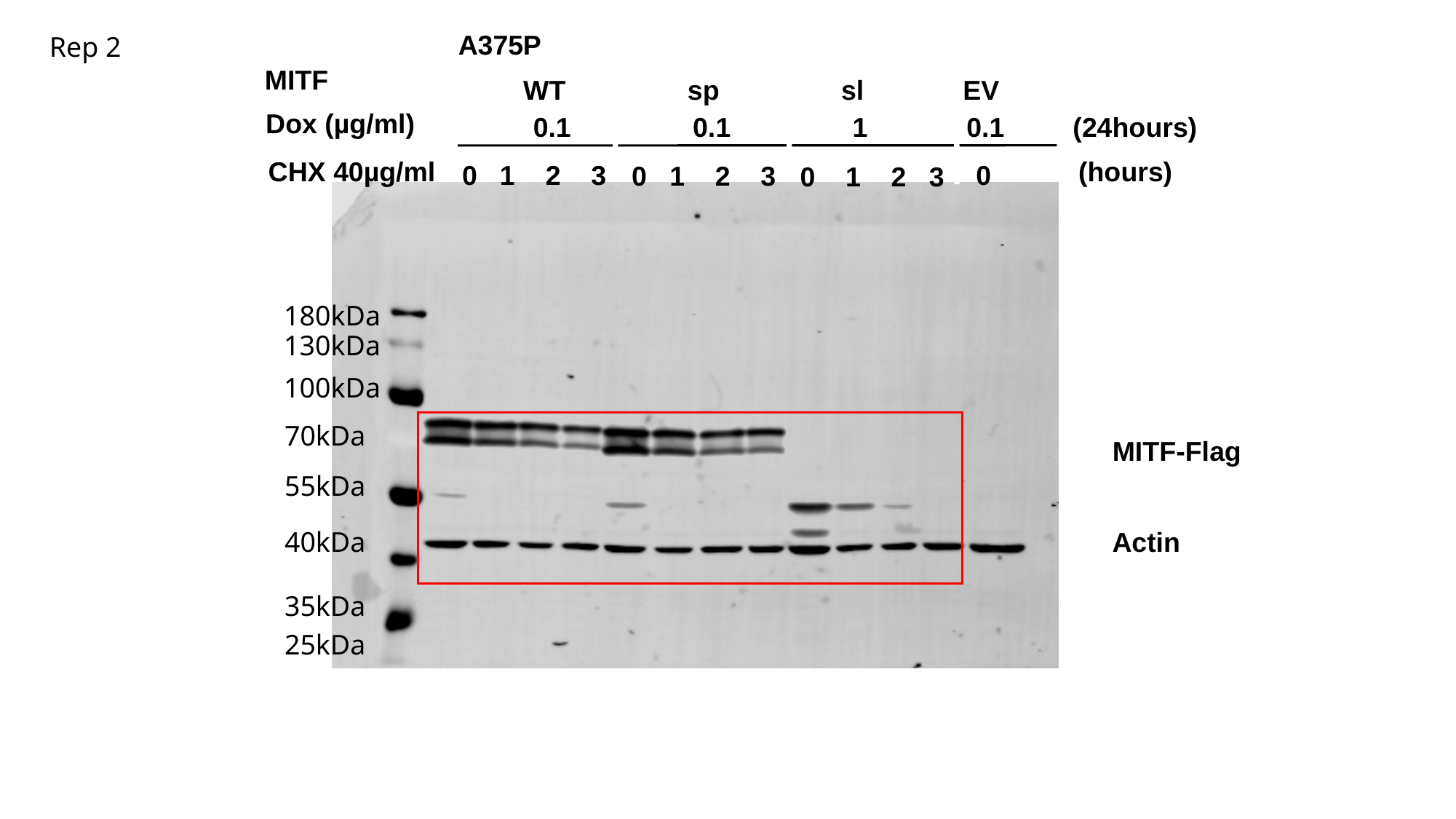

A375P
Rep 2
MITF
 WT sp sl EV
Dox (µg/ml)
 0.1 0.1 1 0.1 (24hours)
CHX 40µg/ml
 (hours)
 0 1 2 3
 0
 0 1 2 3
 0 1 2 3
180kDa
130kDa
100kDa
70kDa
MITF-Flag
55kDa
Actin
40kDa
35kDa
25kDa

## Slide 3
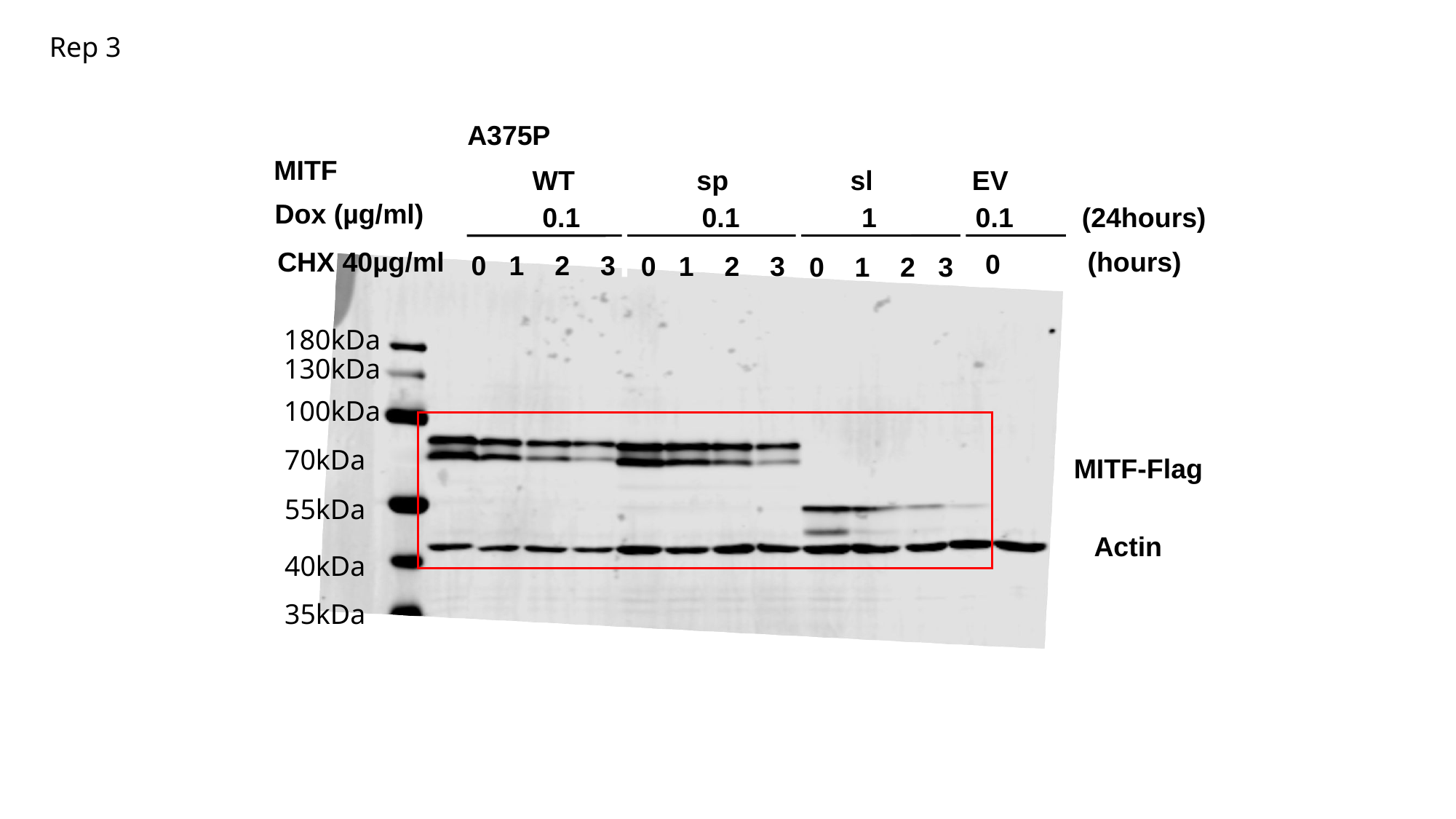

Rep 3
A375P
MITF
 WT sp sl EV
Dox (µg/ml)
 0.1 0.1 1 0.1 (24hours)
CHX 40µg/ml
 (hours)
 0
 0 1 2 3
 0 1 2 3
 0 1 2 3
180kDa
130kDa
100kDa
70kDa
MITF-Flag
55kDa
Actin
40kDa
35kDa

## Slide 4
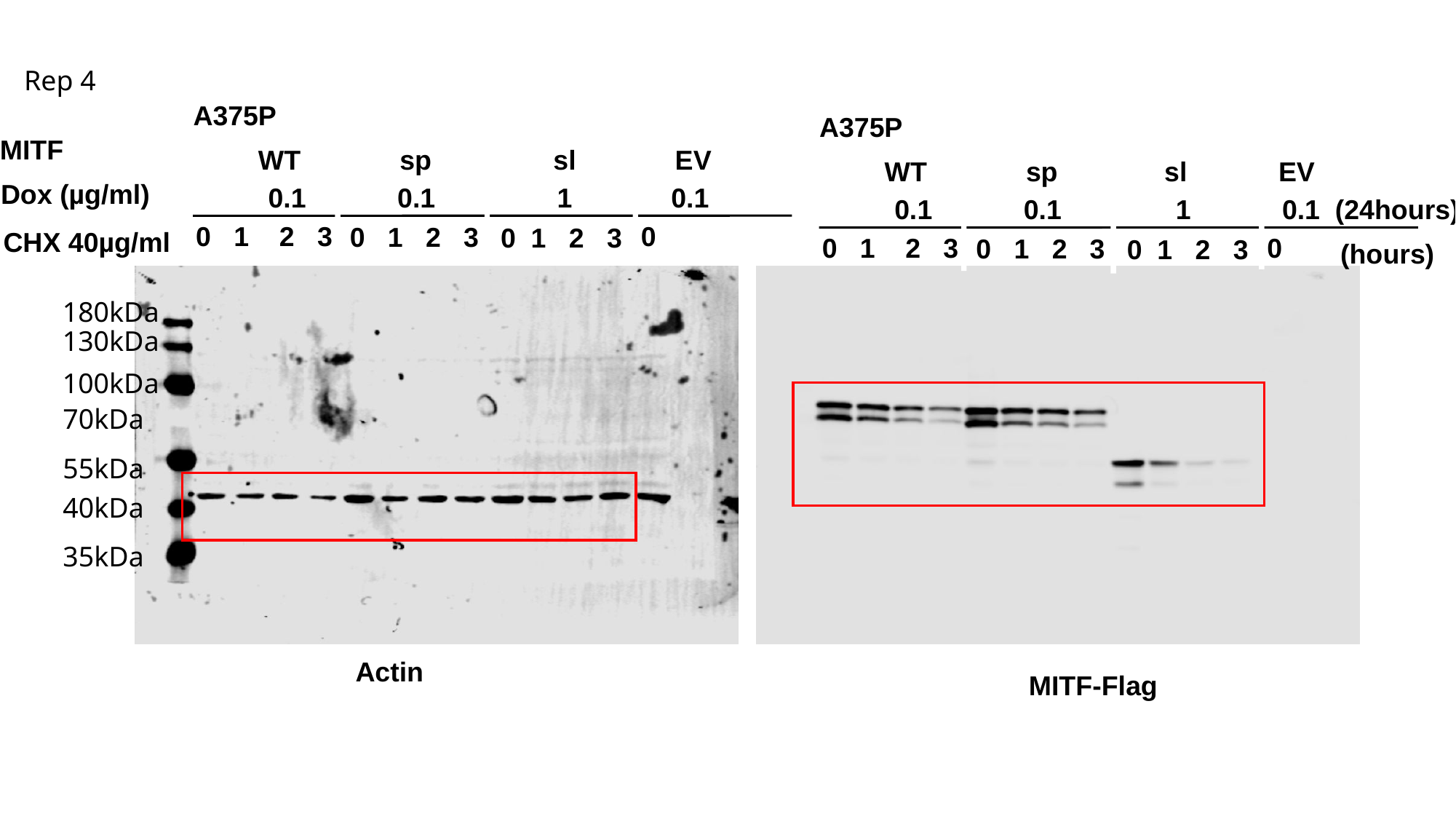

Rep 4
A375P
A375P
MITF
 WT sp sl EV
 WT sp sl EV
Dox (µg/ml)
 0.1 0.1 1 0.1
 0
 0.1 0.1 1 0.1 (24hours)
 0
 0 1 2 3
 0 1 2 3
 0 1 2 3
CHX 40µg/ml
 0 1 2 3
 0 1 2 3
 0 1 2 3
 (hours)
180kDa
130kDa
100kDa
70kDa
55kDa
40kDa
35kDa
Actin
MITF-Flag
