# Supplementary material for: Novel mechanisms of MITF regulation identified in a mouse suppressor screen
Source: EMBO Rep. 2024 Aug 21;25(10):4252–80. doi: 10.1038/s44319-024-00225-3 (PMC11467436; doi:10.1038/s44319-024-00225-3)
Supplement: Supplementary file 5 — Source data Fig. 2 [file 44319_2024_225_MOESM5_ESM.zip › 2C/Figure 2C.pptx]

## Slide 1
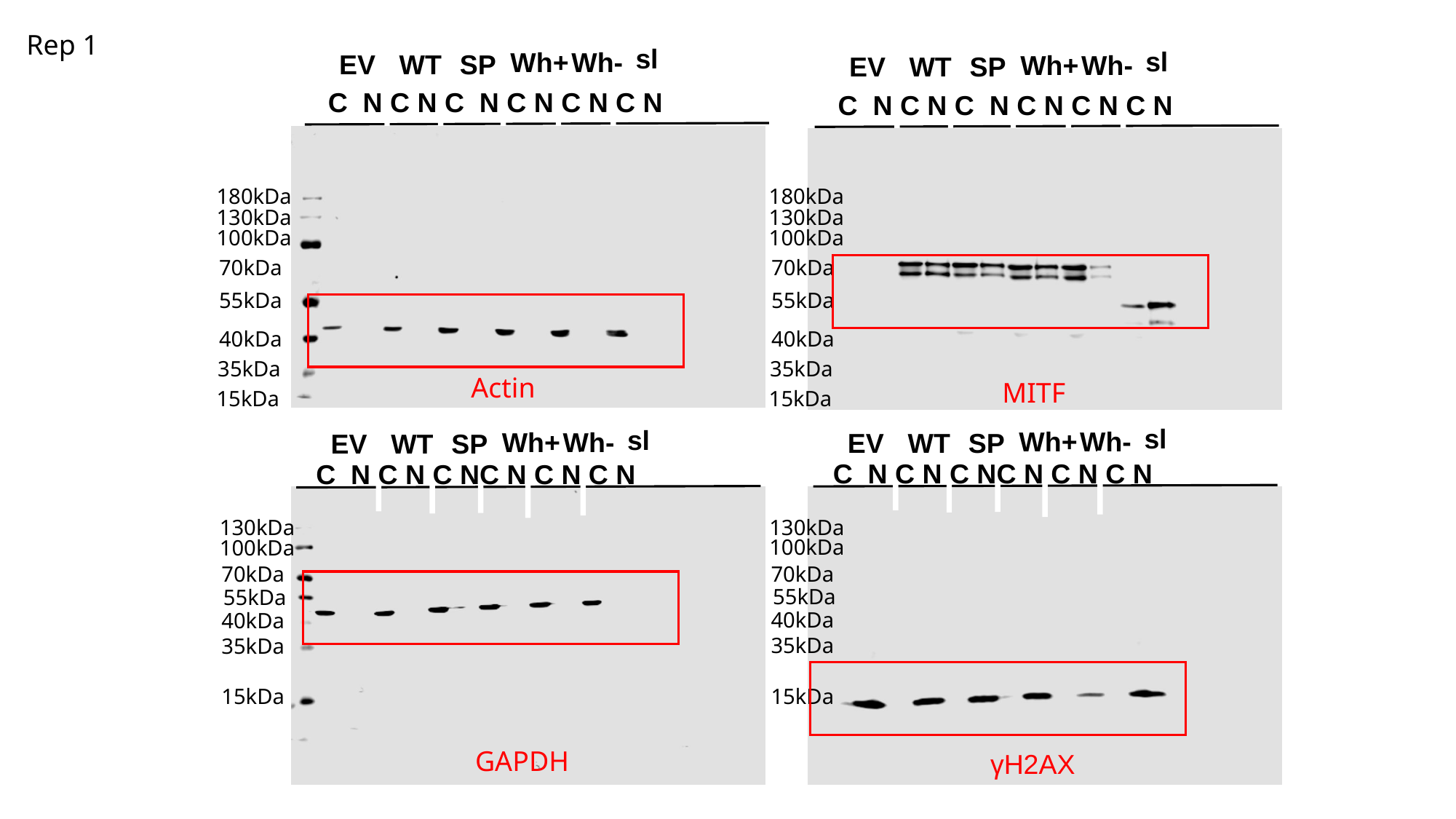

Rep 1
sl
sl
Wh+
Wh-
SP
EV
WT
Wh+
Wh-
SP
EV
WT
C N C N C N C N C N C N
C N C N C N C N C N C N
180kDa
180kDa
130kDa
130kDa
100kDa
100kDa
70kDa
70kDa
55kDa
55kDa
40kDa
40kDa
35kDa
35kDa
Actin
MITF
15kDa
15kDa
sl
sl
Wh+
Wh-
Wh+
Wh-
SP
EV
WT
SP
EV
WT
C N C N C NC N C N C N
C N C N C NC N C N C N
130kDa
130kDa
100kDa
100kDa
70kDa
70kDa
55kDa
55kDa
40kDa
40kDa
35kDa
35kDa
15kDa
15kDa
GAPDH
γH2AX

## Slide 2
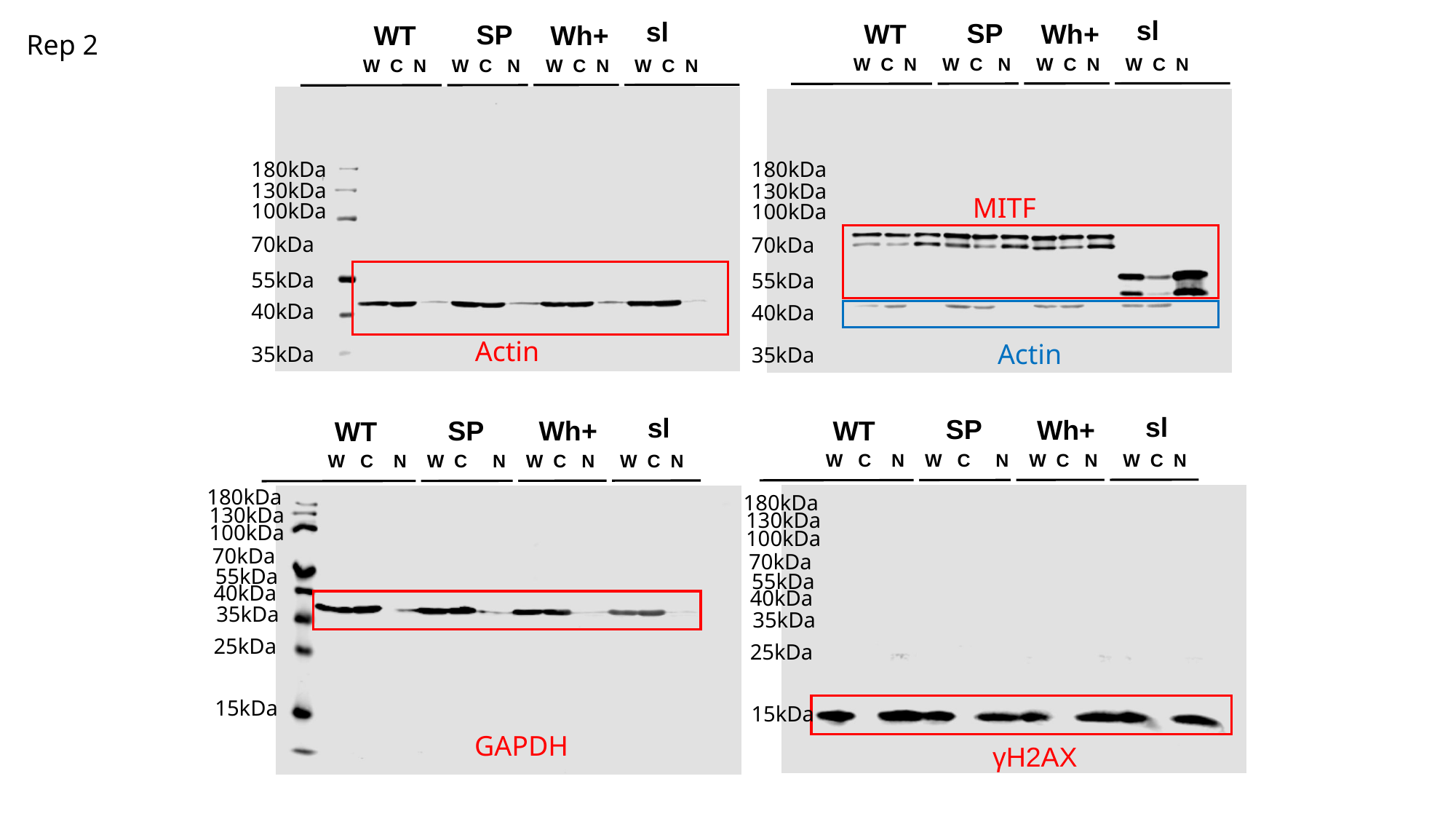

sl
sl
SP
Wh+
WT
SP
Wh+
WT
Rep 2
W C N W C N W C N W C N
W C N W C N W C N W C N
180kDa
180kDa
130kDa
130kDa
MITF
100kDa
100kDa
70kDa
70kDa
55kDa
55kDa
40kDa
40kDa
Actin
Actin
35kDa
35kDa
sl
sl
SP
Wh+
SP
WT
Wh+
WT
W C N W C N W C N W C N
W C N W C N W C N W C N
180kDa
180kDa
130kDa
130kDa
100kDa
100kDa
70kDa
70kDa
55kDa
55kDa
40kDa
40kDa
35kDa
35kDa
25kDa
25kDa
15kDa
15kDa
GAPDH
γH2AX

## Slide 3
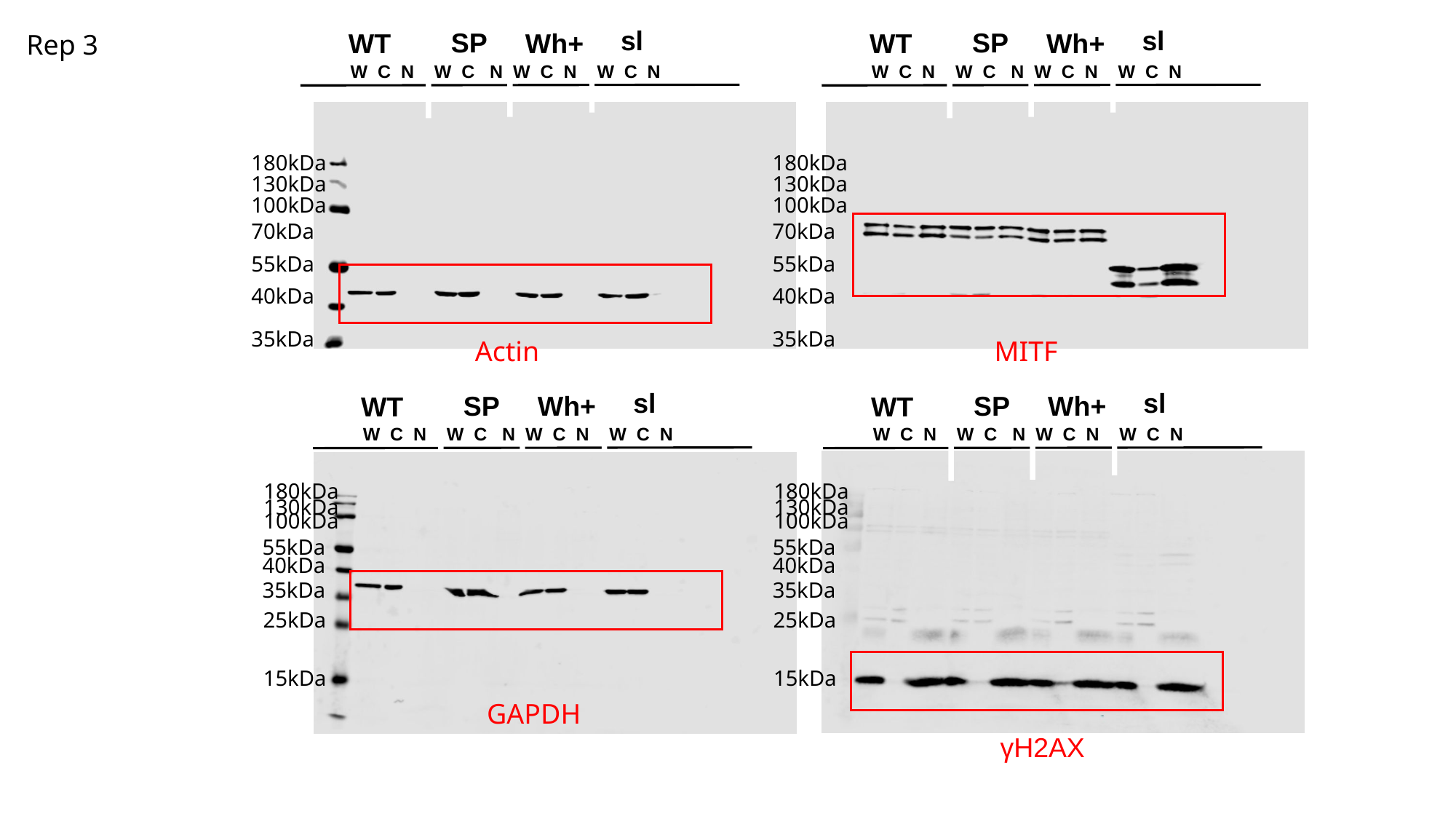

sl
sl
SP
SP
Wh+
Wh+
WT
WT
Rep 3
W C N W C N W C N W C N
W C N W C N W C N W C N
180kDa
180kDa
130kDa
130kDa
100kDa
100kDa
70kDa
70kDa
55kDa
55kDa
40kDa
40kDa
35kDa
35kDa
Actin
MITF
sl
sl
SP
SP
Wh+
Wh+
WT
WT
W C N W C N W C N W C N
W C N W C N W C N W C N
180kDa
180kDa
130kDa
130kDa
100kDa
100kDa
55kDa
55kDa
40kDa
40kDa
35kDa
35kDa
25kDa
25kDa
15kDa
15kDa
GAPDH
γH2AX
