# Supplementary material for: Novel mechanisms of MITF regulation identified in a mouse suppressor screen
Source: EMBO Rep. 2024 Aug 21;25(10):4252–80. doi: 10.1038/s44319-024-00225-3 (PMC11467436; doi:10.1038/s44319-024-00225-3)
Supplement: Supplementary file 5 — Source data Fig. 2 [file 44319_2024_225_MOESM5_ESM.zip › 2H/Figure 2H.pptx]

## Slide 1
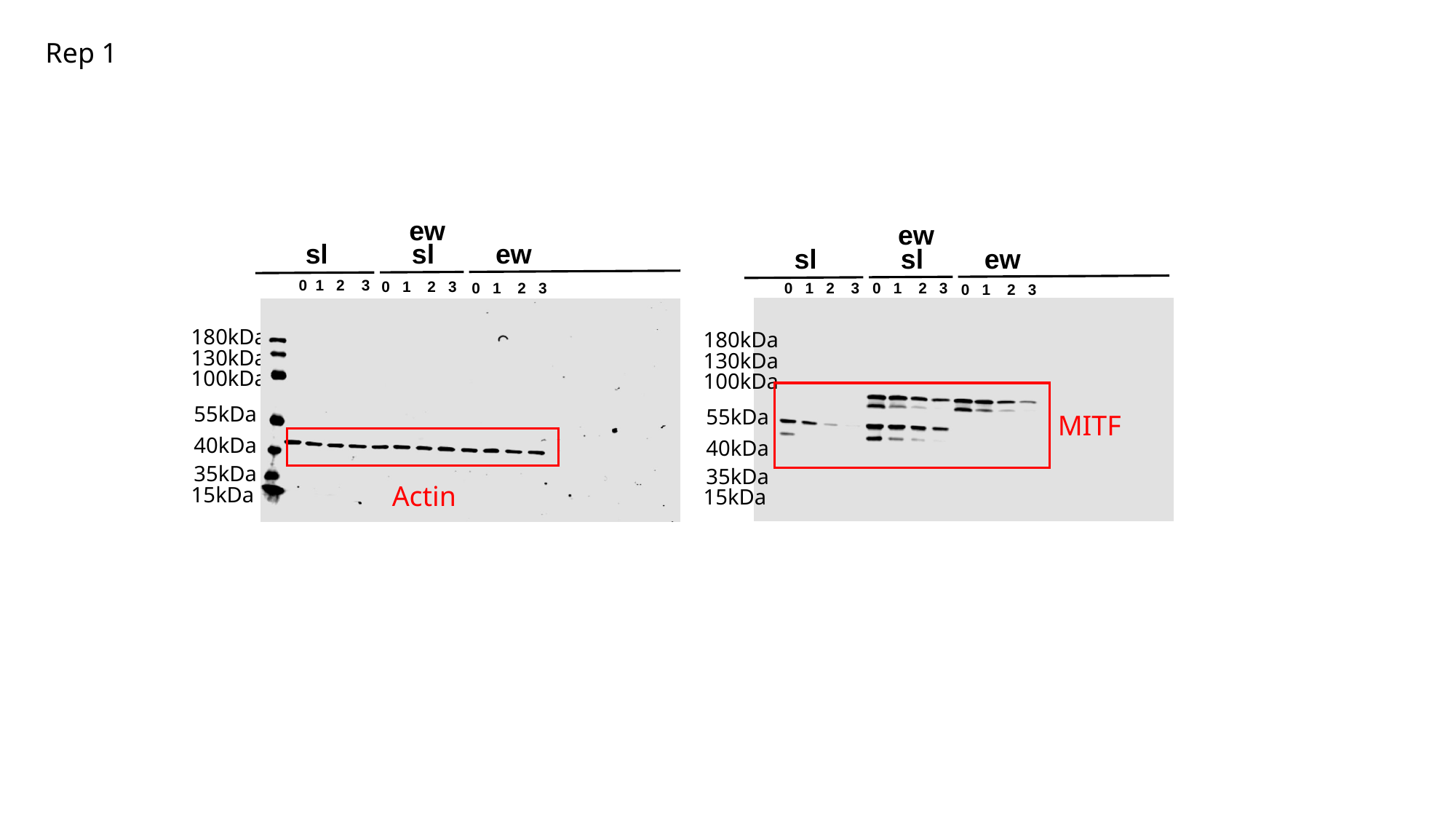

Rep 1
ew
ew
sl sl ew
sl sl ew
 0 1 2 3
 0 1 2 3
 0 1 2 3
 0 1 2 3
 0 1 2 3
 0 1 2 3
180kDa
180kDa
130kDa
130kDa
100kDa
100kDa
55kDa
55kDa
MITF
40kDa
40kDa
35kDa
35kDa
Actin
15kDa
15kDa

## Slide 2
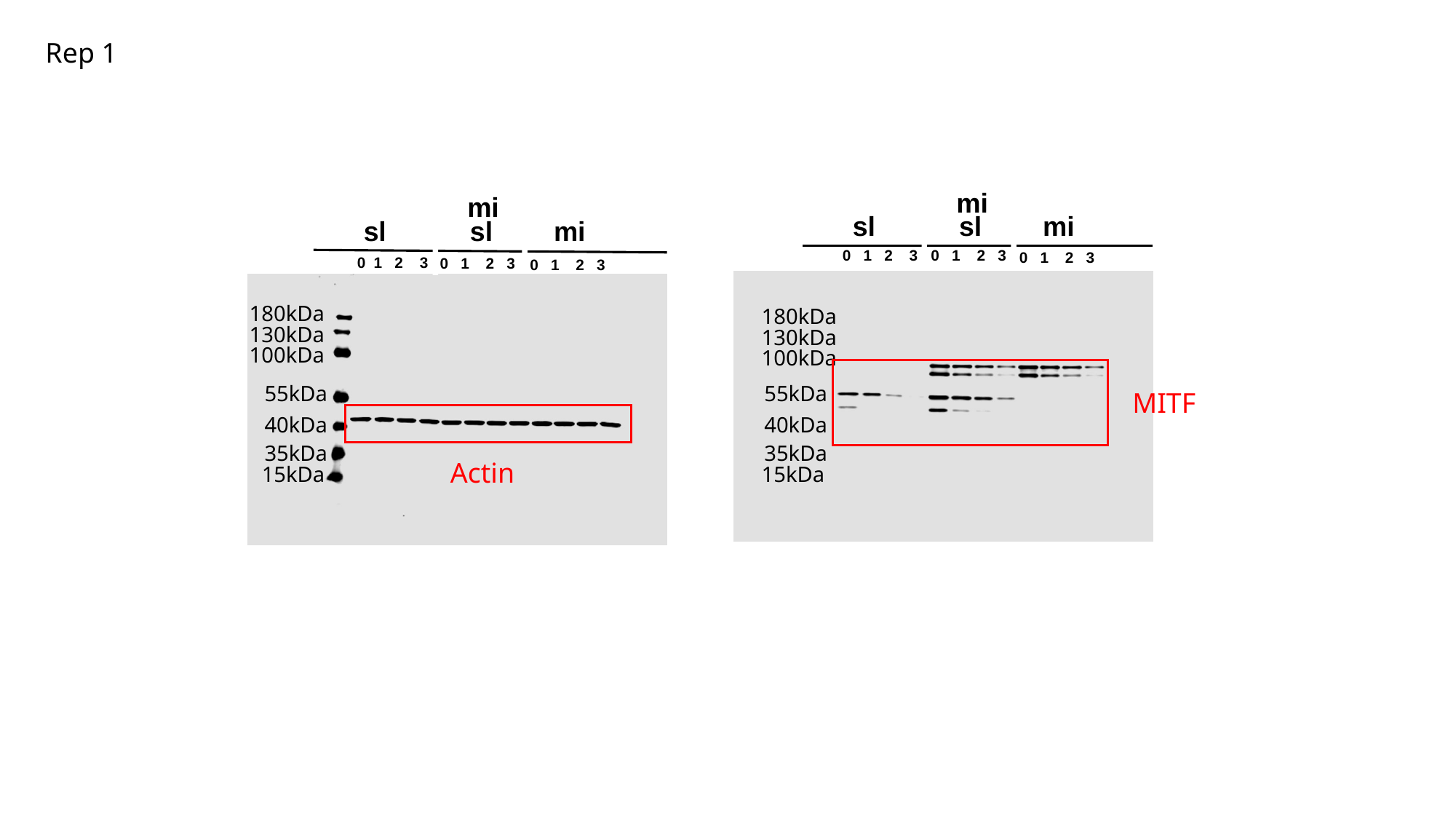

Rep 1
mi
mi
sl sl mi
sl sl mi
 0 1 2 3
 0 1 2 3
 0 1 2 3
 0 1 2 3
 0 1 2 3
 0 1 2 3
180kDa
180kDa
130kDa
130kDa
100kDa
100kDa
55kDa
55kDa
MITF
40kDa
40kDa
35kDa
35kDa
Actin
15kDa
15kDa

## Slide 3
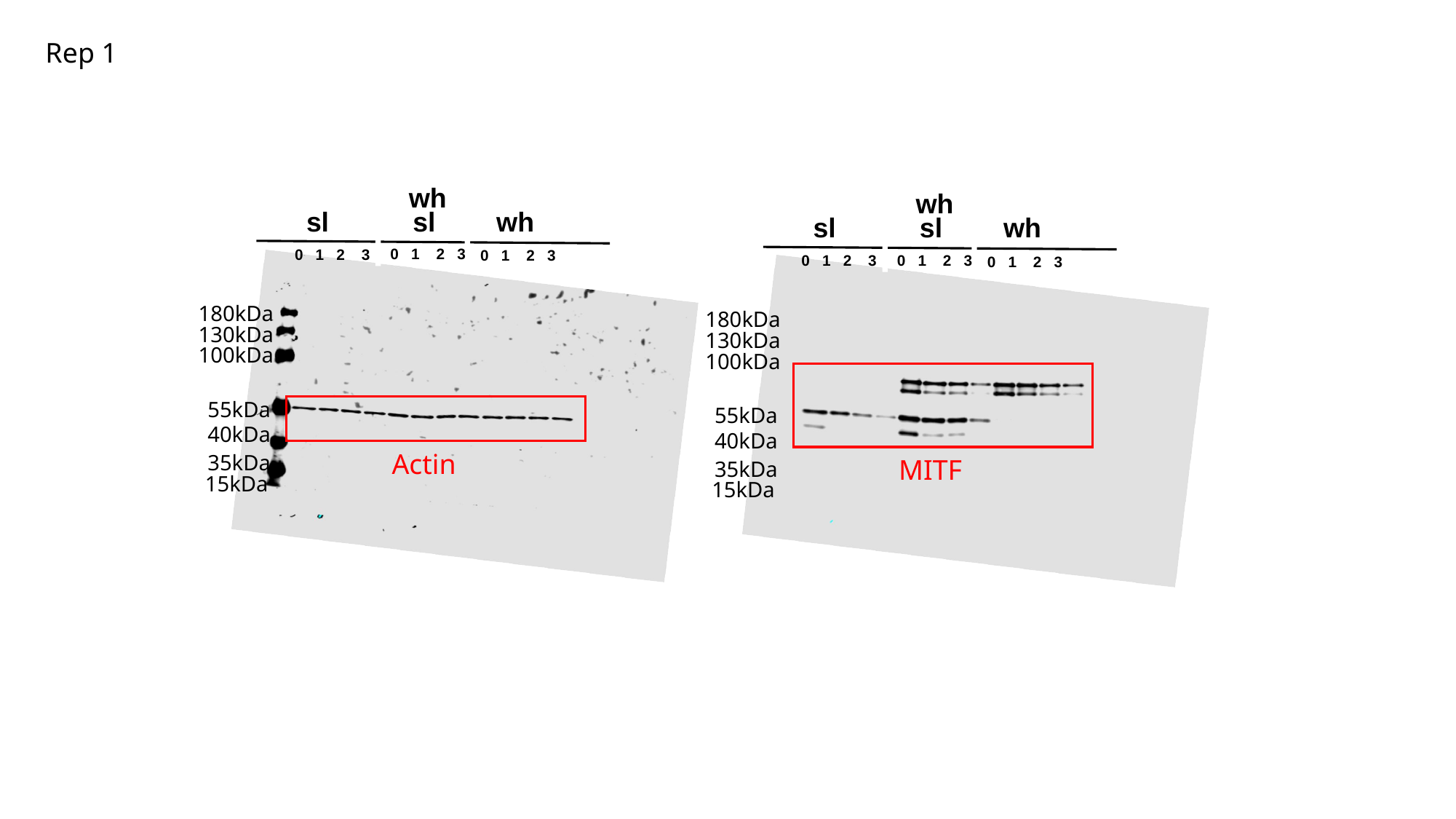

Rep 1
wh
wh
sl sl wh
sl sl wh
 0 1 2 3
 0 1 2 3
 0 1 2 3
 0 1 2 3
 0 1 2 3
 0 1 2 3
180kDa
180kDa
130kDa
130kDa
100kDa
100kDa
55kDa
55kDa
40kDa
40kDa
Actin
35kDa
MITF
35kDa
15kDa
15kDa

## Slide 4
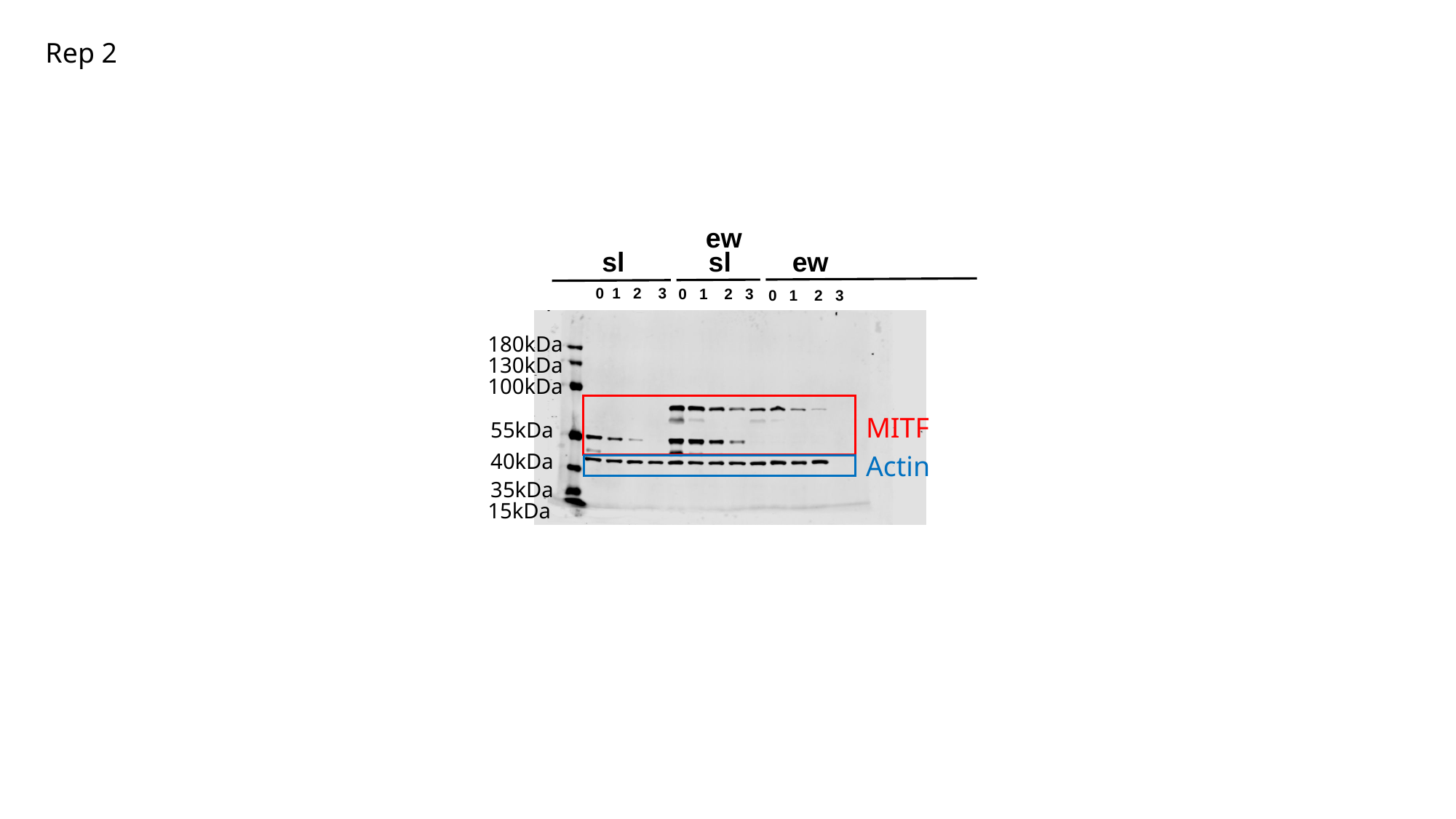

Rep 2
ew
sl sl ew
 0 1 2 3
 0 1 2 3
 0 1 2 3
180kDa
130kDa
100kDa
MITF
55kDa
40kDa
Actin
35kDa
15kDa

## Slide 5
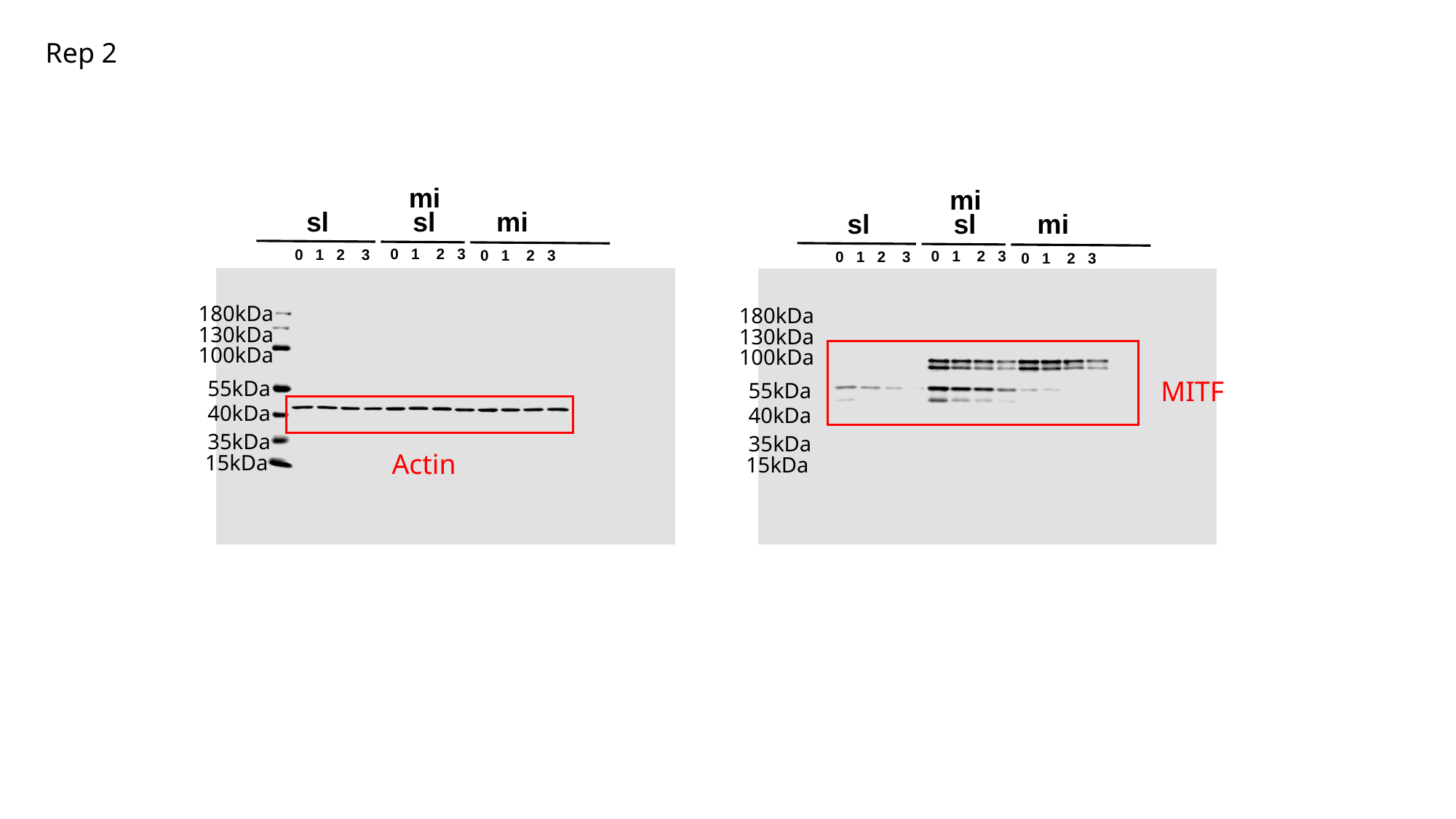

Rep 2
mi
mi
sl sl mi
sl sl mi
 0 1 2 3
 0 1 2 3
 0 1 2 3
 0 1 2 3
 0 1 2 3
 0 1 2 3
180kDa
180kDa
130kDa
130kDa
100kDa
100kDa
MITF
55kDa
55kDa
40kDa
40kDa
35kDa
35kDa
Actin
15kDa
15kDa

## Slide 6
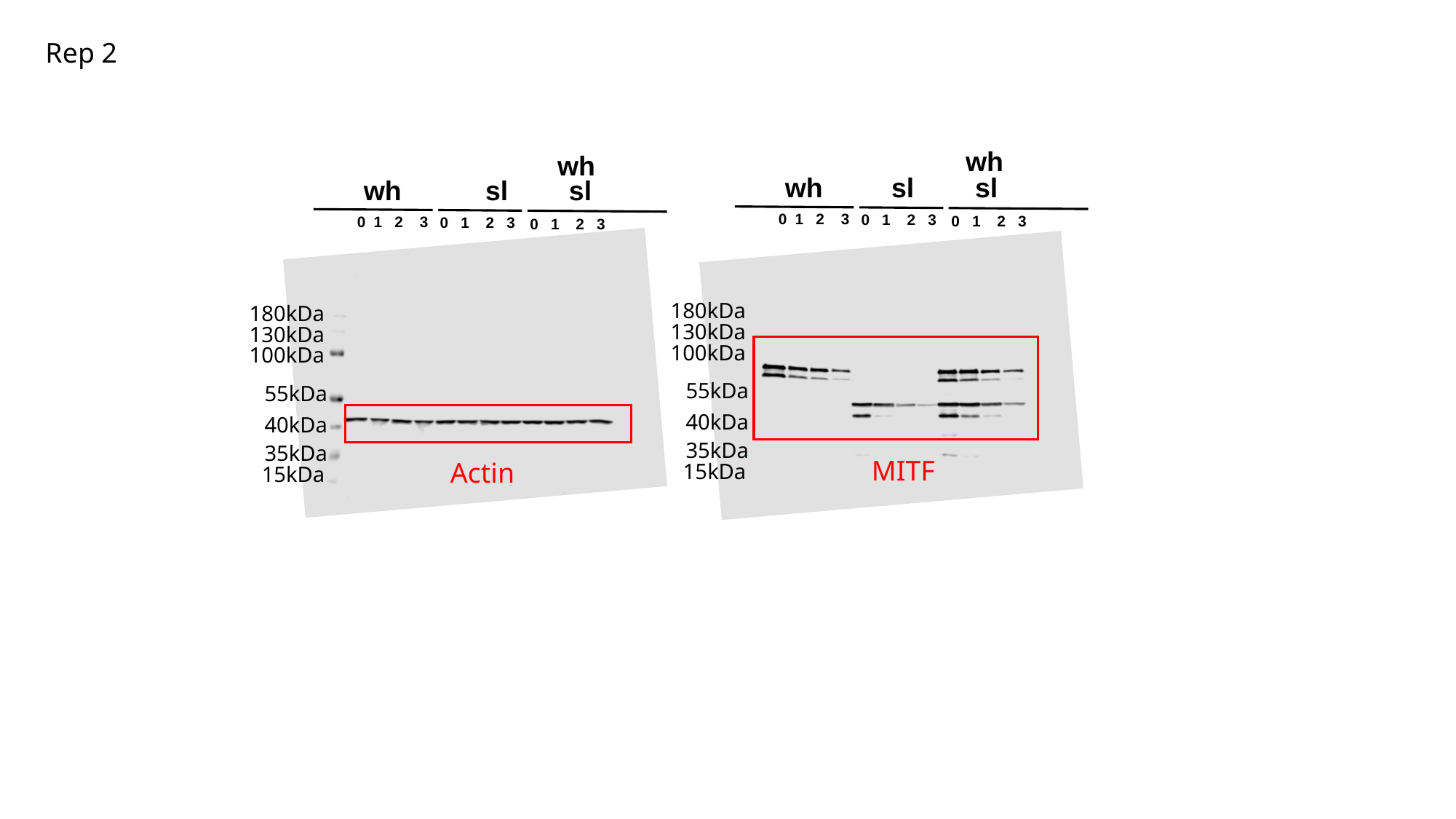

Rep 2
wh
wh
wh sl sl
wh sl sl
 0 1 2 3
 0 1 2 3
 0 1 2 3
 0 1 2 3
 0 1 2 3
 0 1 2 3
180kDa
180kDa
130kDa
130kDa
100kDa
100kDa
55kDa
55kDa
40kDa
40kDa
35kDa
35kDa
MITF
Actin
15kDa
15kDa

## Slide 7
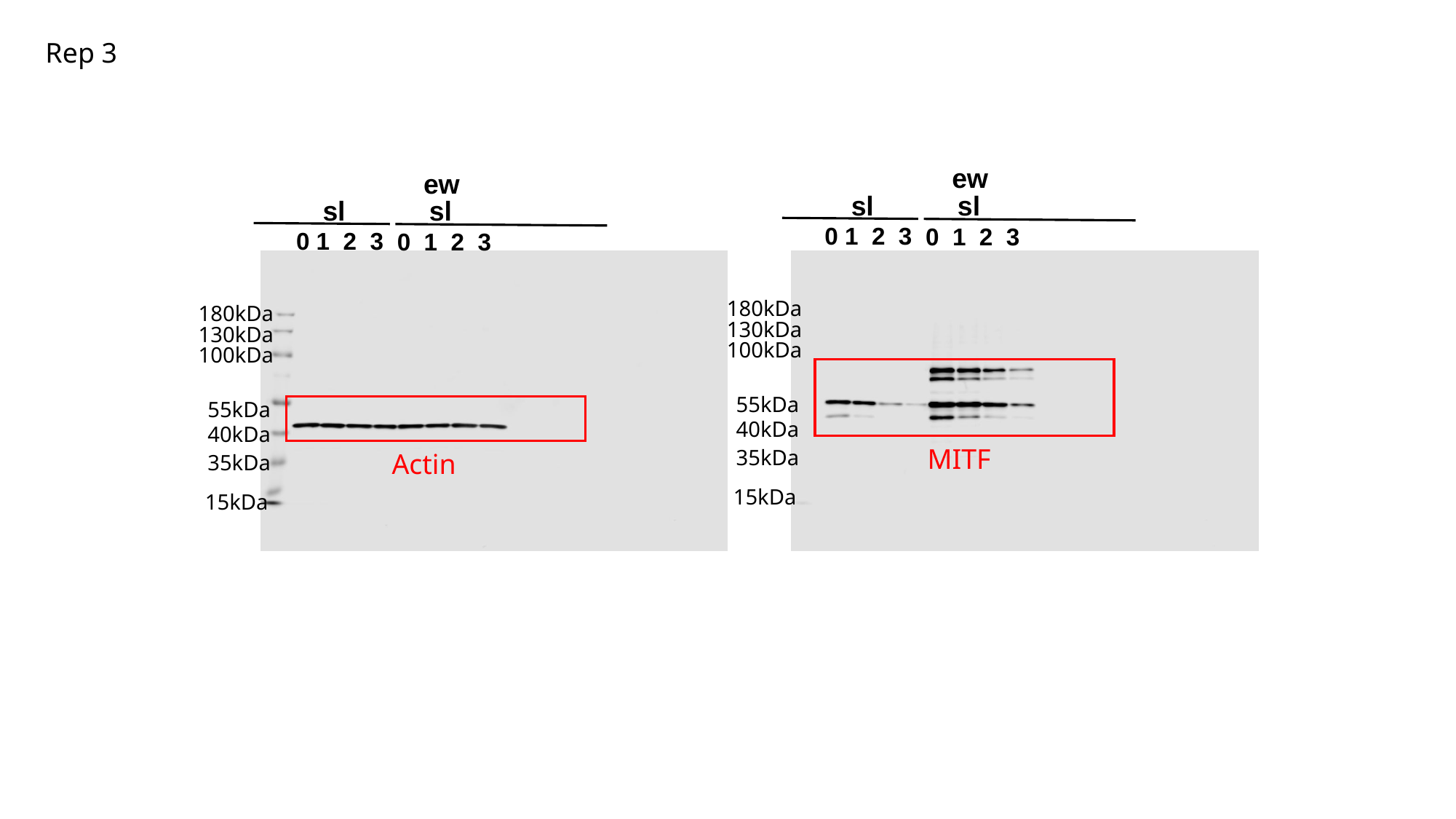

Rep 3
ew
ew
sl sl
sl sl
 0 1 2 3
 0 1 2 3
 0 1 2 3
 0 1 2 3
180kDa
180kDa
130kDa
130kDa
100kDa
100kDa
55kDa
55kDa
40kDa
40kDa
 MITF
35kDa
Actin
35kDa
15kDa
15kDa

## Slide 8
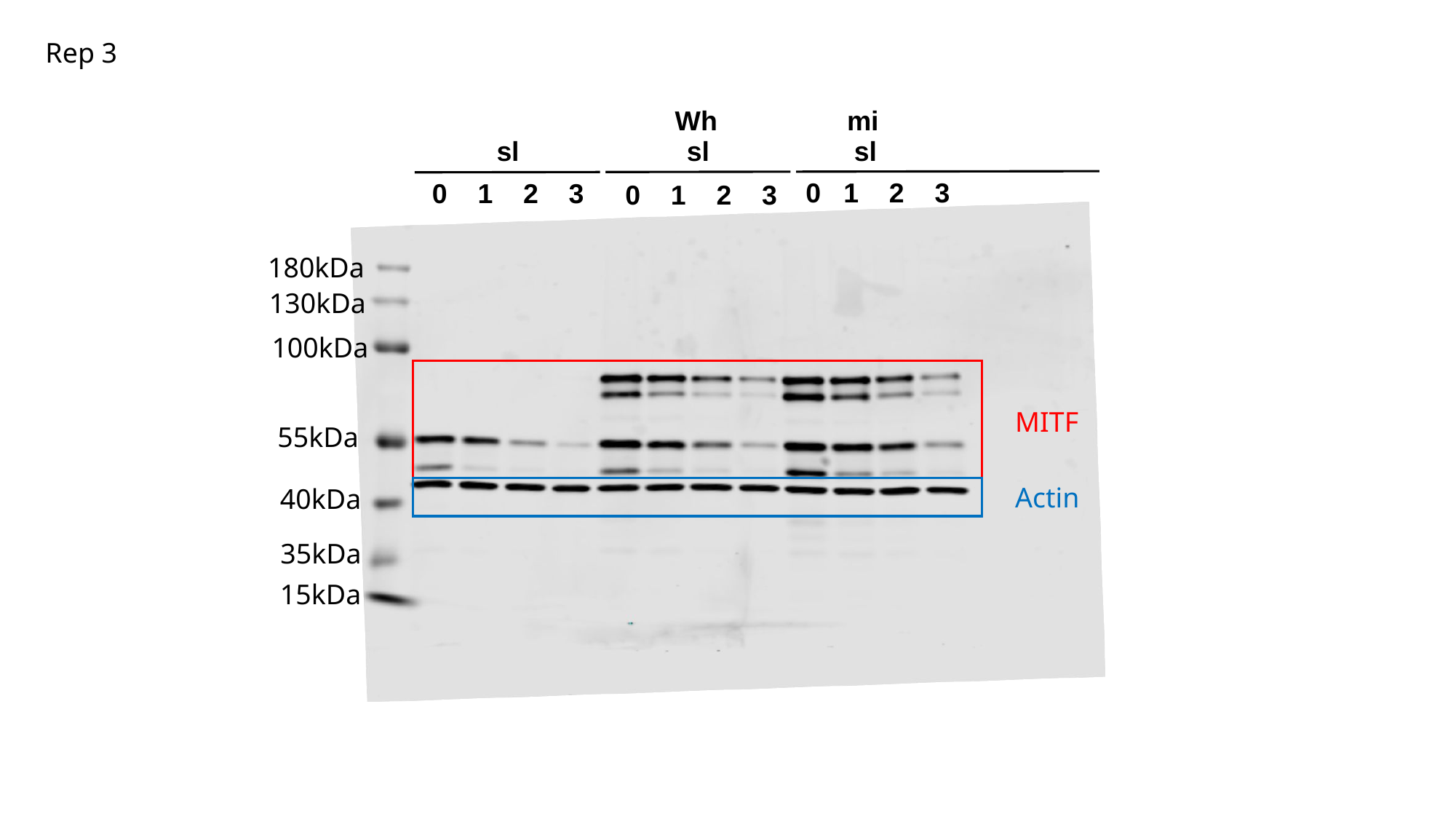

Rep 3
Wh mi
sl sl sl
 0 1 2 3
 0 1 2 3
 0 1 2 3
180kDa
130kDa
100kDa
MITF
55kDa
Actin
40kDa
35kDa
15kDa
