# Supplementary material for: Novel mechanisms of MITF regulation identified in a mouse suppressor screen
Source: EMBO Rep. 2024 Aug 21;25(10):4252–80. doi: 10.1038/s44319-024-00225-3 (PMC11467436; doi:10.1038/s44319-024-00225-3)
Supplement: Supplementary file 5 — Source data Fig. 2 [file 44319_2024_225_MOESM5_ESM.zip › 2E/Figure 2E.pptx]

## Slide 1
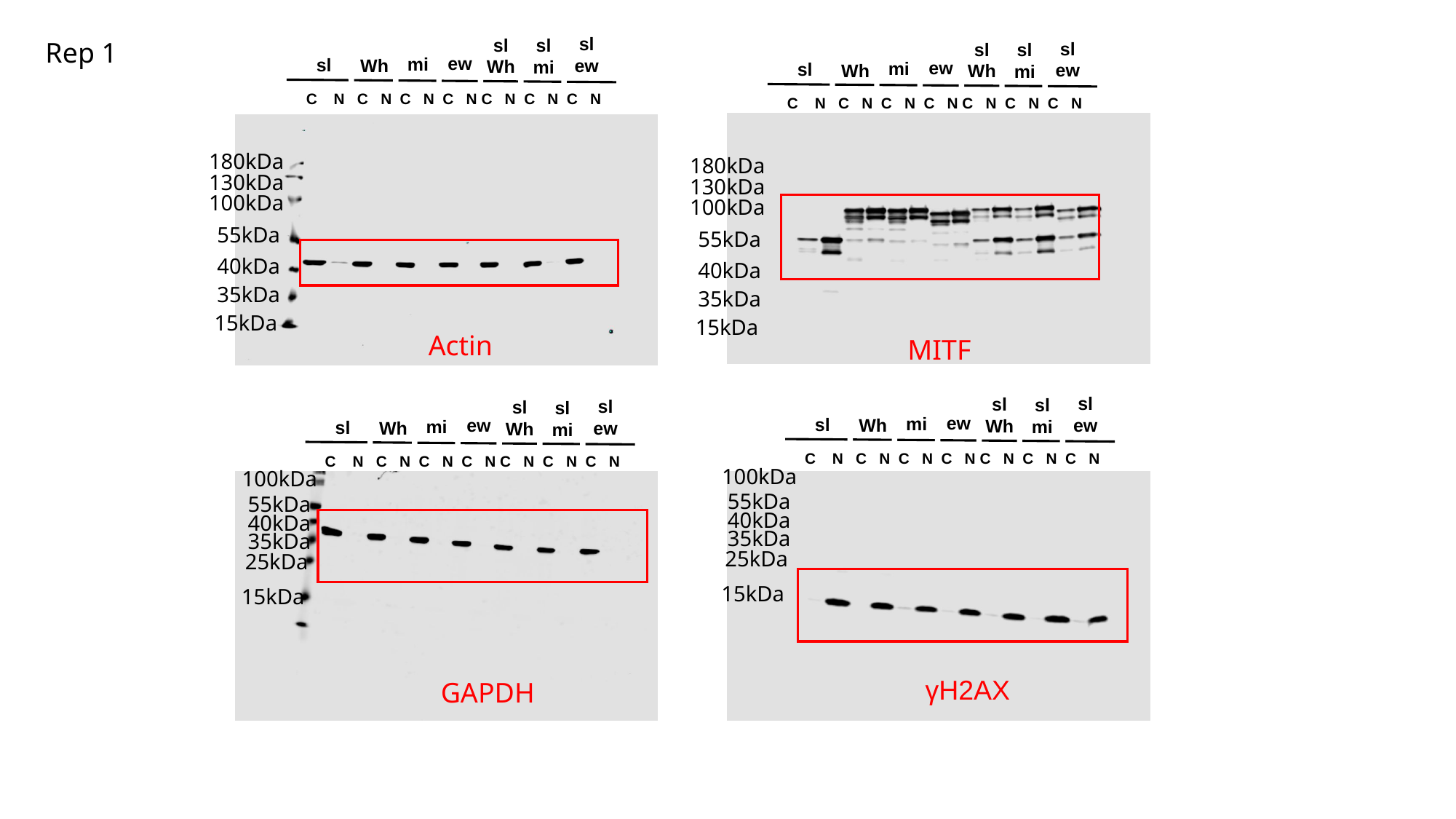

sl
ew
sl
Wh
sl
mi
Rep 1
sl
ew
sl
Wh
sl
mi
ew
mi
sl
Wh
ew
mi
sl
Wh
C N C N C N C N C N C N C N
C N C N C N C N C N C N C N
180kDa
180kDa
130kDa
130kDa
100kDa
100kDa
55kDa
55kDa
40kDa
40kDa
35kDa
35kDa
15kDa
15kDa
Actin
MITF
sl
ew
sl
Wh
sl
mi
sl
ew
sl
Wh
sl
mi
ew
mi
sl
Wh
ew
mi
sl
Wh
C N C N C N C N C N C N C N
C N C N C N C N C N C N C N
100kDa
100kDa
55kDa
55kDa
40kDa
40kDa
35kDa
35kDa
25kDa
25kDa
15kDa
15kDa
γH2AX
GAPDH

## Slide 2
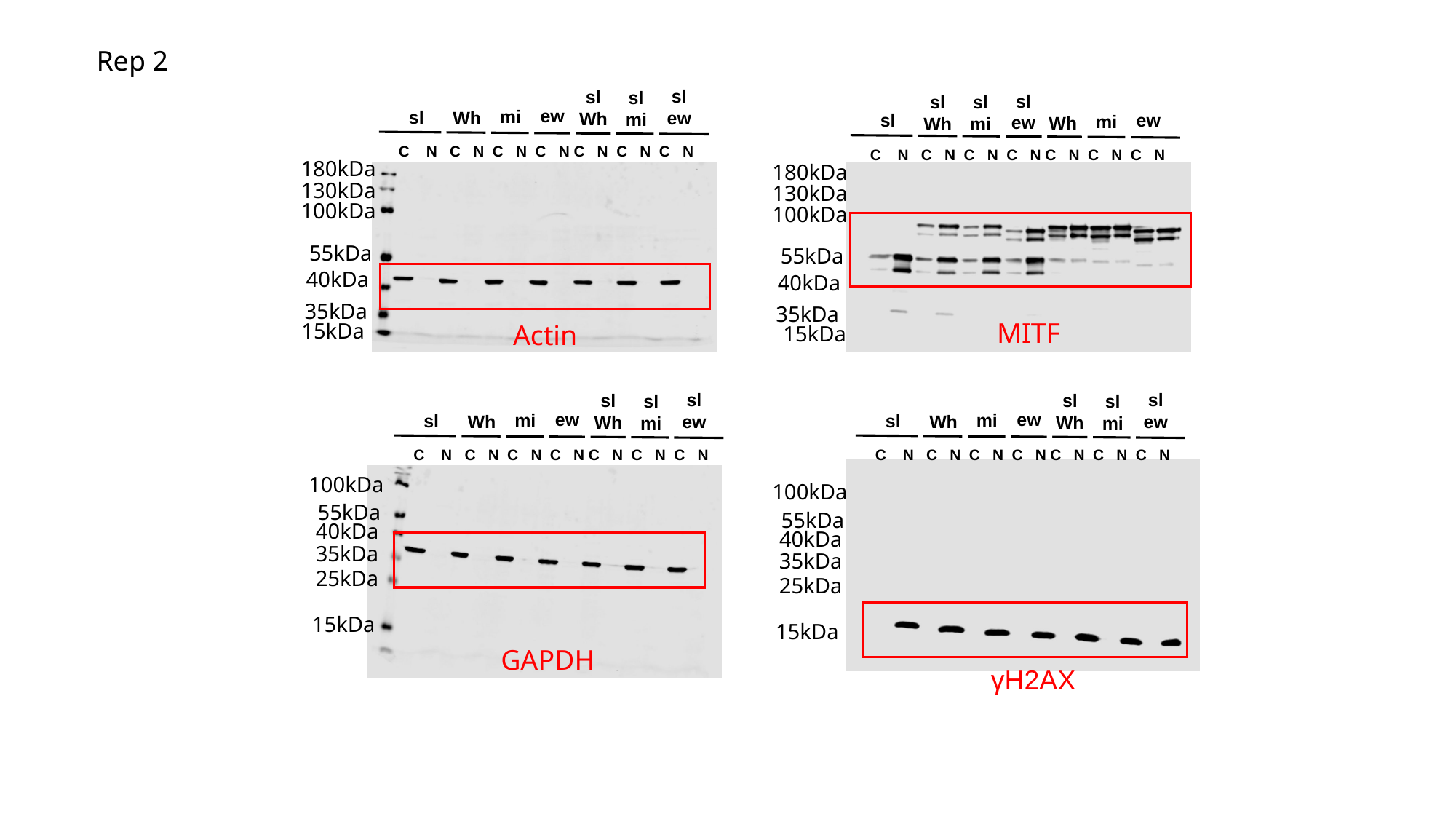

Rep 2
sl
ew
sl
Wh
sl
mi
sl
ew
sl
Wh
sl
mi
ew
mi
sl
Wh
ew
sl
mi
Wh
C N C N C N C N C N C N C N
C N C N C N C N C N C N C N
180kDa
180kDa
130kDa
130kDa
100kDa
100kDa
55kDa
55kDa
40kDa
40kDa
35kDa
35kDa
MITF
15kDa
Actin
15kDa
sl
ew
sl
ew
sl
Wh
sl
Wh
sl
mi
sl
mi
ew
ew
mi
mi
sl
sl
Wh
Wh
C N C N C N C N C N C N C N
C N C N C N C N C N C N C N
100kDa
100kDa
55kDa
55kDa
40kDa
40kDa
35kDa
35kDa
25kDa
25kDa
15kDa
15kDa
GAPDH
γH2AX

## Slide 3
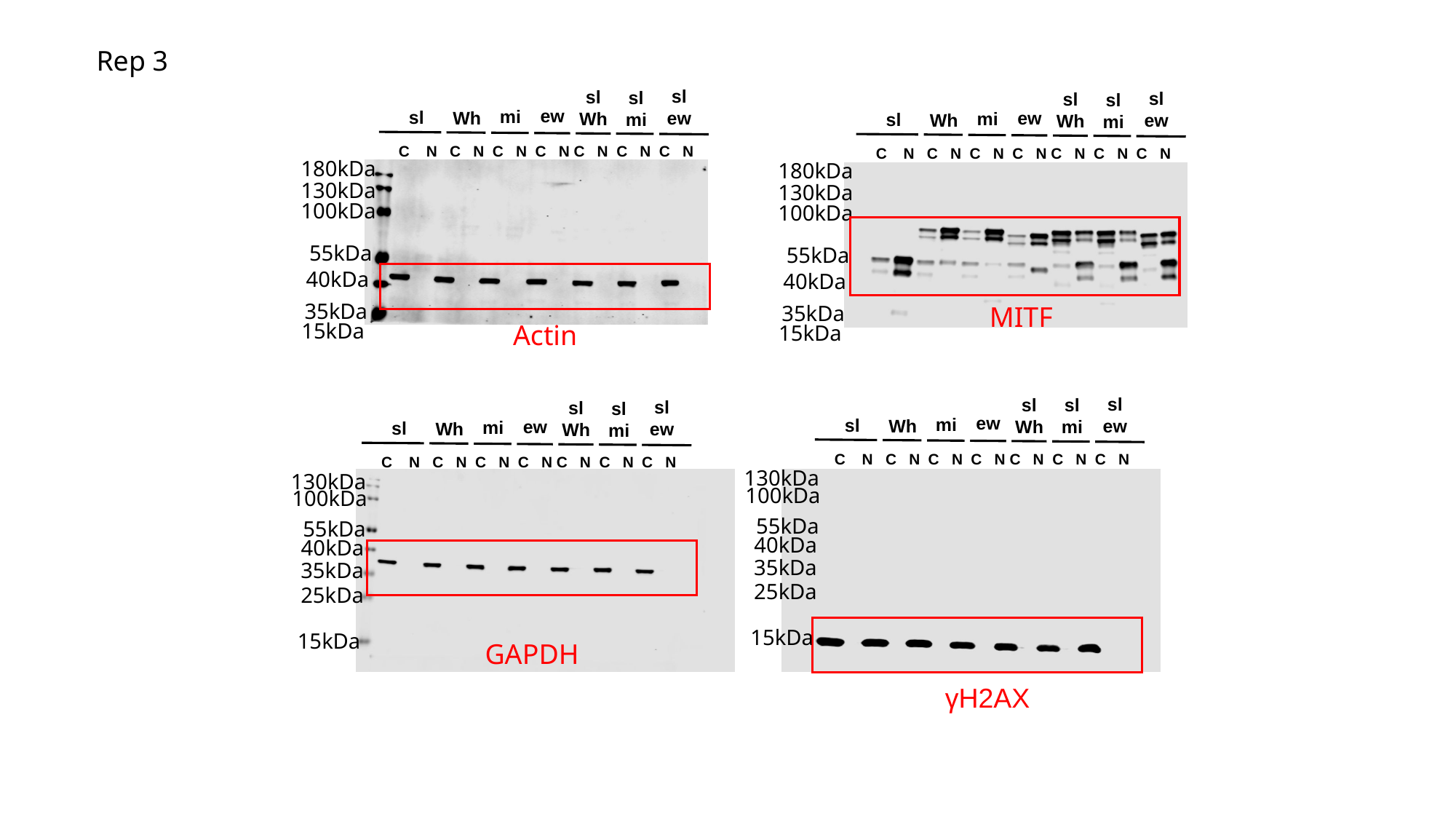

Rep 3
sl
ew
sl
Wh
sl
mi
sl
ew
sl
Wh
sl
mi
ew
mi
sl
ew
Wh
mi
sl
Wh
C N C N C N C N C N C N C N
C N C N C N C N C N C N C N
180kDa
180kDa
130kDa
130kDa
100kDa
100kDa
55kDa
55kDa
40kDa
40kDa
35kDa
35kDa
MITF
15kDa
Actin
15kDa
sl
ew
sl
Wh
sl
mi
sl
ew
sl
Wh
sl
mi
ew
mi
sl
Wh
ew
mi
sl
Wh
C N C N C N C N C N C N C N
C N C N C N C N C N C N C N
130kDa
130kDa
100kDa
100kDa
55kDa
55kDa
40kDa
40kDa
35kDa
35kDa
25kDa
25kDa
15kDa
15kDa
GAPDH
γH2AX

## Slide 4
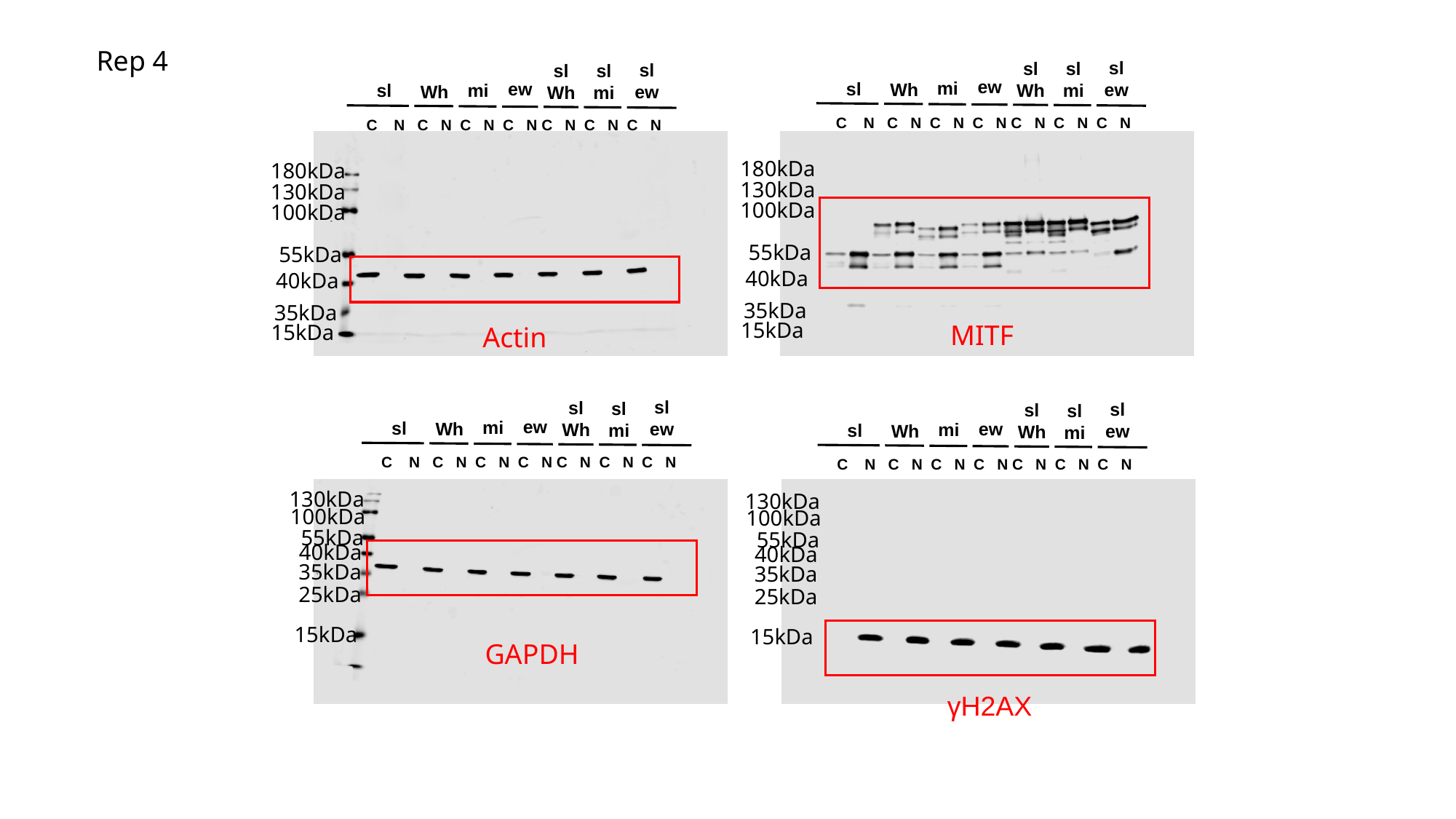

Rep 4
sl
ew
sl
Wh
sl
mi
sl
ew
sl
Wh
sl
mi
ew
mi
sl
ew
Wh
mi
sl
Wh
C N C N C N C N C N C N C N
C N C N C N C N C N C N C N
180kDa
180kDa
130kDa
130kDa
100kDa
100kDa
55kDa
55kDa
40kDa
40kDa
35kDa
35kDa
15kDa
MITF
15kDa
Actin
sl
ew
sl
Wh
sl
mi
sl
ew
sl
Wh
sl
mi
ew
mi
sl
ew
Wh
mi
sl
Wh
C N C N C N C N C N C N C N
C N C N C N C N C N C N C N
130kDa
130kDa
100kDa
100kDa
55kDa
55kDa
40kDa
40kDa
35kDa
35kDa
25kDa
25kDa
15kDa
15kDa
GAPDH
γH2AX
