# Supplementary material for: Novel mechanisms of MITF regulation identified in a mouse suppressor screen
Source: EMBO Rep. 2024 Aug 21;25(10):4252–80. doi: 10.1038/s44319-024-00225-3 (PMC11467436; doi:10.1038/s44319-024-00225-3)
Supplement: Supplementary file 6 — Source data Fig. 3 [file 44319_2024_225_MOESM6_ESM.zip › 3F/Figure 3F.pptx]

## Slide 1
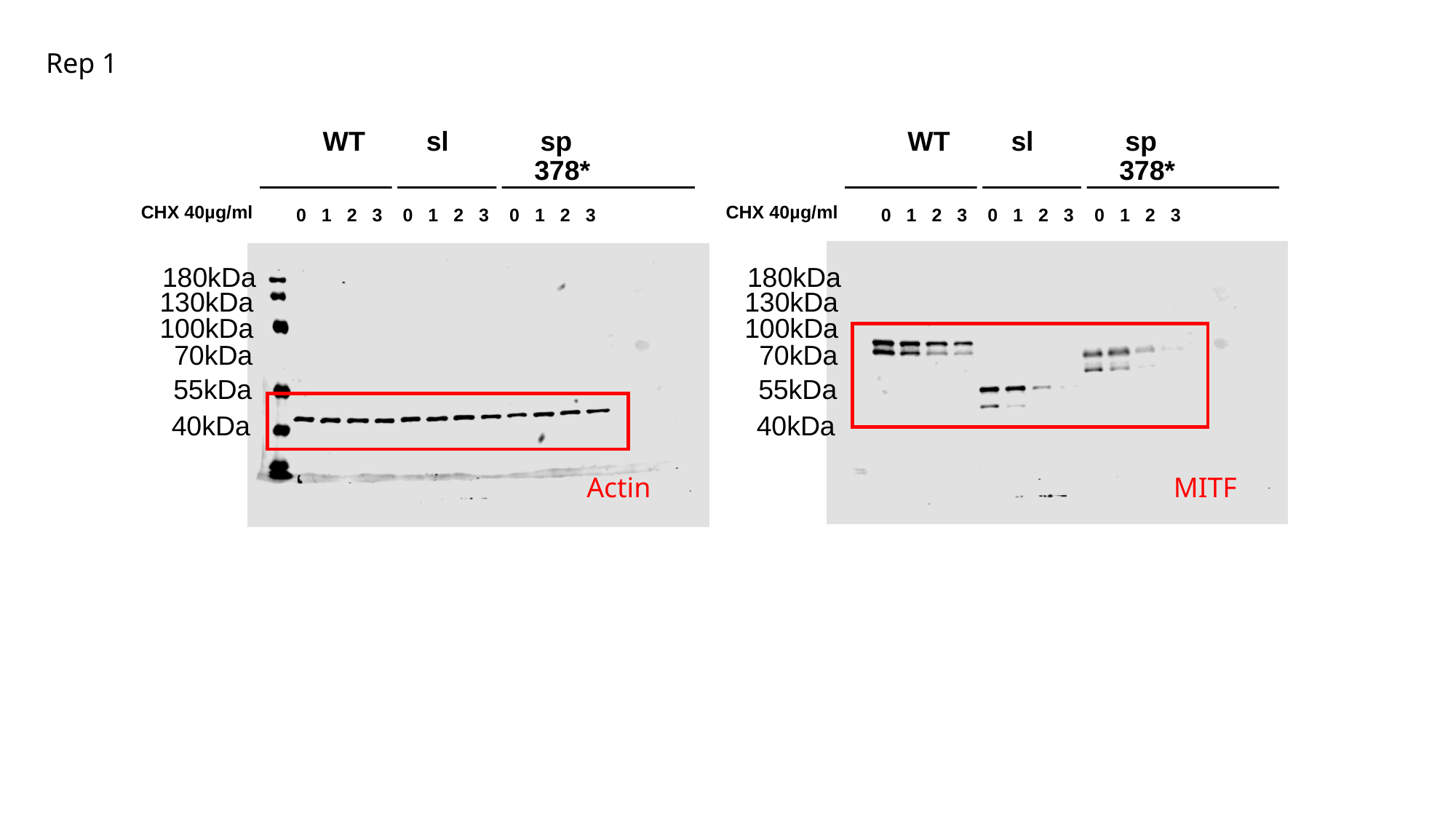

Rep 1
WT sl sp
WT sl sp
378*
378*
CHX 40µg/ml
CHX 40µg/ml
 0 1 2 3
 0 1 2 3
 0 1 2 3
 0 1 2 3
 0 1 2 3
 0 1 2 3
180kDa
180kDa
130kDa
130kDa
100kDa
100kDa
70kDa
70kDa
55kDa
55kDa
40kDa
40kDa
Actin
MITF

## Slide 2
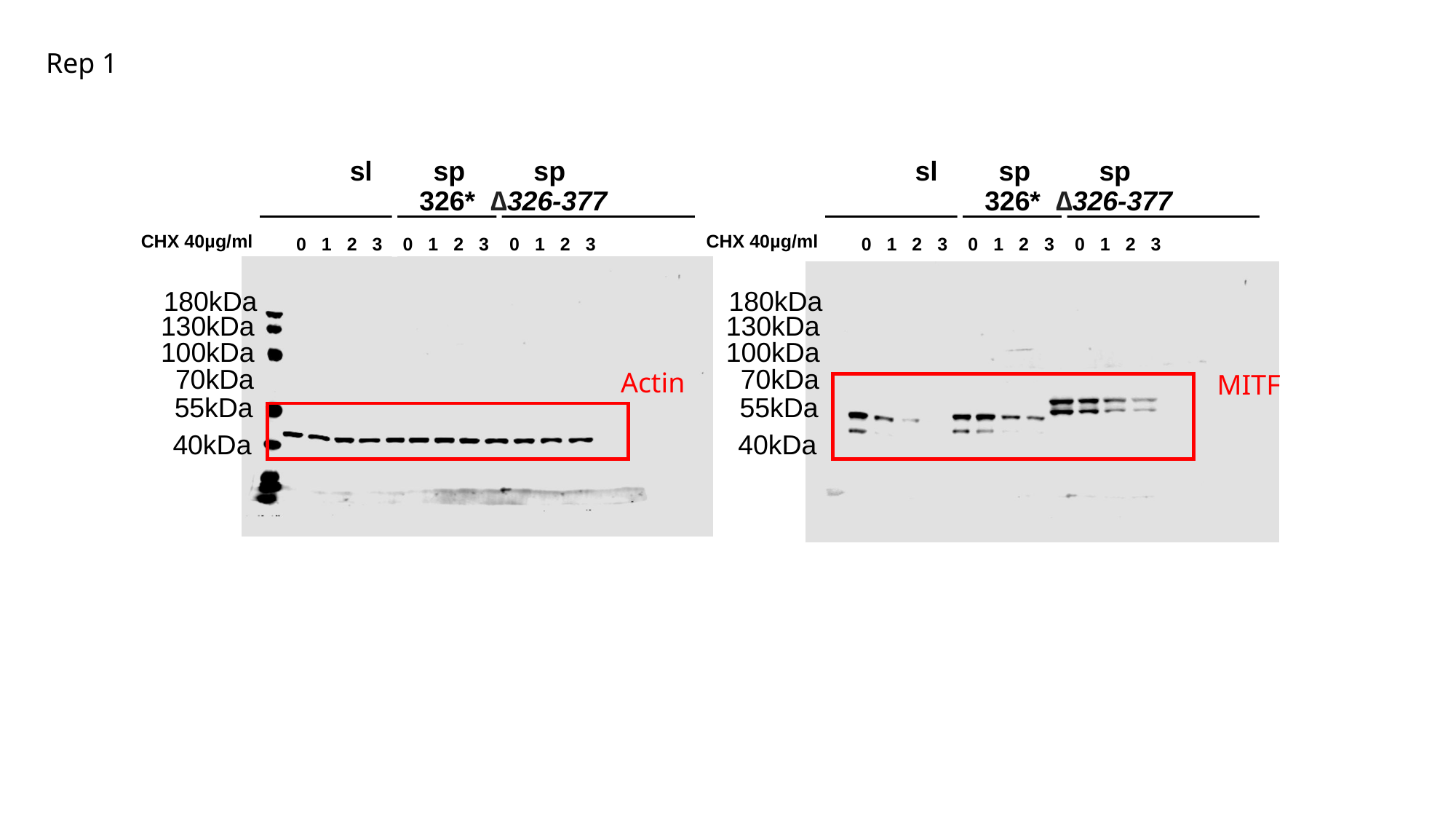

Rep 1
sl sp sp
sl sp sp
326* ∆326-377
326* ∆326-377
CHX 40µg/ml
CHX 40µg/ml
 0 1 2 3
 0 1 2 3
 0 1 2 3
 0 1 2 3
 0 1 2 3
 0 1 2 3
180kDa
180kDa
130kDa
130kDa
100kDa
100kDa
70kDa
70kDa
Actin
MITF
55kDa
55kDa
40kDa
40kDa

## Slide 3
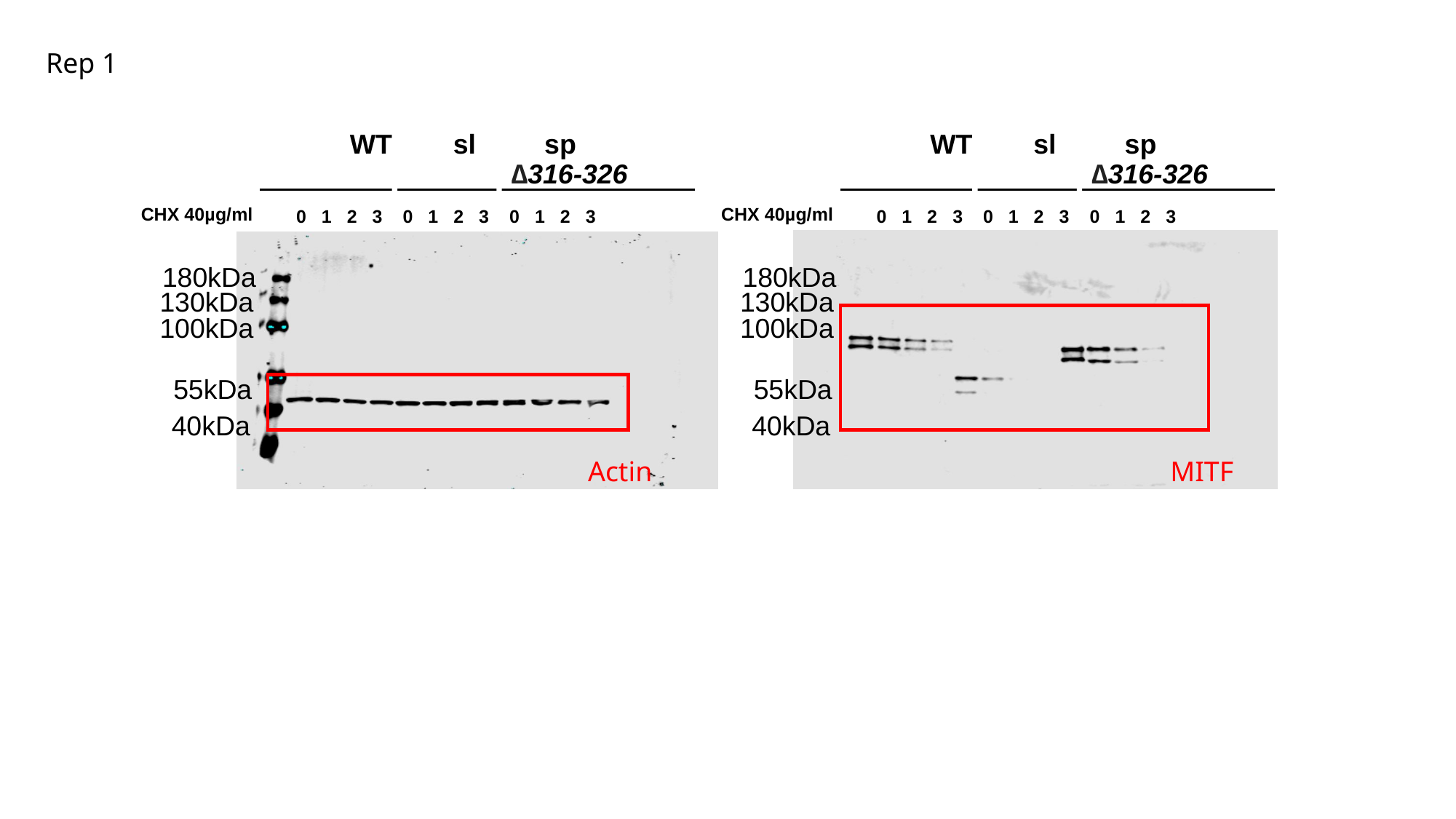

Rep 1
WT sl sp
WT sl sp
 ∆316-326
 ∆316-326
CHX 40µg/ml
CHX 40µg/ml
 0 1 2 3
 0 1 2 3
 0 1 2 3
 0 1 2 3
 0 1 2 3
 0 1 2 3
180kDa
180kDa
130kDa
130kDa
100kDa
100kDa
55kDa
55kDa
40kDa
40kDa
MITF
Actin

## Slide 4
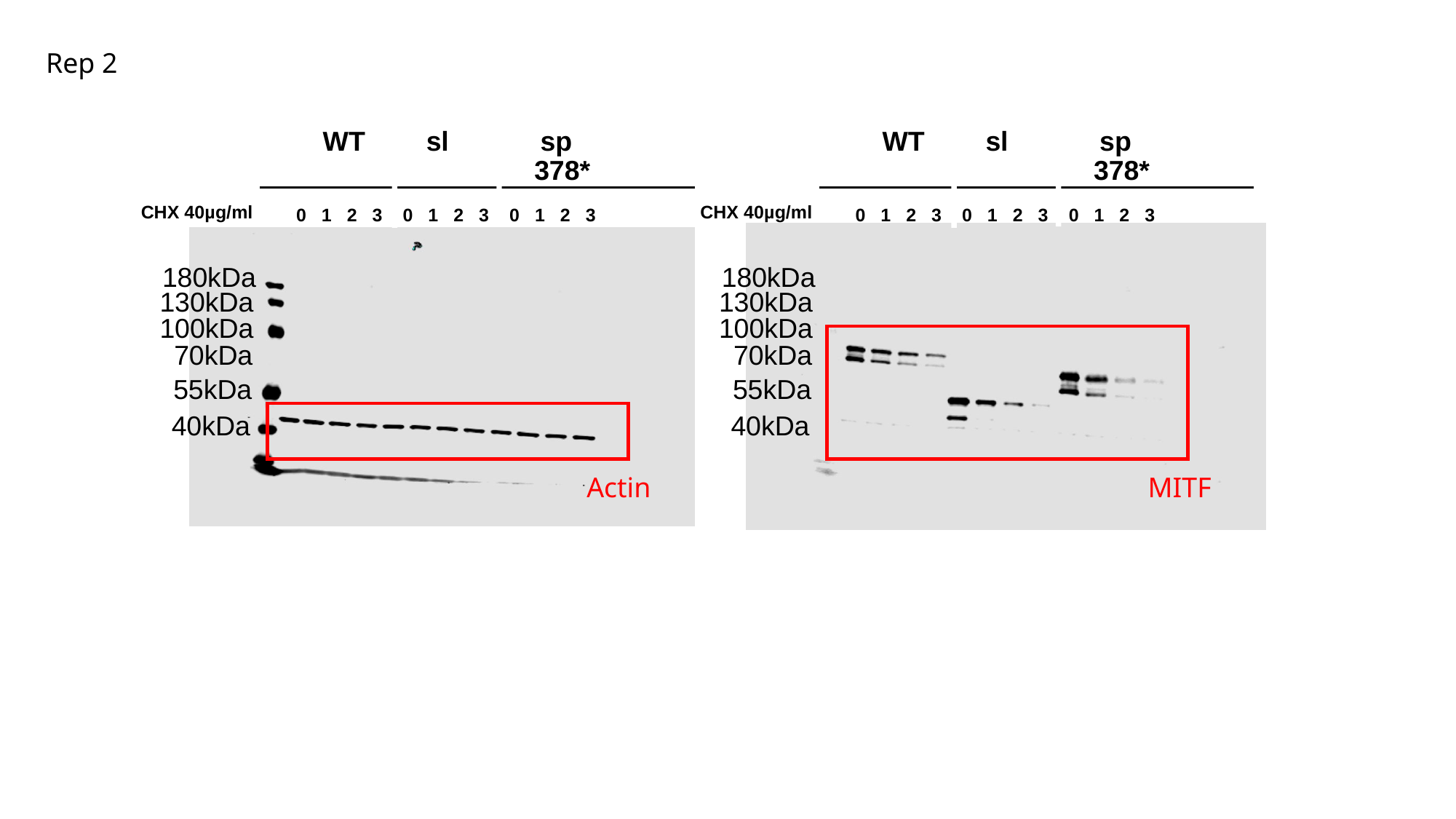

Rep 2
WT sl sp
WT sl sp
378*
378*
CHX 40µg/ml
CHX 40µg/ml
 0 1 2 3
 0 1 2 3
 0 1 2 3
 0 1 2 3
 0 1 2 3
 0 1 2 3
180kDa
180kDa
130kDa
130kDa
100kDa
100kDa
70kDa
70kDa
55kDa
55kDa
40kDa
40kDa
Actin
MITF

## Slide 5
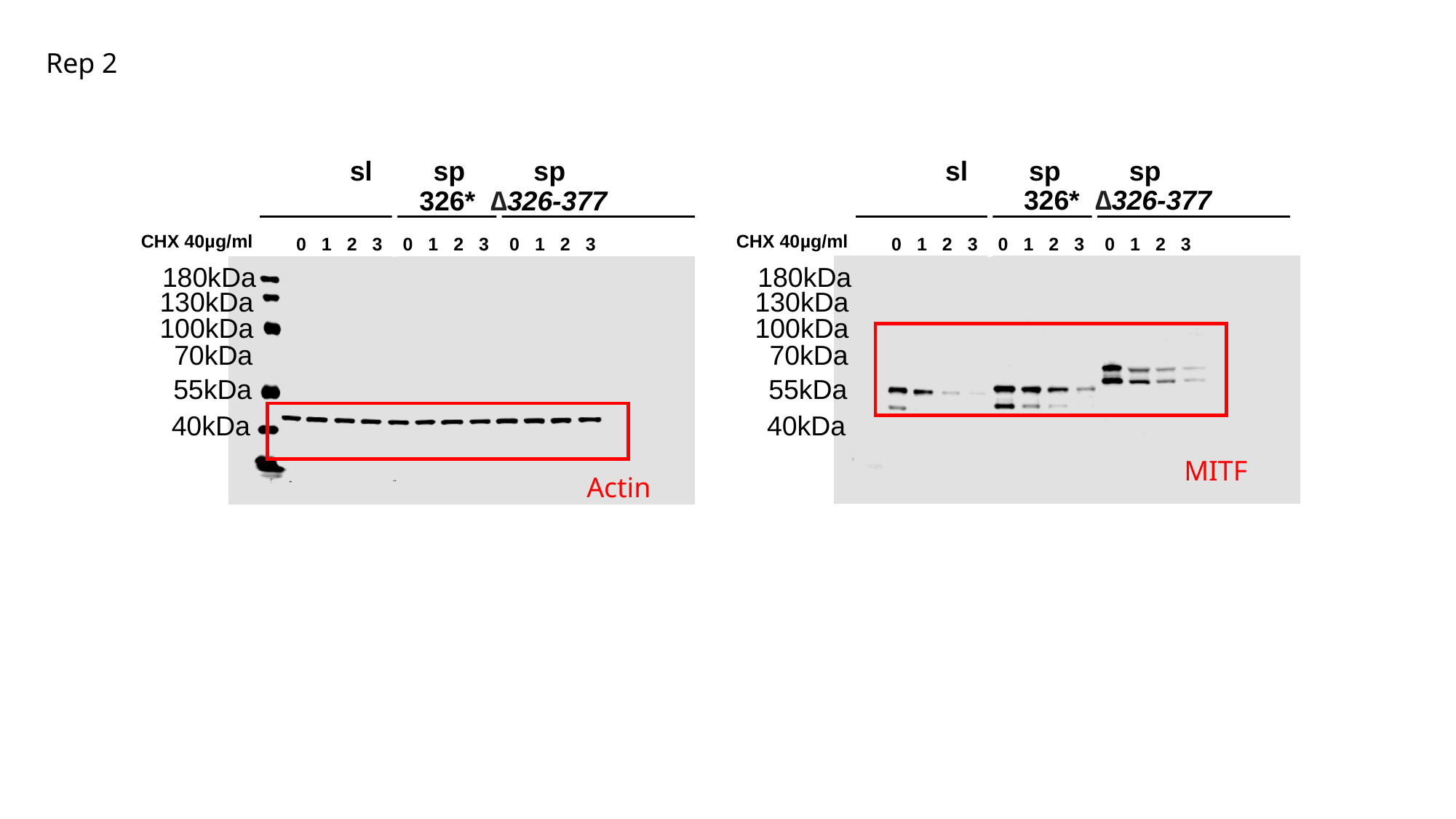

Rep 2
sl sp sp
sl sp sp
326* ∆326-377
326* ∆326-377
CHX 40µg/ml
CHX 40µg/ml
 0 1 2 3
 0 1 2 3
 0 1 2 3
 0 1 2 3
 0 1 2 3
 0 1 2 3
180kDa
180kDa
130kDa
130kDa
100kDa
100kDa
70kDa
70kDa
55kDa
55kDa
40kDa
40kDa
MITF
Actin

## Slide 6
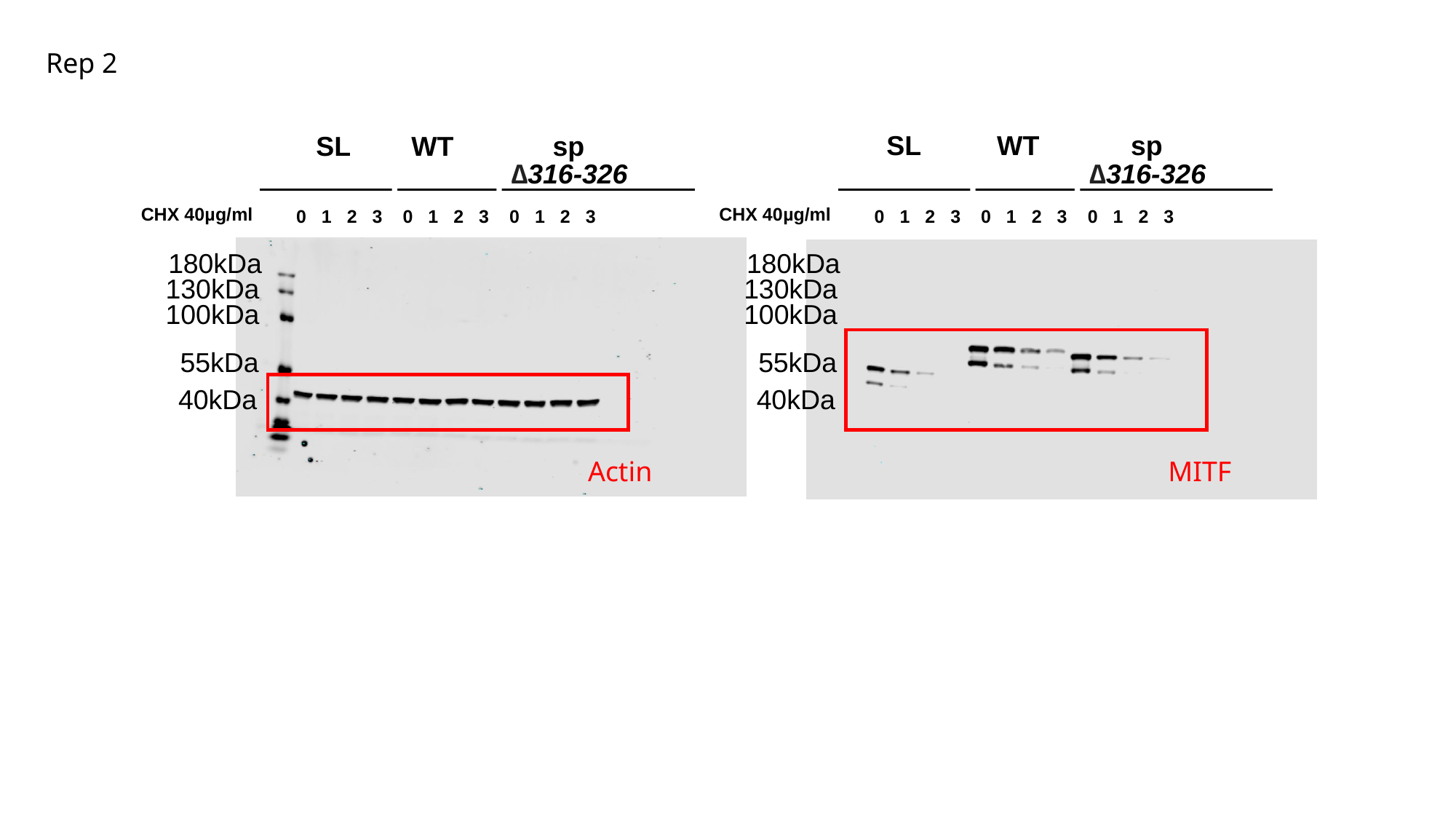

Rep 2
SL WT sp
SL WT sp
 ∆316-326
 ∆316-326
CHX 40µg/ml
CHX 40µg/ml
 0 1 2 3
 0 1 2 3
 0 1 2 3
 0 1 2 3
 0 1 2 3
 0 1 2 3
180kDa
180kDa
130kDa
130kDa
100kDa
100kDa
55kDa
55kDa
40kDa
40kDa
MITF
Actin

## Slide 7
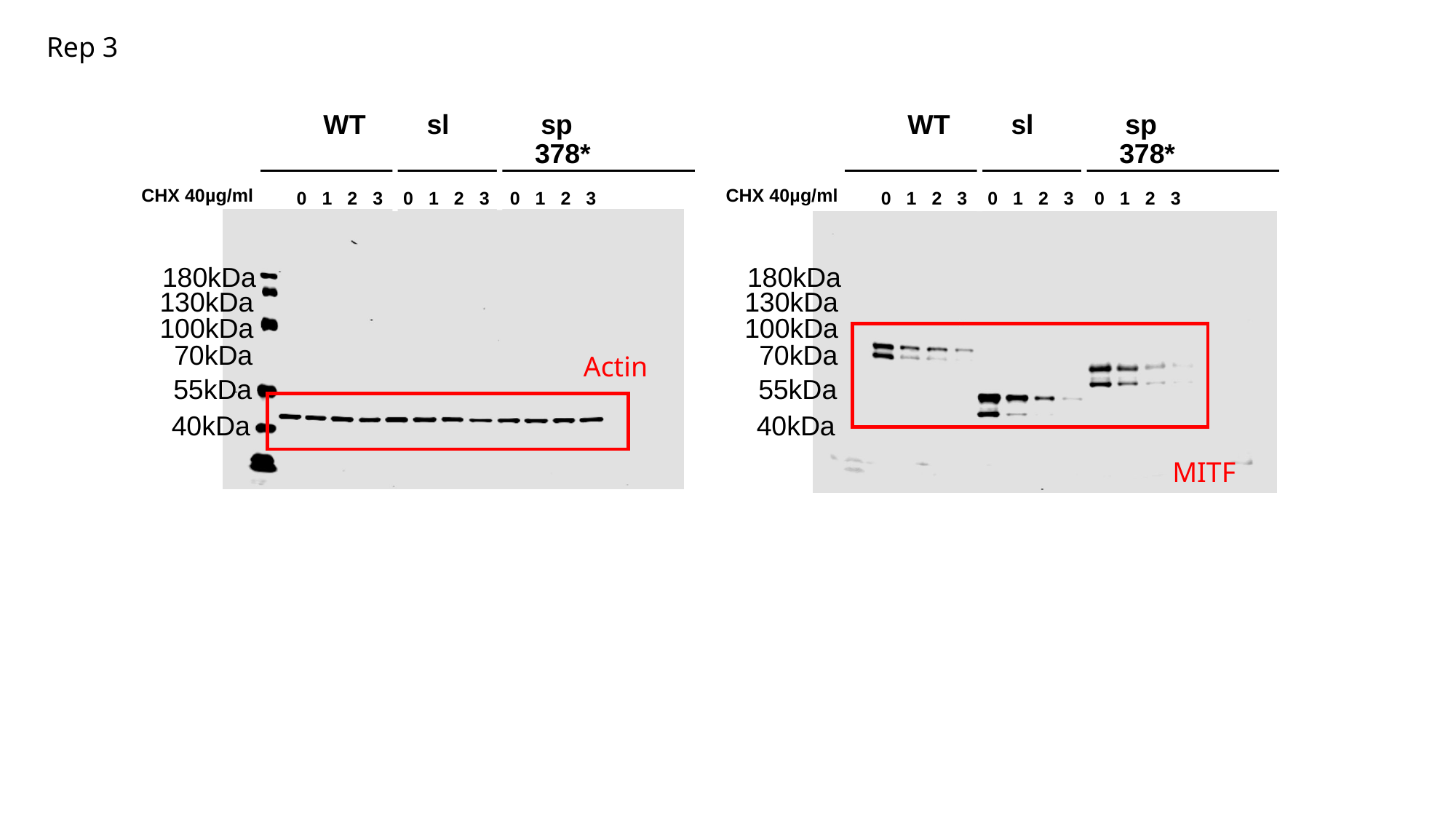

Rep 3
WT sl sp
WT sl sp
378*
378*
CHX 40µg/ml
CHX 40µg/ml
 0 1 2 3
 0 1 2 3
 0 1 2 3
 0 1 2 3
 0 1 2 3
 0 1 2 3
180kDa
180kDa
130kDa
130kDa
100kDa
100kDa
70kDa
70kDa
Actin
55kDa
55kDa
40kDa
40kDa
MITF

## Slide 8
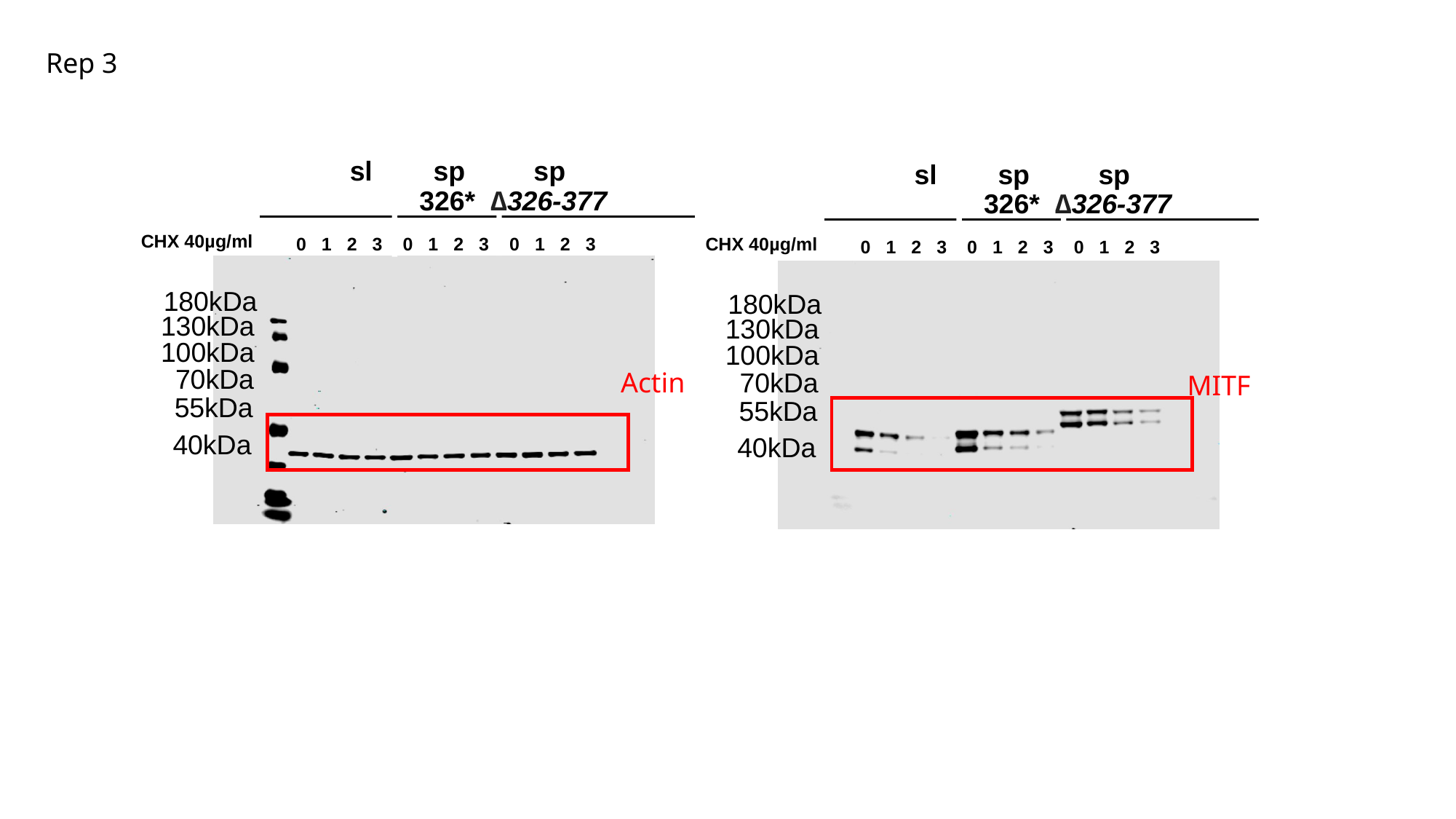

Rep 3
sl sp sp
sl sp sp
326* ∆326-377
326* ∆326-377
CHX 40µg/ml
 0 1 2 3
 0 1 2 3
 0 1 2 3
CHX 40µg/ml
 0 1 2 3
 0 1 2 3
 0 1 2 3
180kDa
180kDa
130kDa
130kDa
100kDa
100kDa
70kDa
70kDa
Actin
MITF
55kDa
55kDa
40kDa
40kDa

## Slide 9
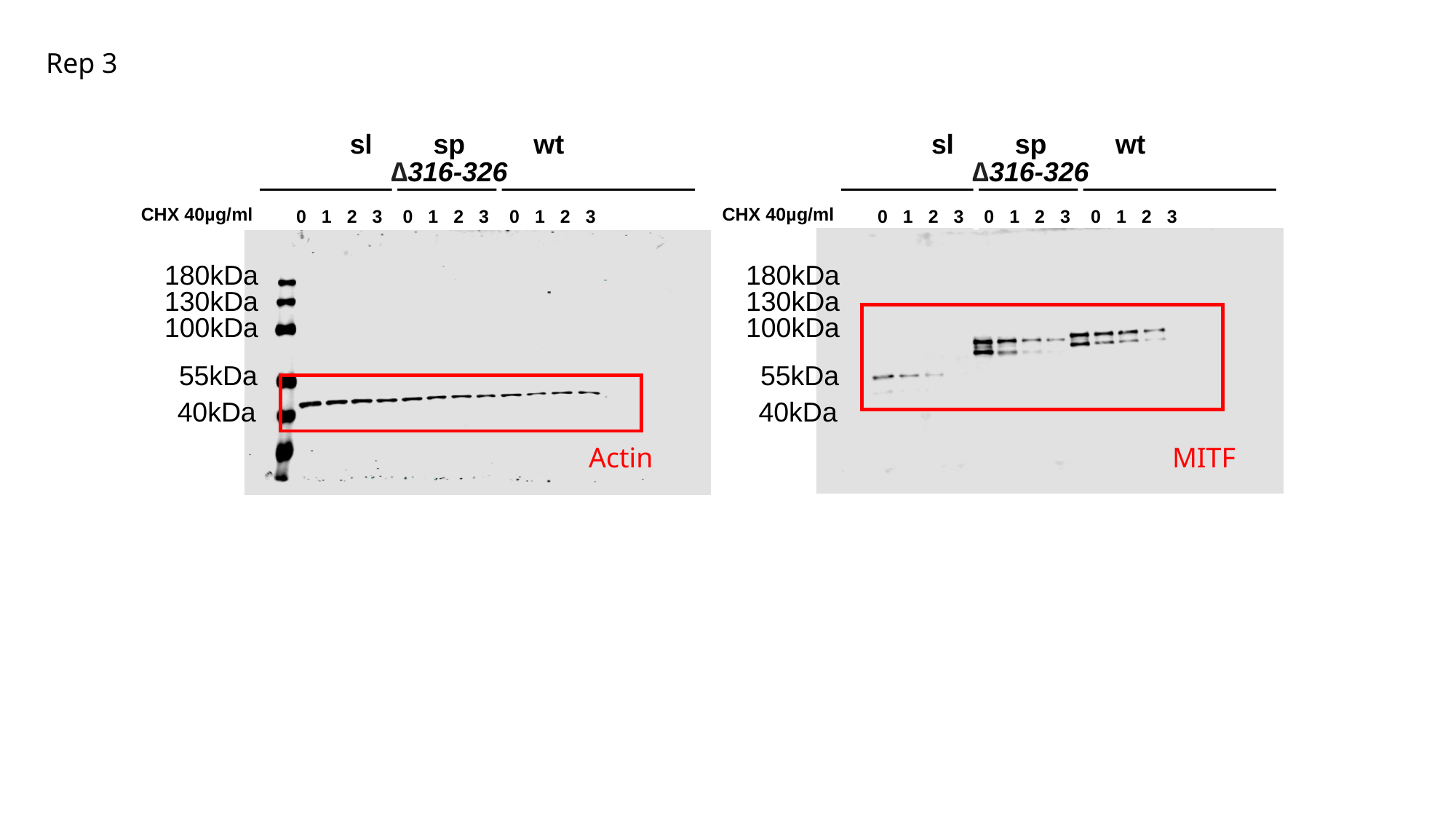

Rep 3
sl sp wt
sl sp wt
 ∆316-326
 ∆316-326
CHX 40µg/ml
CHX 40µg/ml
 0 1 2 3
 0 1 2 3
 0 1 2 3
 0 1 2 3
 0 1 2 3
 0 1 2 3
180kDa
180kDa
130kDa
130kDa
100kDa
100kDa
55kDa
55kDa
40kDa
40kDa
Actin
MITF
