# Supplementary material for: Novel mechanisms of MITF regulation identified in a mouse suppressor screen
Source: EMBO Rep. 2024 Aug 21;25(10):4252–80. doi: 10.1038/s44319-024-00225-3 (PMC11467436; doi:10.1038/s44319-024-00225-3)
Supplement: Supplementary file 6 — Source data Fig. 3 [file 44319_2024_225_MOESM6_ESM.zip › 3B/Figure 3B.pptx]

## Slide 1
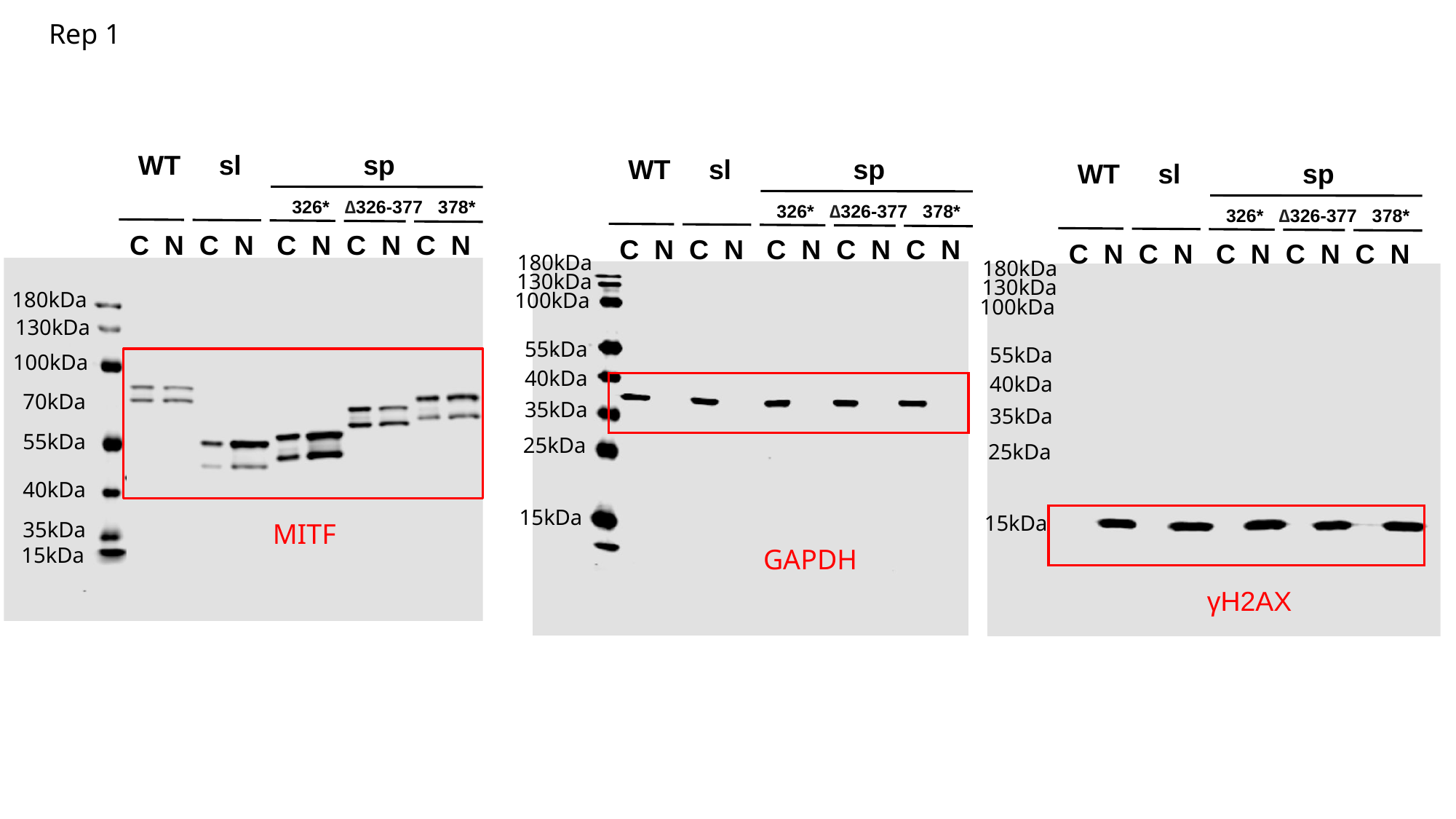

Rep 1
 WT sl sp
 WT sl sp
 WT sl sp
326* ∆326-377 378*
326* ∆326-377 378*
326* ∆326-377 378*
 C N C N C N C N C N
 C N C N C N C N C N
 C N C N C N C N C N
180kDa
180kDa
130kDa
130kDa
180kDa
100kDa
100kDa
130kDa
55kDa
55kDa
100kDa
40kDa
40kDa
70kDa
35kDa
35kDa
55kDa
25kDa
25kDa
40kDa
15kDa
15kDa
35kDa
MITF
15kDa
GAPDH
γH2AX

## Slide 2
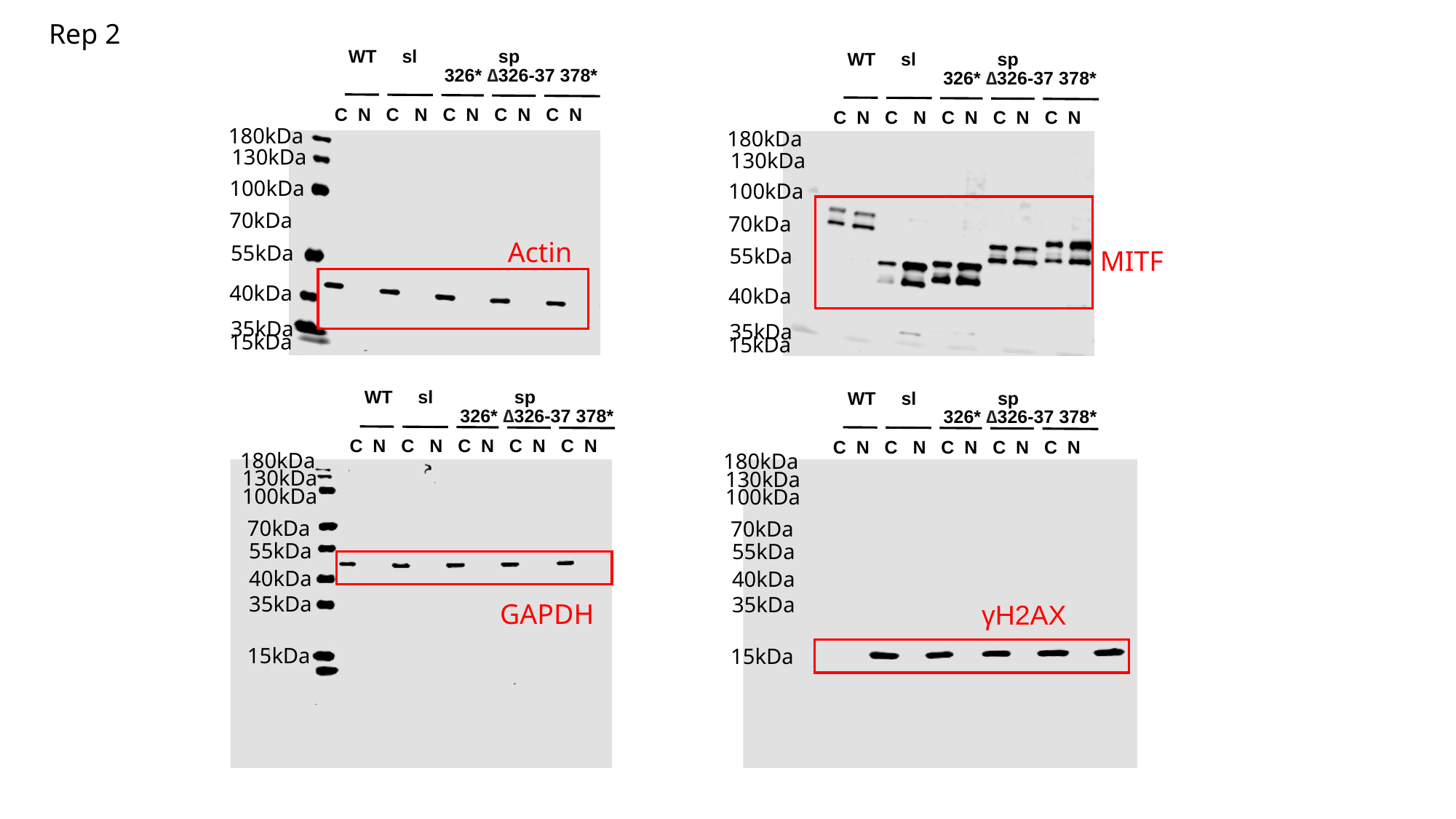

Rep 2
 WT sl sp
 WT sl sp
326* ∆326-37 378*
326* ∆326-37 378*
 C N C N C N C N C N
 C N C N C N C N C N
180kDa
180kDa
130kDa
130kDa
100kDa
100kDa
70kDa
70kDa
Actin
55kDa
55kDa
MITF
40kDa
40kDa
35kDa
35kDa
15kDa
15kDa
 WT sl sp
 WT sl sp
326* ∆326-37 378*
326* ∆326-37 378*
 C N C N C N C N C N
 C N C N C N C N C N
180kDa
180kDa
130kDa
130kDa
100kDa
100kDa
70kDa
70kDa
55kDa
55kDa
40kDa
40kDa
35kDa
35kDa
GAPDH
γH2AX
15kDa
15kDa

## Slide 3
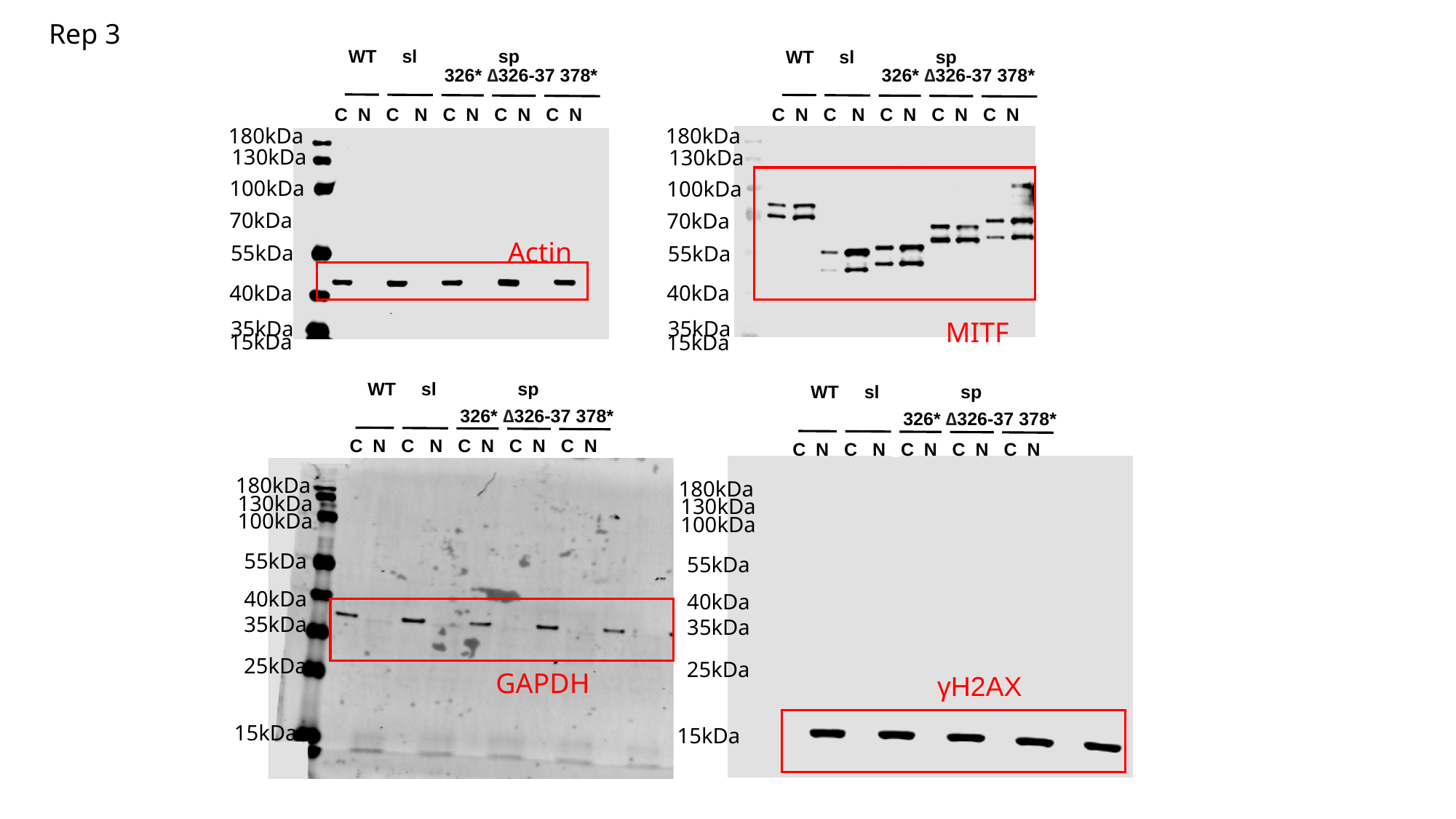

Rep 3
 WT sl sp
 WT sl sp
326* ∆326-37 378*
326* ∆326-37 378*
 C N C N C N C N C N
 C N C N C N C N C N
180kDa
180kDa
130kDa
130kDa
100kDa
100kDa
70kDa
70kDa
Actin
55kDa
55kDa
40kDa
40kDa
35kDa
MITF
35kDa
15kDa
15kDa
 WT sl sp
 WT sl sp
326* ∆326-37 378*
326* ∆326-37 378*
 C N C N C N C N C N
 C N C N C N C N C N
180kDa
180kDa
130kDa
130kDa
100kDa
100kDa
55kDa
55kDa
40kDa
40kDa
35kDa
35kDa
25kDa
25kDa
GAPDH
γH2AX
15kDa
15kDa
