# Supplementary material for: Novel mechanisms of MITF regulation identified in a mouse suppressor screen
Source: EMBO Rep. 2024 Aug 21;25(10):4252–80. doi: 10.1038/s44319-024-00225-3 (PMC11467436; doi:10.1038/s44319-024-00225-3)
Supplement: Supplementary file 6 — Source data Fig. 3 [file 44319_2024_225_MOESM6_ESM.zip › 3D/Figure 3D.pptx]

## Slide 1
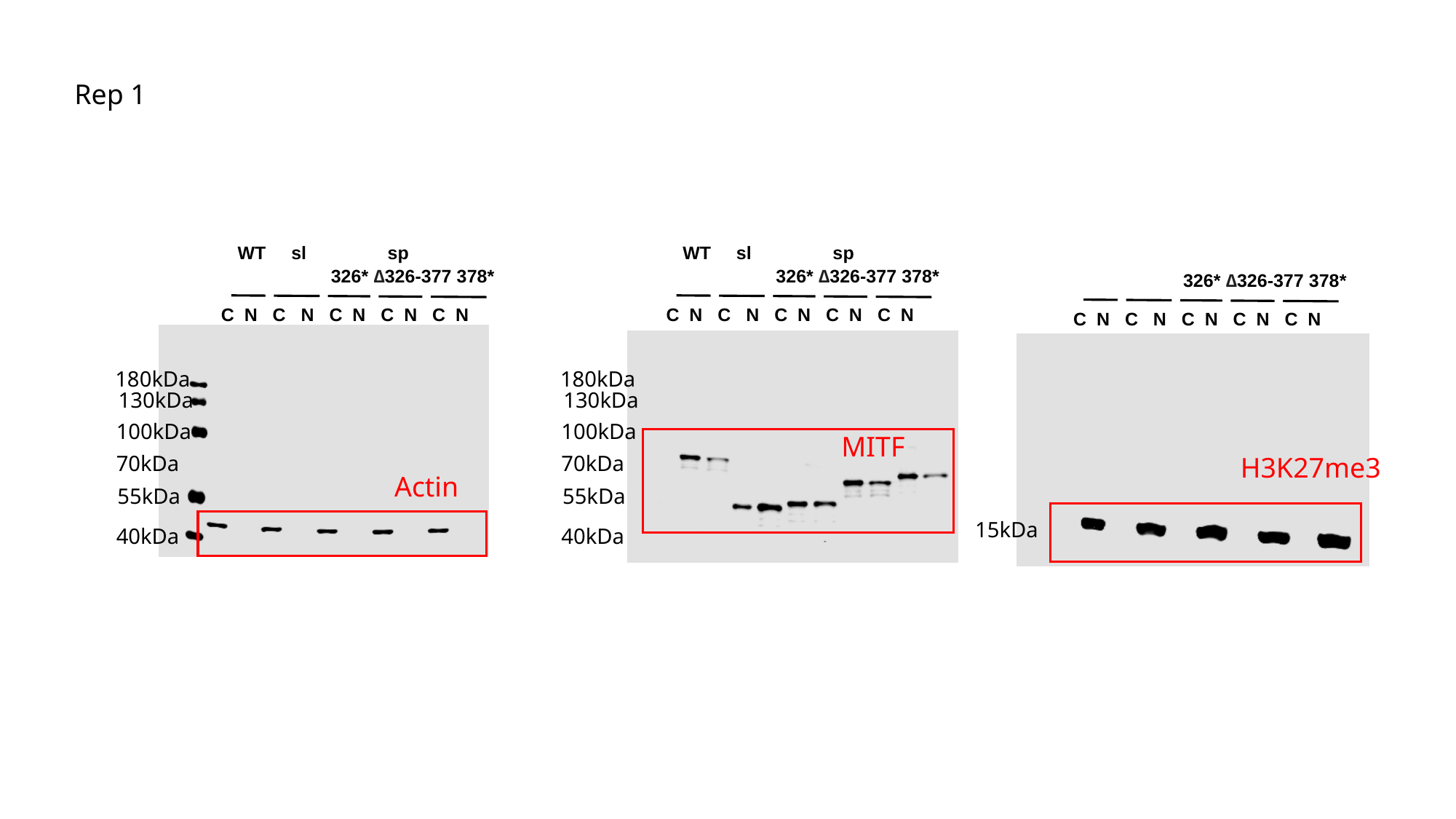

Rep 1
 WT sl sp
 WT sl sp
326* ∆326-377 378*
326* ∆326-377 378*
326* ∆326-377 378*
 C N C N C N C N C N
 C N C N C N C N C N
 C N C N C N C N C N
180kDa
180kDa
130kDa
130kDa
100kDa
100kDa
MITF
70kDa
70kDa
H3K27me3
Actin
55kDa
55kDa
15kDa
40kDa
40kDa

## Slide 2
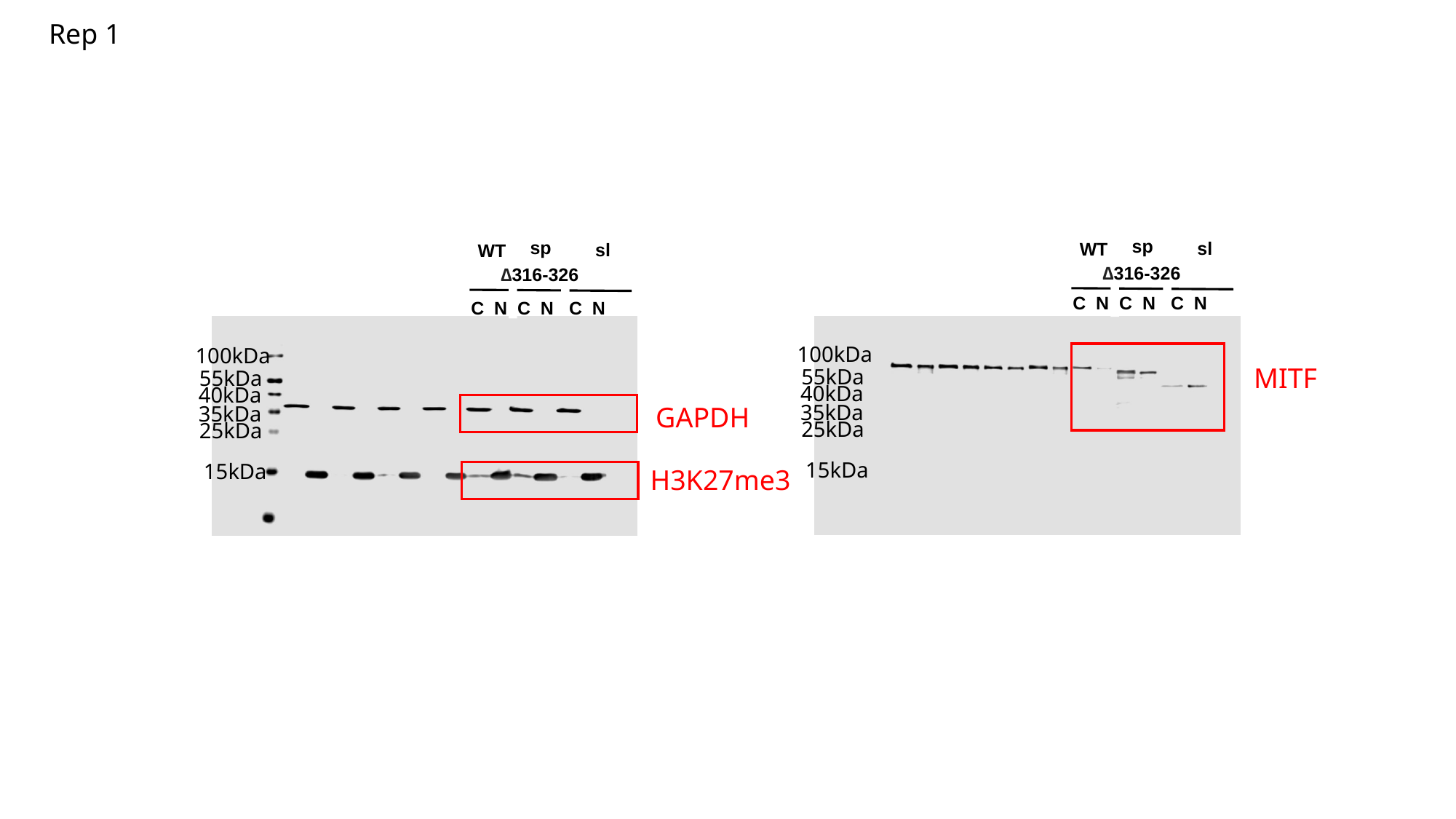

Rep 1
sp
sp
sl
 WT
sl
 WT
 ∆316-326
 ∆316-326
 C N C N C N
 C N C N C N
100kDa
100kDa
MITF
55kDa
55kDa
40kDa
40kDa
35kDa
GAPDH
35kDa
25kDa
25kDa
15kDa
15kDa
H3K27me3

## Slide 3
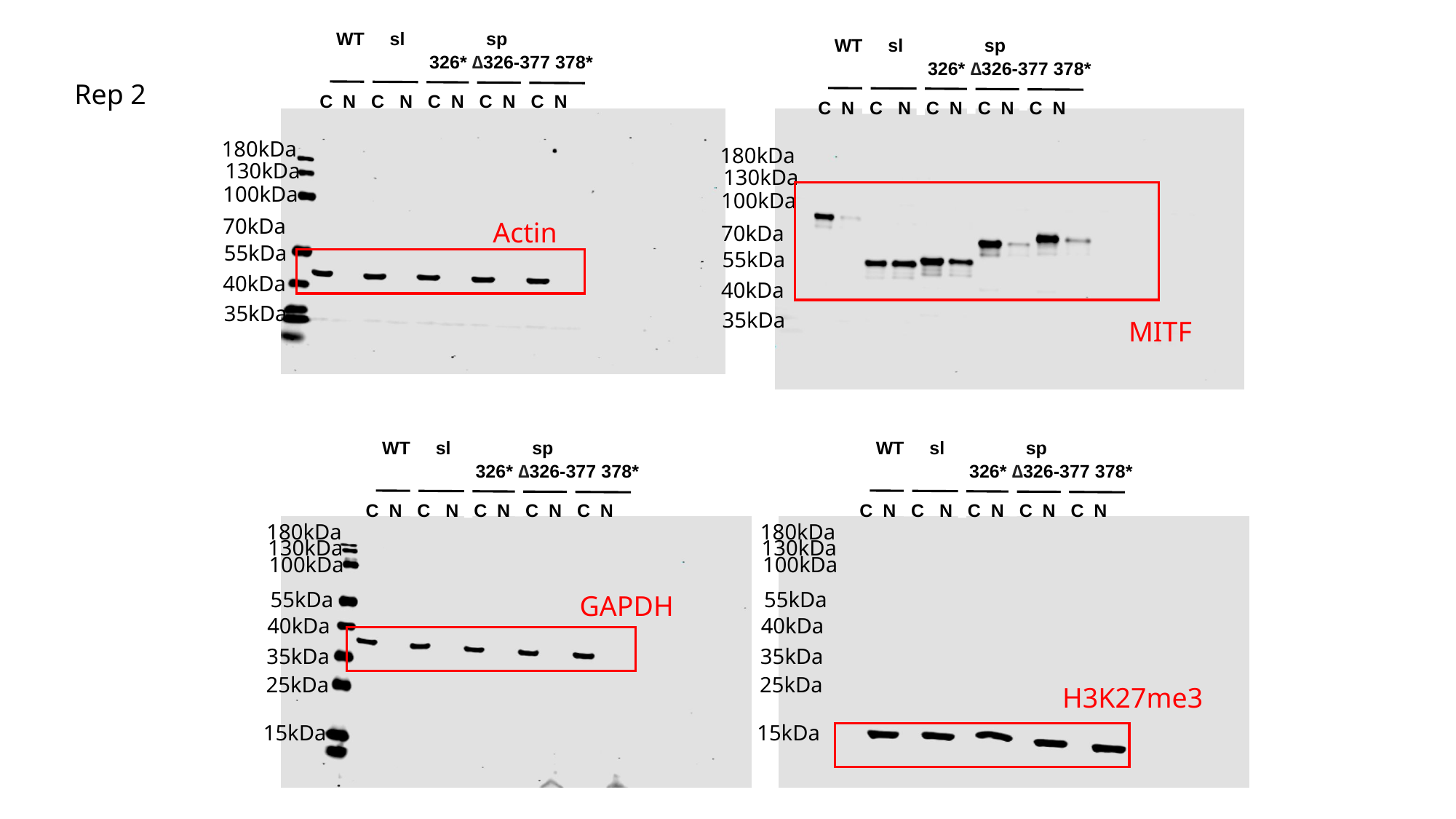

WT sl sp
 WT sl sp
326* ∆326-377 378*
326* ∆326-377 378*
Rep 2
 C N C N C N C N C N
 C N C N C N C N C N
180kDa
180kDa
130kDa
130kDa
100kDa
100kDa
70kDa
Actin
70kDa
55kDa
55kDa
40kDa
40kDa
35kDa
35kDa
MITF
 WT sl sp
 WT sl sp
326* ∆326-377 378*
326* ∆326-377 378*
 C N C N C N C N C N
 C N C N C N C N C N
180kDa
180kDa
130kDa
130kDa
100kDa
100kDa
55kDa
55kDa
GAPDH
40kDa
40kDa
35kDa
35kDa
25kDa
25kDa
H3K27me3
15kDa
15kDa

## Slide 4
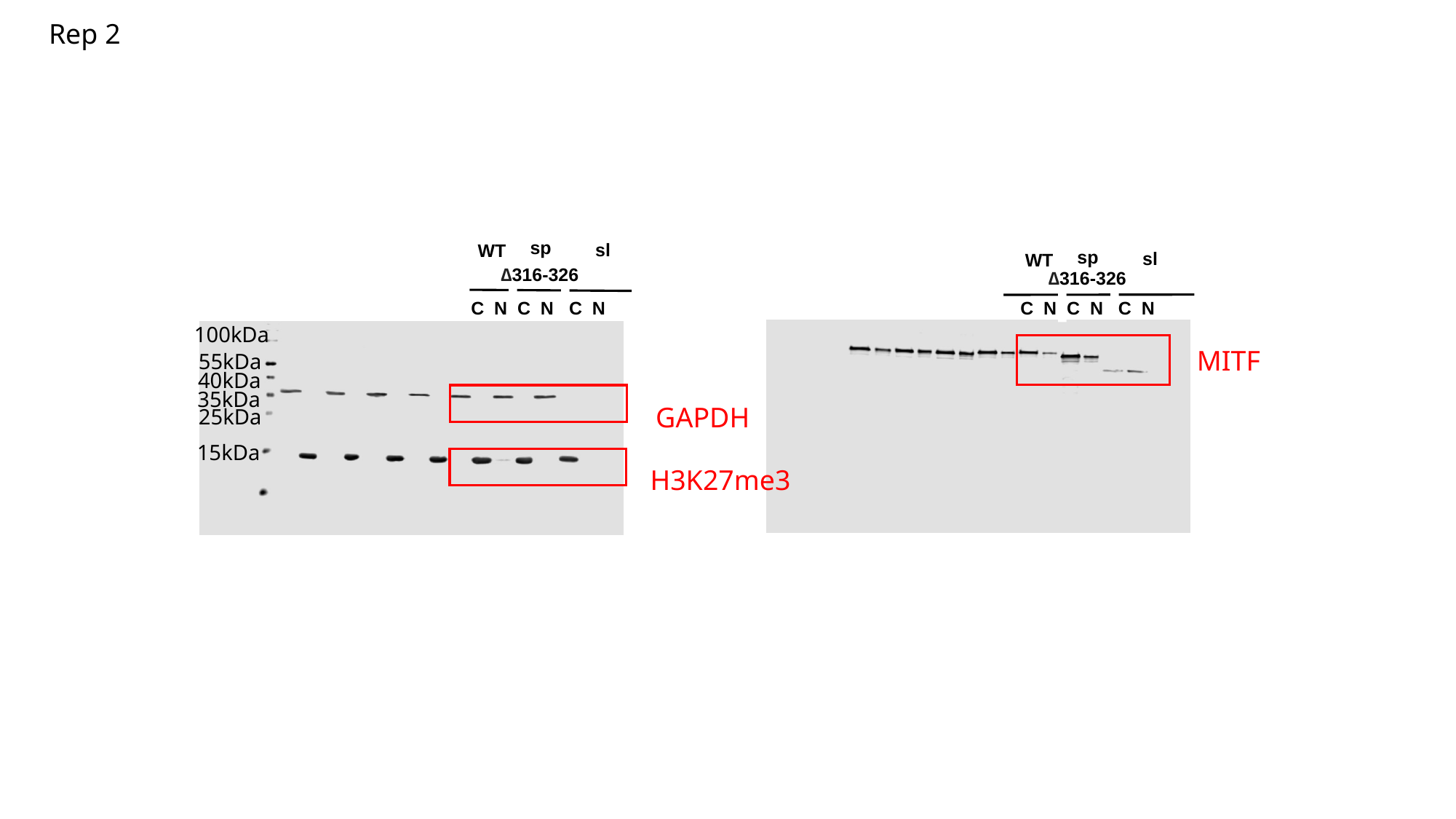

Rep 2
sp
sl
 WT
sp
sl
 WT
 ∆316-326
 ∆316-326
 C N C N C N
 C N C N C N
100kDa
MITF
55kDa
40kDa
35kDa
GAPDH
25kDa
15kDa
H3K27me3

## Slide 5
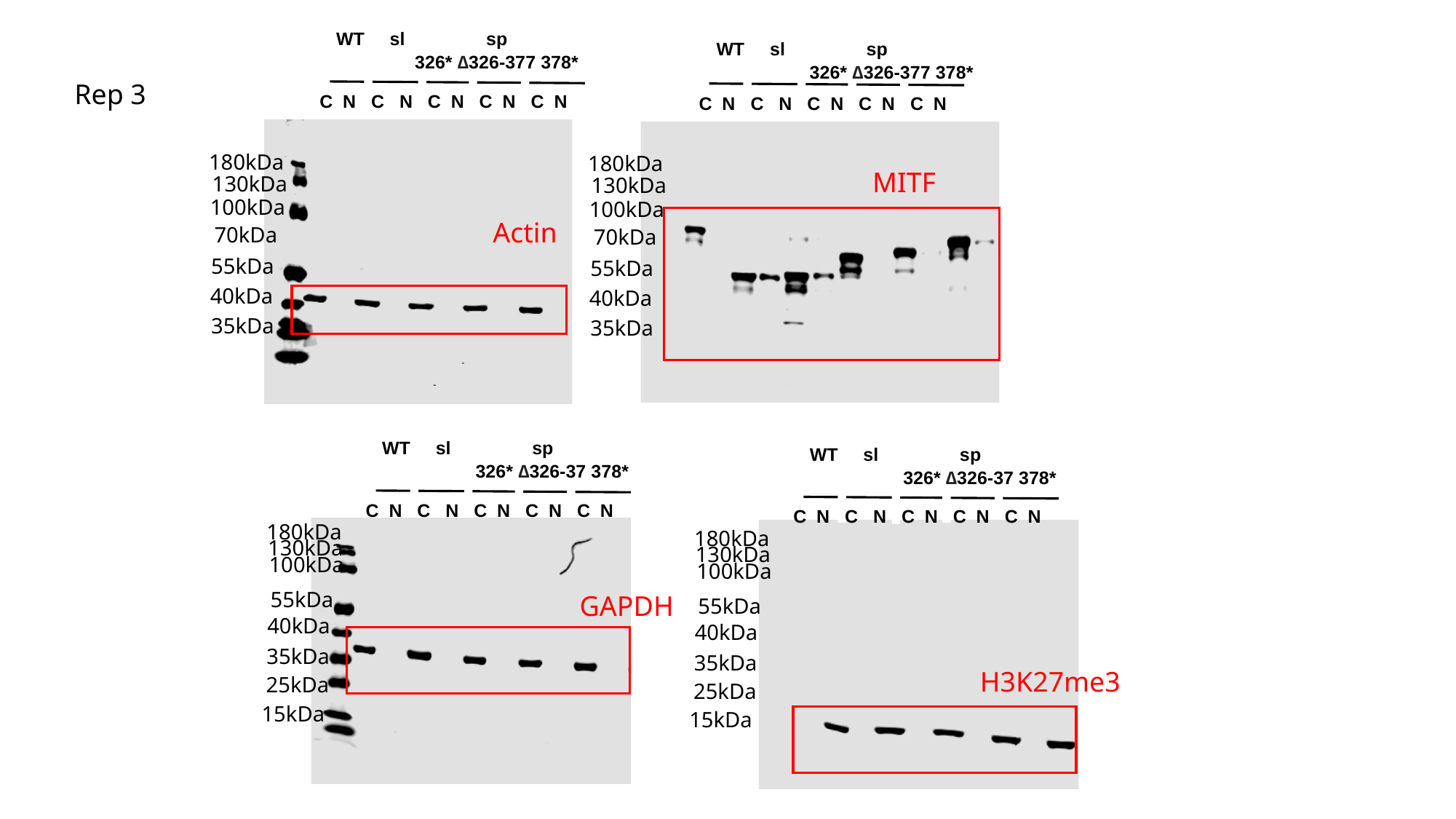

WT sl sp
 WT sl sp
326* ∆326-377 378*
326* ∆326-377 378*
Rep 3
 C N C N C N C N C N
 C N C N C N C N C N
180kDa
180kDa
MITF
130kDa
130kDa
100kDa
100kDa
Actin
70kDa
70kDa
55kDa
55kDa
40kDa
40kDa
35kDa
35kDa
 WT sl sp
 WT sl sp
326* ∆326-37 378*
326* ∆326-37 378*
 C N C N C N C N C N
 C N C N C N C N C N
180kDa
180kDa
130kDa
130kDa
100kDa
100kDa
55kDa
GAPDH
55kDa
40kDa
40kDa
35kDa
35kDa
H3K27me3
25kDa
25kDa
15kDa
15kDa

## Slide 6
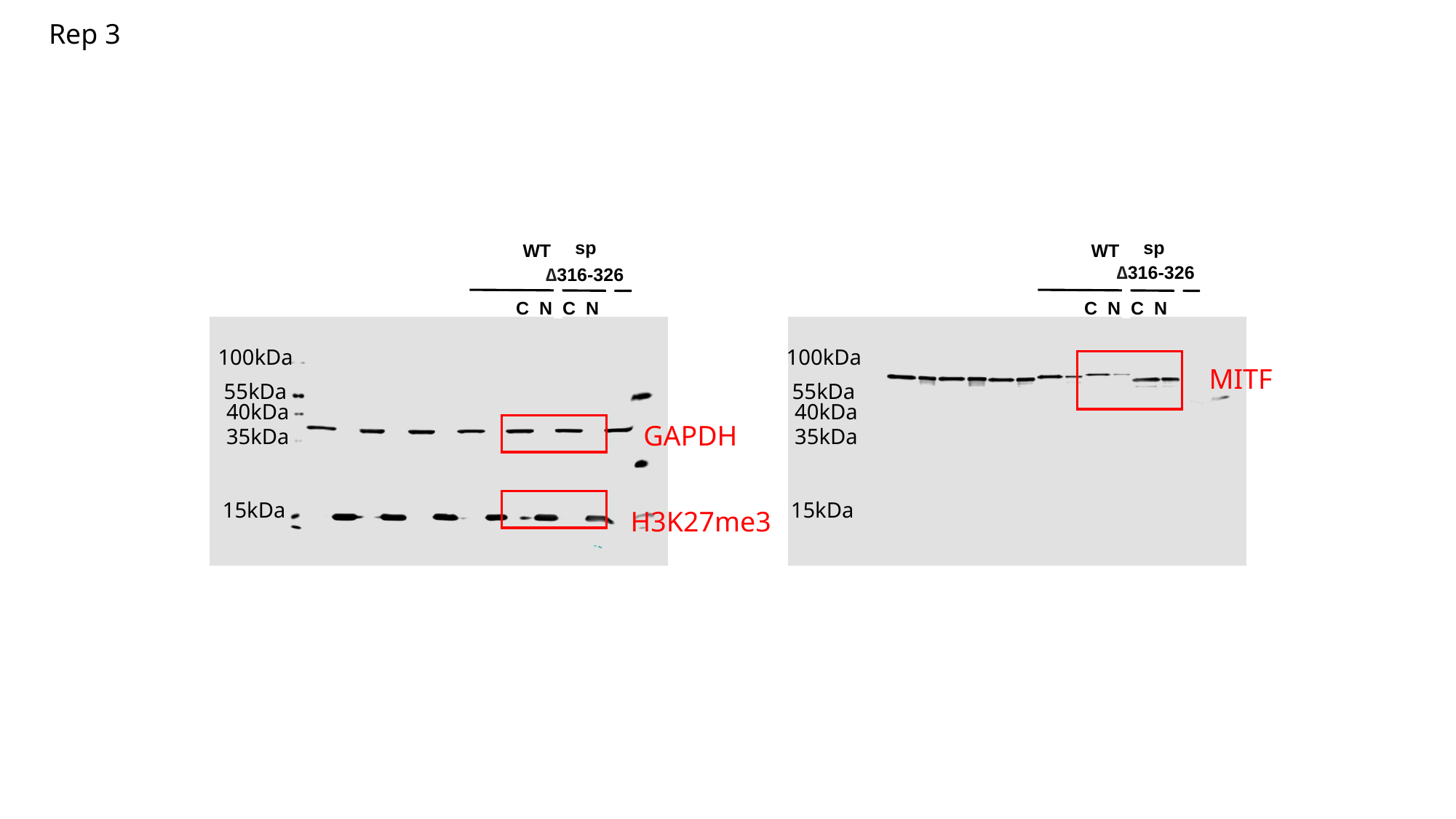

Rep 3
sp
sp
 WT
 WT
 ∆316-326
 ∆316-326
 C N C N
 C N C N
100kDa
100kDa
MITF
55kDa
55kDa
40kDa
40kDa
GAPDH
35kDa
35kDa
15kDa
15kDa
H3K27me3
