# Supplementary material for: Novel mechanisms of MITF regulation identified in a mouse suppressor screen
Source: EMBO Rep. 2024 Aug 21;25(10):4252–80. doi: 10.1038/s44319-024-00225-3 (PMC11467436; doi:10.1038/s44319-024-00225-3)
Supplement: Supplementary file 7 — Source data Fig. 4 [file 44319_2024_225_MOESM7_ESM.zip › 4E/Figure 4E.pptx]

## Slide 1
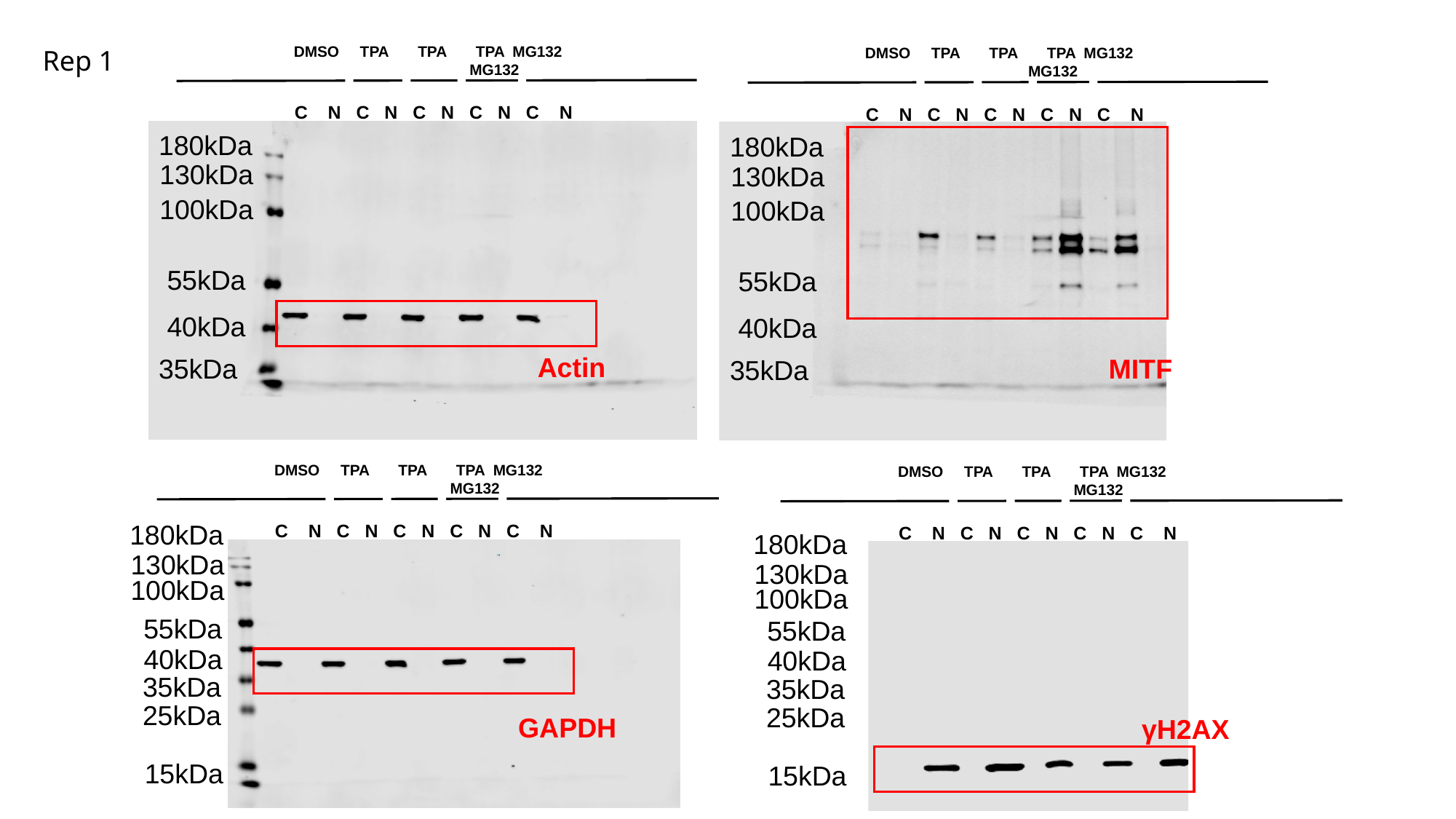

DMSO TPA TPA TPA MG132
 MG132
Rep 1
DMSO TPA TPA TPA MG132
 MG132
C N C N C N C N C N
C N C N C N C N C N
180kDa
180kDa
130kDa
130kDa
100kDa
100kDa
55kDa
55kDa
40kDa
40kDa
Actin
MITF
35kDa
35kDa
DMSO TPA TPA TPA MG132
 MG132
DMSO TPA TPA TPA MG132
 MG132
180kDa
C N C N C N C N C N
C N C N C N C N C N
180kDa
130kDa
130kDa
100kDa
100kDa
55kDa
55kDa
40kDa
40kDa
35kDa
35kDa
25kDa
25kDa
GAPDH
γH2AX
15kDa
15kDa

## Slide 2
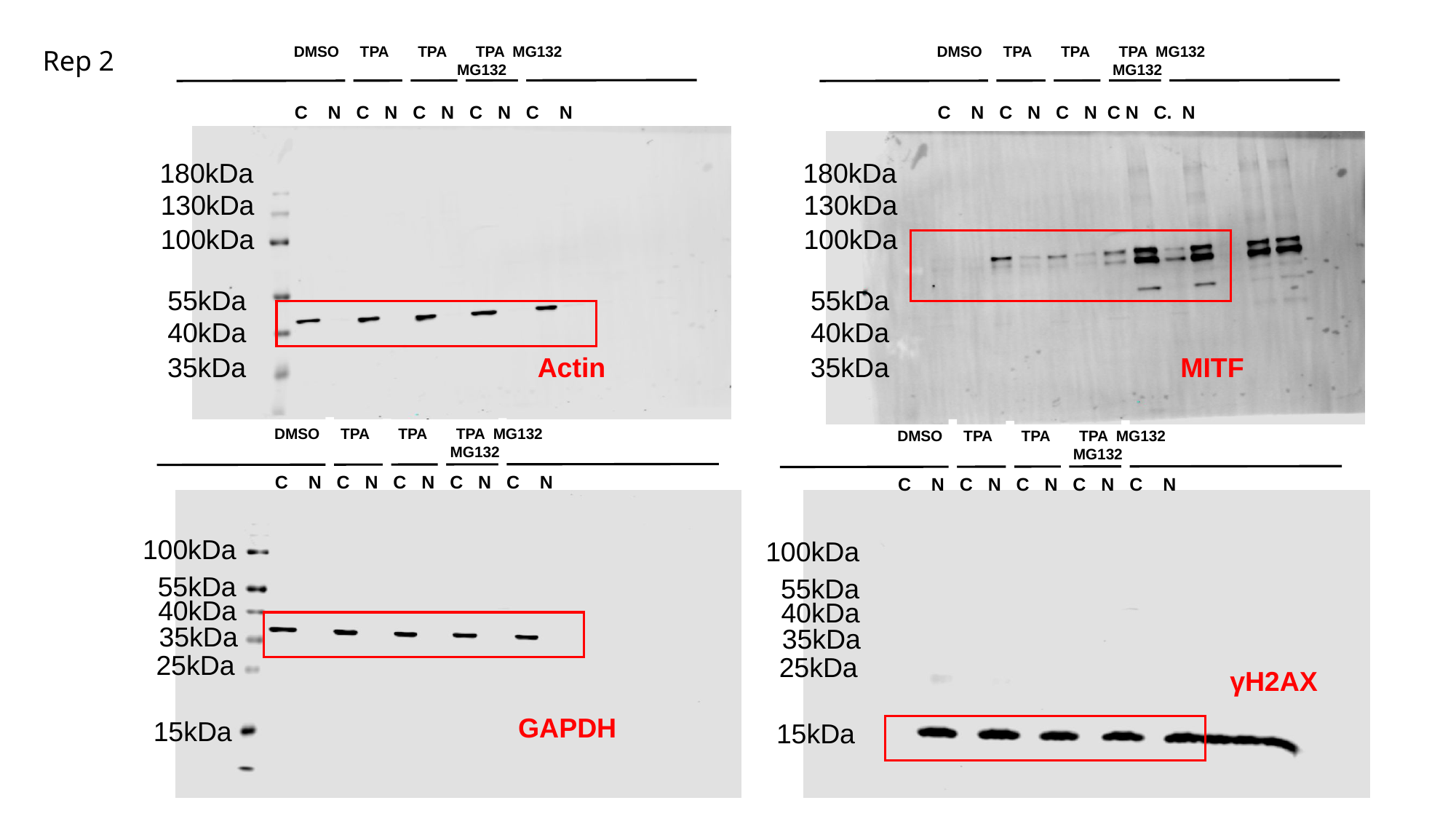

DMSO TPA TPA TPA MG132
 MG132
DMSO TPA TPA TPA MG132
 MG132
Rep 2
C N C N C N C N C N
C N C N C N C N C. N
180kDa
180kDa
130kDa
130kDa
100kDa
100kDa
55kDa
55kDa
40kDa
40kDa
35kDa
Actin
35kDa
MITF
DMSO TPA TPA TPA MG132
 MG132
DMSO TPA TPA TPA MG132
 MG132
C N C N C N C N C N
C N C N C N C N C N
100kDa
100kDa
55kDa
55kDa
40kDa
40kDa
35kDa
35kDa
25kDa
25kDa
γH2AX
GAPDH
15kDa
15kDa

## Slide 3
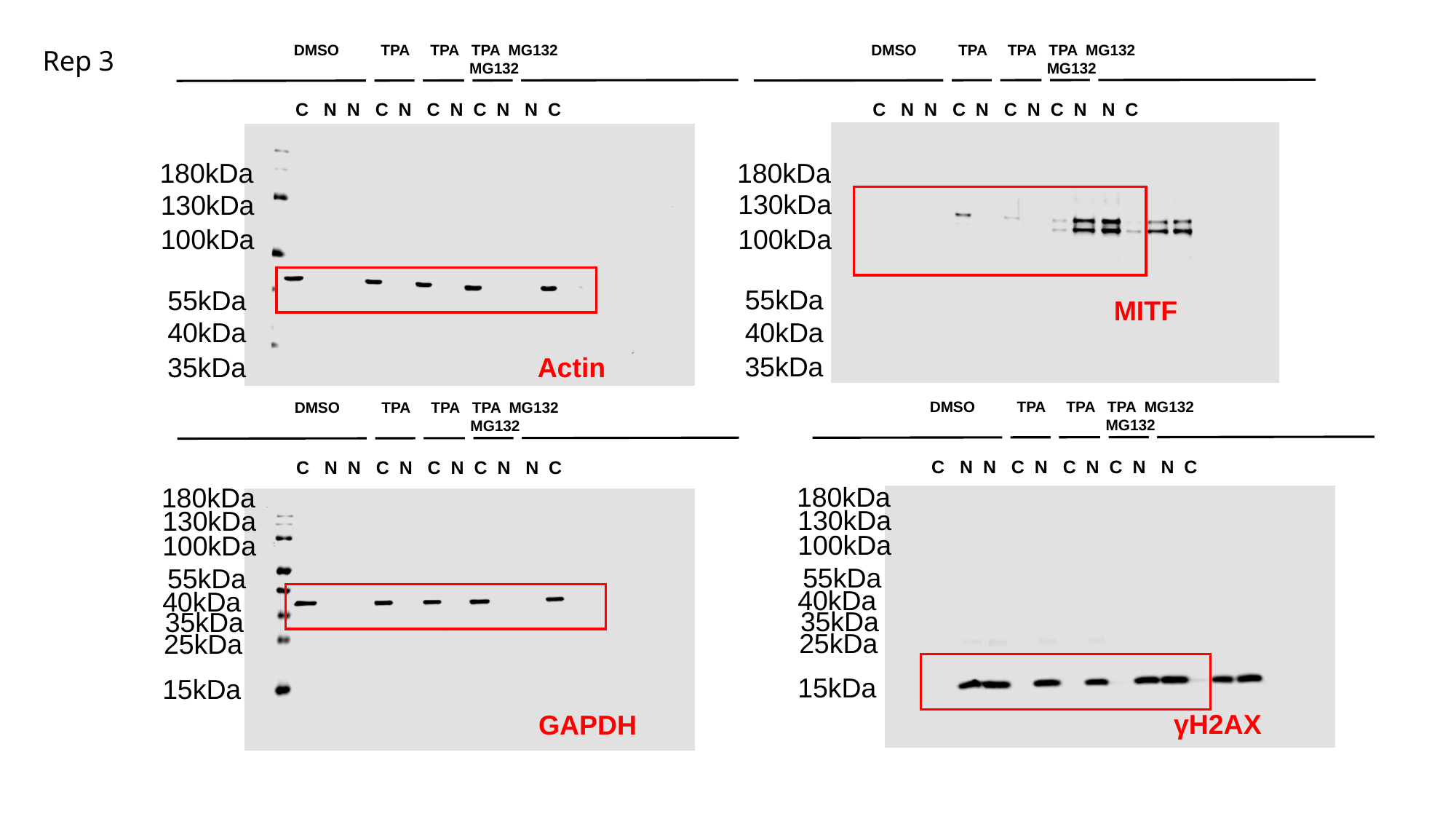

DMSO TPA TPA TPA MG132
 MG132
DMSO TPA TPA TPA MG132
 MG132
Rep 3
C N N C N C N C N N C
C N N C N C N C N N C
180kDa
180kDa
130kDa
130kDa
100kDa
100kDa
55kDa
55kDa
MITF
40kDa
40kDa
35kDa
35kDa
Actin
DMSO TPA TPA TPA MG132
 MG132
DMSO TPA TPA TPA MG132
 MG132
C N N C N C N C N N C
C N N C N C N C N N C
180kDa
180kDa
130kDa
130kDa
100kDa
100kDa
55kDa
55kDa
40kDa
40kDa
35kDa
35kDa
25kDa
25kDa
15kDa
15kDa
γH2AX
GAPDH
