# Supplementary material for: Novel mechanisms of MITF regulation identified in a mouse suppressor screen
Source: EMBO Rep. 2024 Aug 21;25(10):4252–80. doi: 10.1038/s44319-024-00225-3 (PMC11467436; doi:10.1038/s44319-024-00225-3)
Supplement: Supplementary file 7 — Source data Fig. 4 [file 44319_2024_225_MOESM7_ESM.zip › 4F/Figure 4F.pptx]

## Slide 1
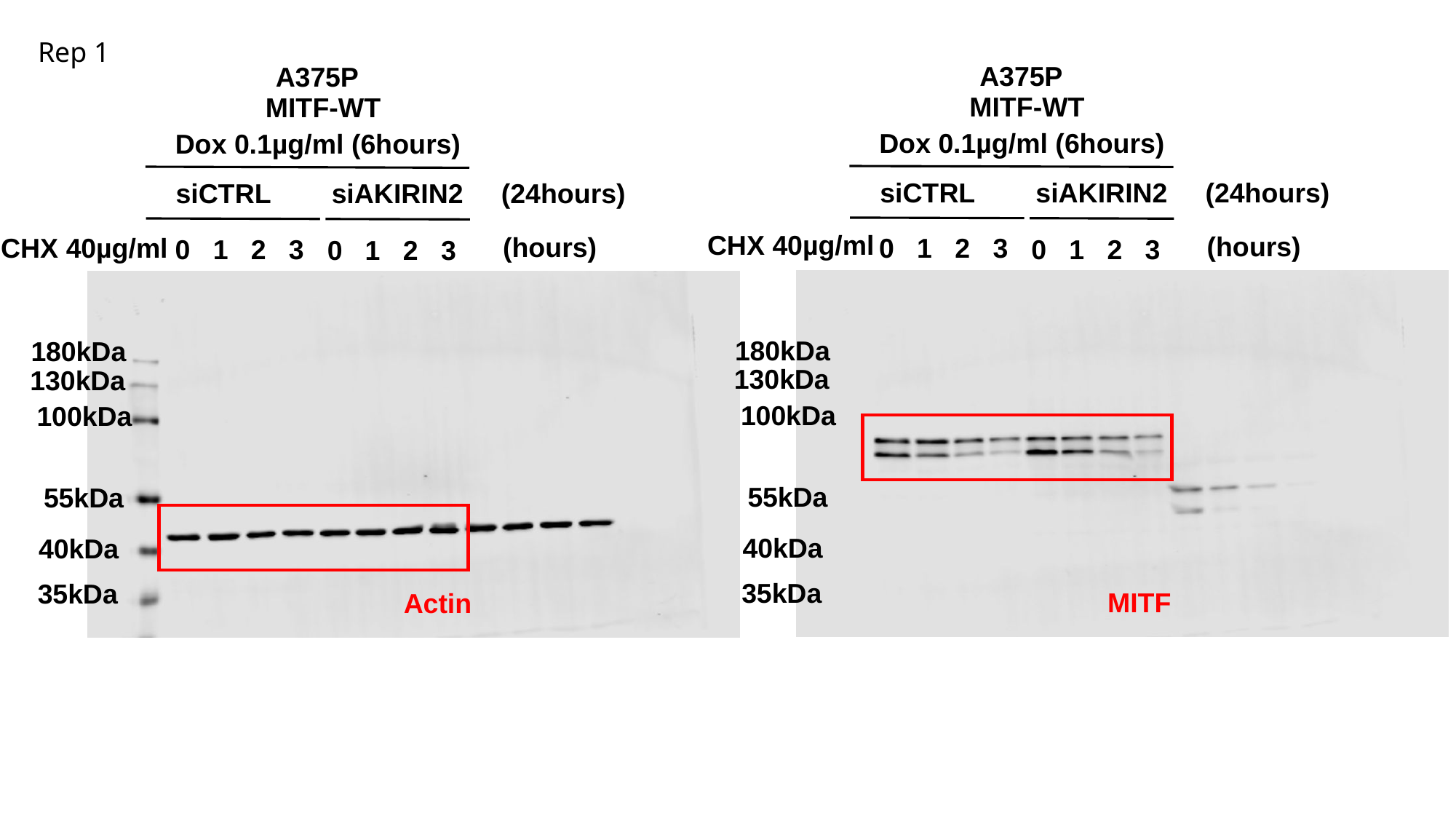

Rep 1
A375P
A375P
MITF-WT
MITF-WT
Dox 0.1µg/ml (6hours)
Dox 0.1µg/ml (6hours)
siCTRL siAKIRIN2 (24hours)
siCTRL siAKIRIN2 (24hours)
CHX 40µg/ml
(hours)
(hours)
CHX 40µg/ml
 0 1 2 3
 0 1 2 3
 0 1 2 3
 0 1 2 3
180kDa
180kDa
130kDa
130kDa
100kDa
100kDa
55kDa
55kDa
40kDa
40kDa
35kDa
35kDa
MITF
Actin

## Slide 2
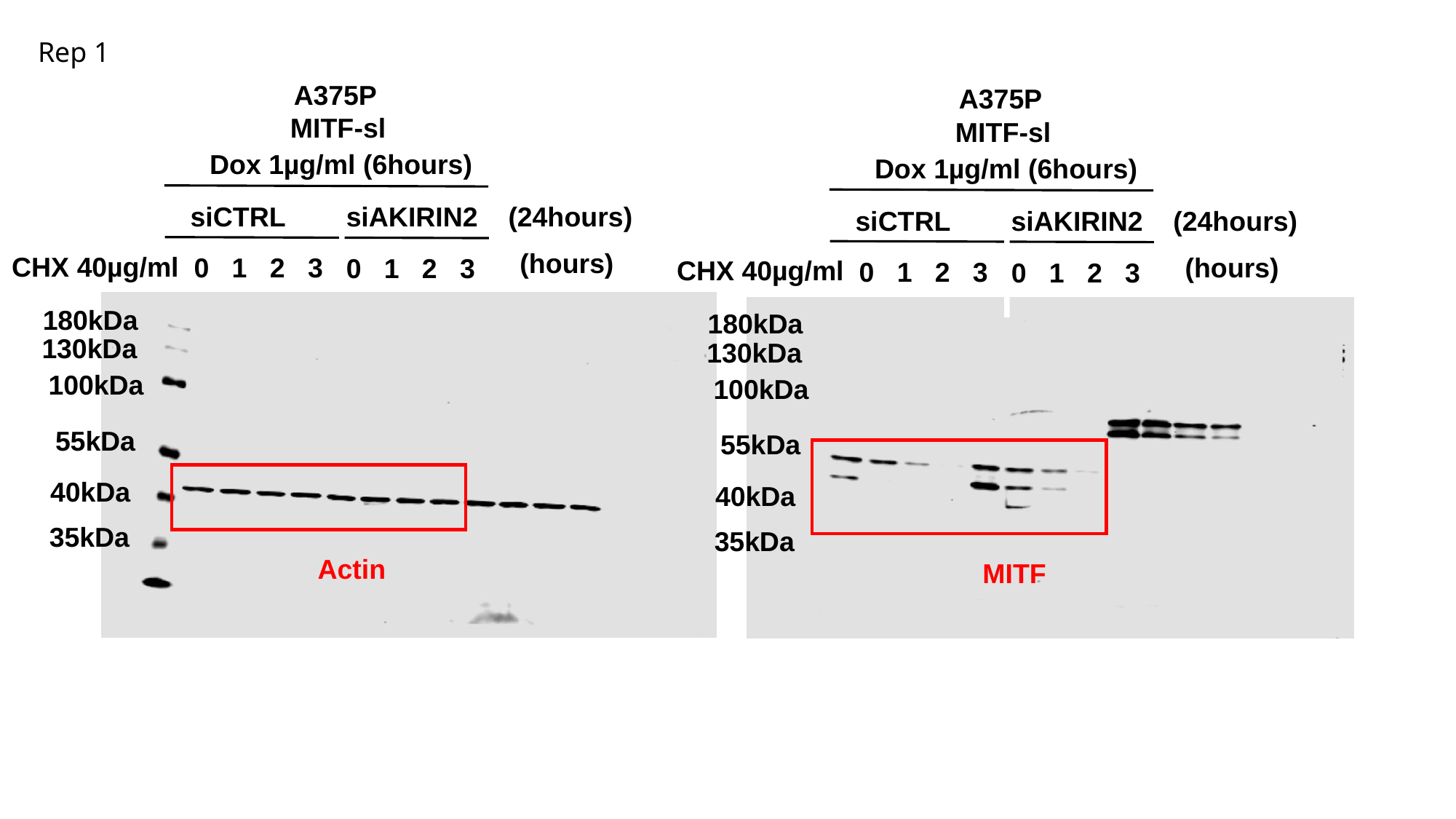

Rep 1
A375P
A375P
MITF-sl
MITF-sl
Dox 1µg/ml (6hours)
Dox 1µg/ml (6hours)
siCTRL siAKIRIN2 (24hours)
siCTRL siAKIRIN2 (24hours)
(hours)
CHX 40µg/ml
(hours)
 0 1 2 3
 0 1 2 3
CHX 40µg/ml
 0 1 2 3
 0 1 2 3
180kDa
180kDa
130kDa
130kDa
100kDa
100kDa
55kDa
55kDa
40kDa
40kDa
35kDa
35kDa
Actin
MITF

## Slide 3
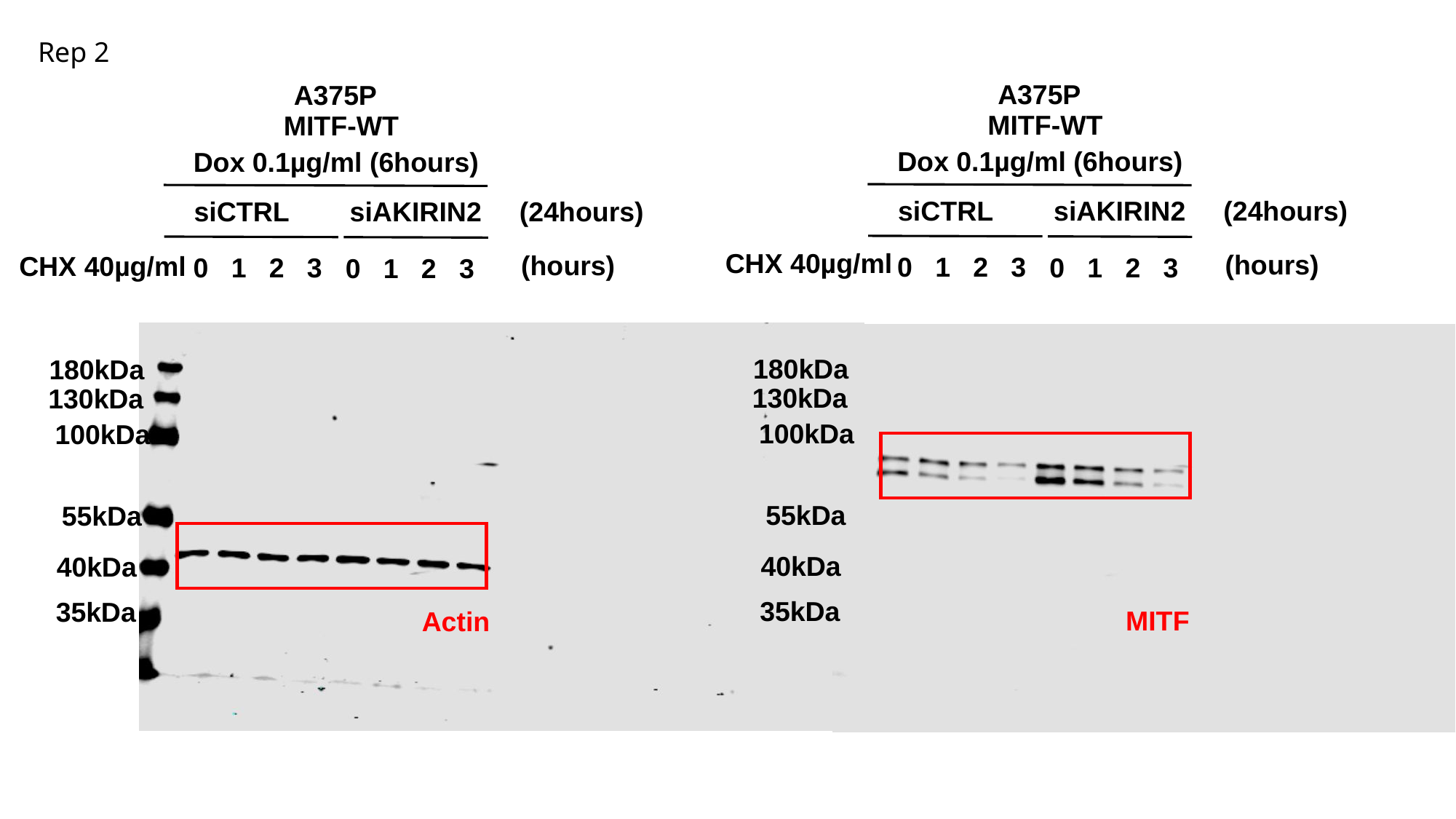

Rep 2
A375P
A375P
MITF-WT
MITF-WT
Dox 0.1µg/ml (6hours)
Dox 0.1µg/ml (6hours)
siCTRL siAKIRIN2 (24hours)
siCTRL siAKIRIN2 (24hours)
CHX 40µg/ml
(hours)
(hours)
CHX 40µg/ml
 0 1 2 3
 0 1 2 3
 0 1 2 3
 0 1 2 3
180kDa
180kDa
130kDa
130kDa
100kDa
100kDa
55kDa
55kDa
40kDa
40kDa
35kDa
35kDa
MITF
Actin

## Slide 4
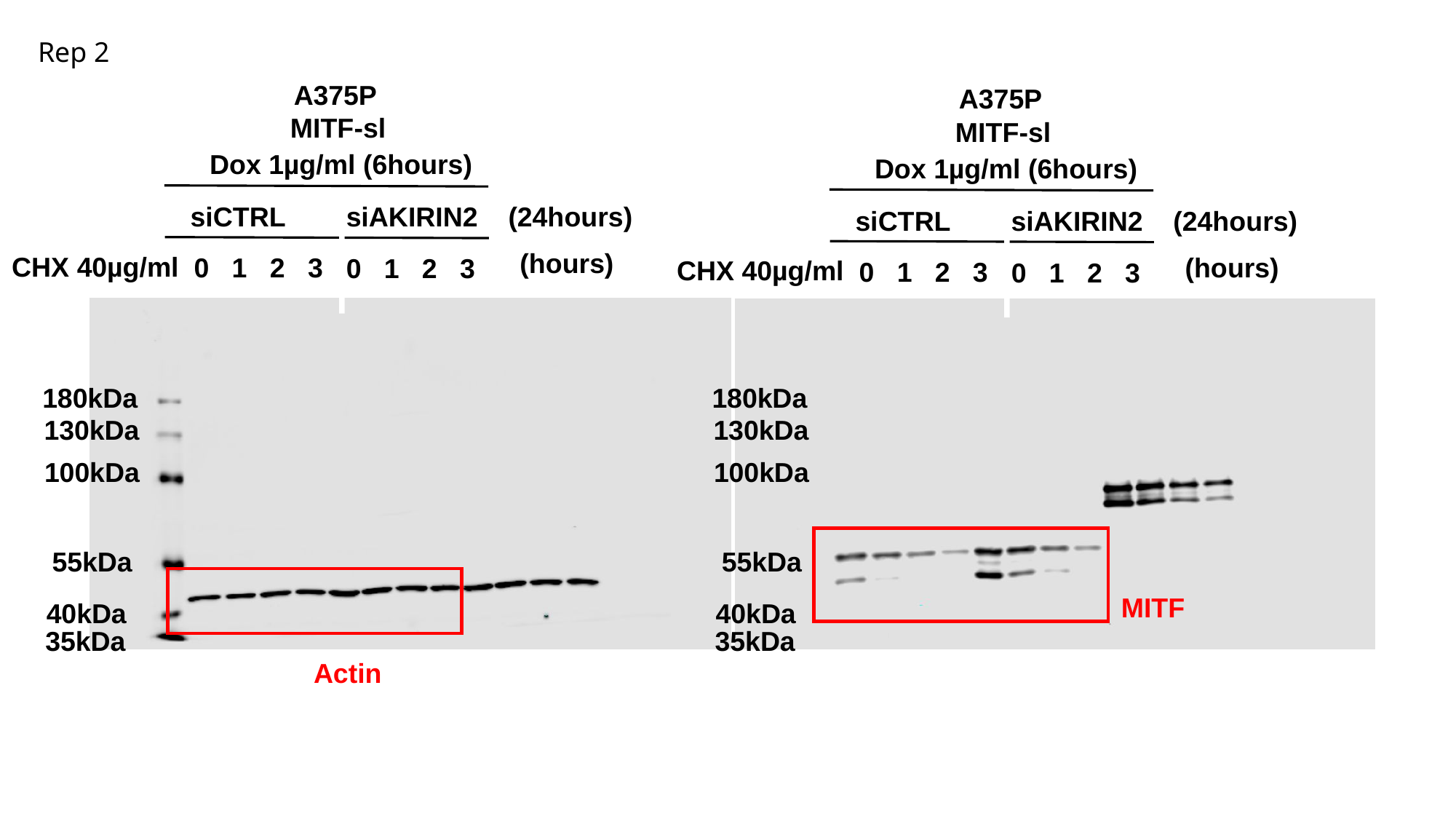

Rep 2
A375P
A375P
MITF-sl
MITF-sl
Dox 1µg/ml (6hours)
Dox 1µg/ml (6hours)
siCTRL siAKIRIN2 (24hours)
siCTRL siAKIRIN2 (24hours)
(hours)
CHX 40µg/ml
(hours)
 0 1 2 3
 0 1 2 3
CHX 40µg/ml
 0 1 2 3
 0 1 2 3
180kDa
180kDa
130kDa
130kDa
100kDa
100kDa
55kDa
55kDa
MITF
40kDa
40kDa
35kDa
35kDa
Actin

## Slide 5
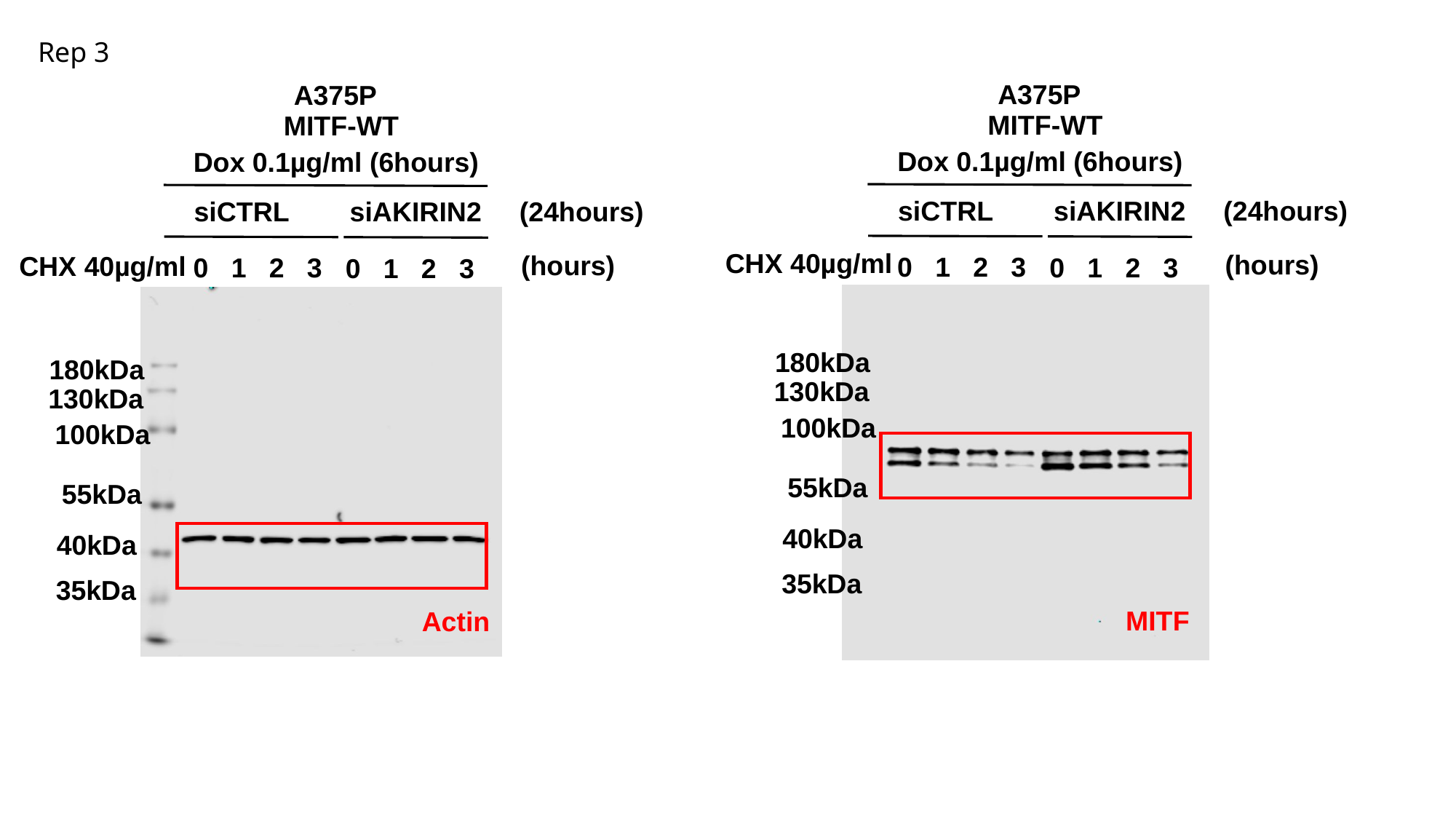

Rep 3
A375P
A375P
MITF-WT
MITF-WT
Dox 0.1µg/ml (6hours)
Dox 0.1µg/ml (6hours)
siCTRL siAKIRIN2 (24hours)
siCTRL siAKIRIN2 (24hours)
CHX 40µg/ml
(hours)
(hours)
CHX 40µg/ml
 0 1 2 3
 0 1 2 3
 0 1 2 3
 0 1 2 3
180kDa
180kDa
130kDa
130kDa
100kDa
100kDa
55kDa
55kDa
40kDa
40kDa
35kDa
35kDa
MITF
Actin

## Slide 6
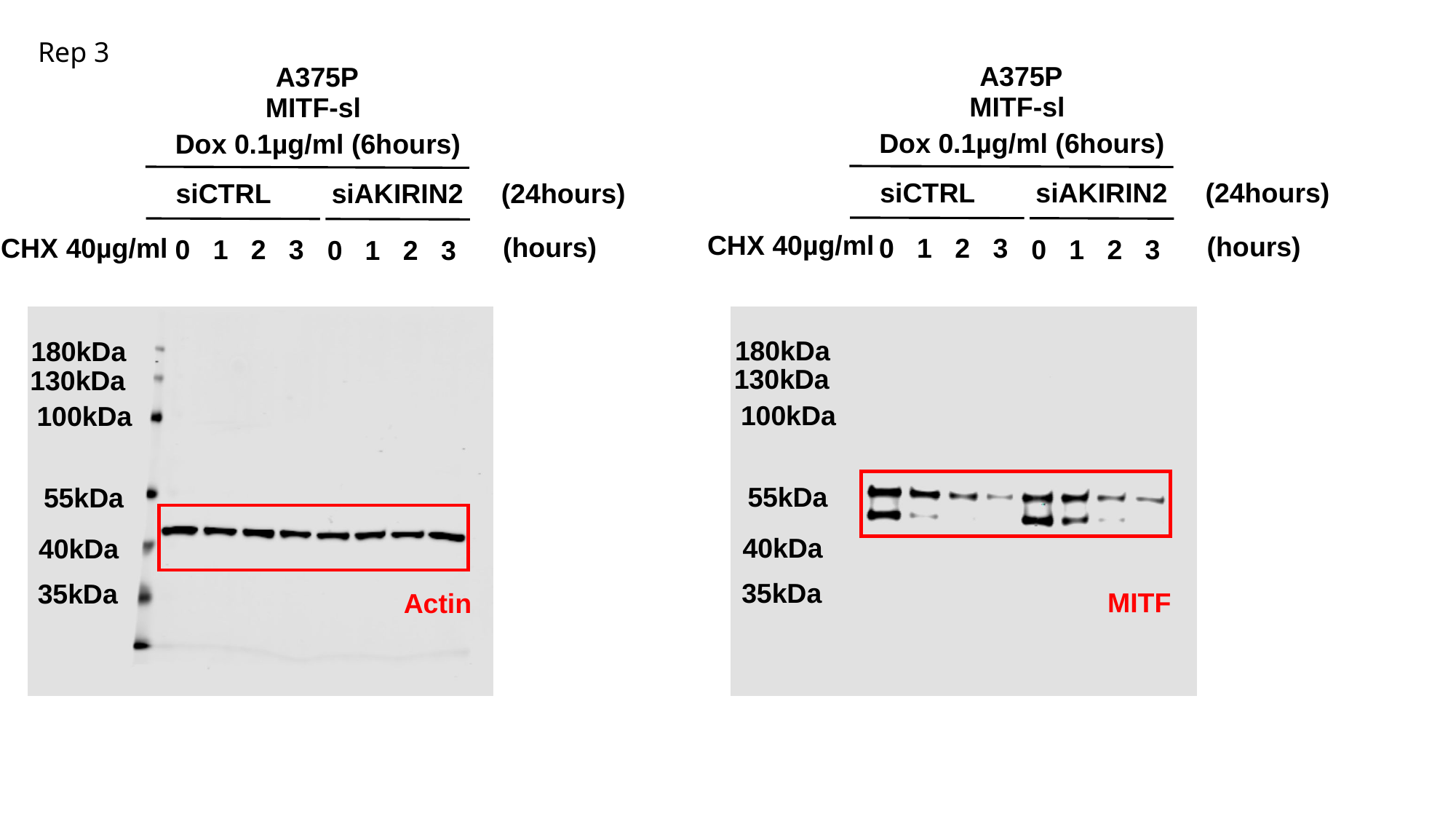

Rep 3
A375P
A375P
MITF-sl
MITF-sl
Dox 0.1µg/ml (6hours)
Dox 0.1µg/ml (6hours)
siCTRL siAKIRIN2 (24hours)
siCTRL siAKIRIN2 (24hours)
CHX 40µg/ml
(hours)
(hours)
CHX 40µg/ml
 0 1 2 3
 0 1 2 3
 0 1 2 3
 0 1 2 3
180kDa
180kDa
130kDa
130kDa
100kDa
100kDa
55kDa
55kDa
40kDa
40kDa
35kDa
35kDa
MITF
Actin
