# Supplementary material for: Novel mechanisms of MITF regulation identified in a mouse suppressor screen
Source: EMBO Rep. 2024 Aug 21;25(10):4252–80. doi: 10.1038/s44319-024-00225-3 (PMC11467436; doi:10.1038/s44319-024-00225-3)
Supplement: Supplementary file 7 — Source data Fig. 4 [file 44319_2024_225_MOESM7_ESM.zip › 4A/Figure 4A.pptx]

## Slide 1
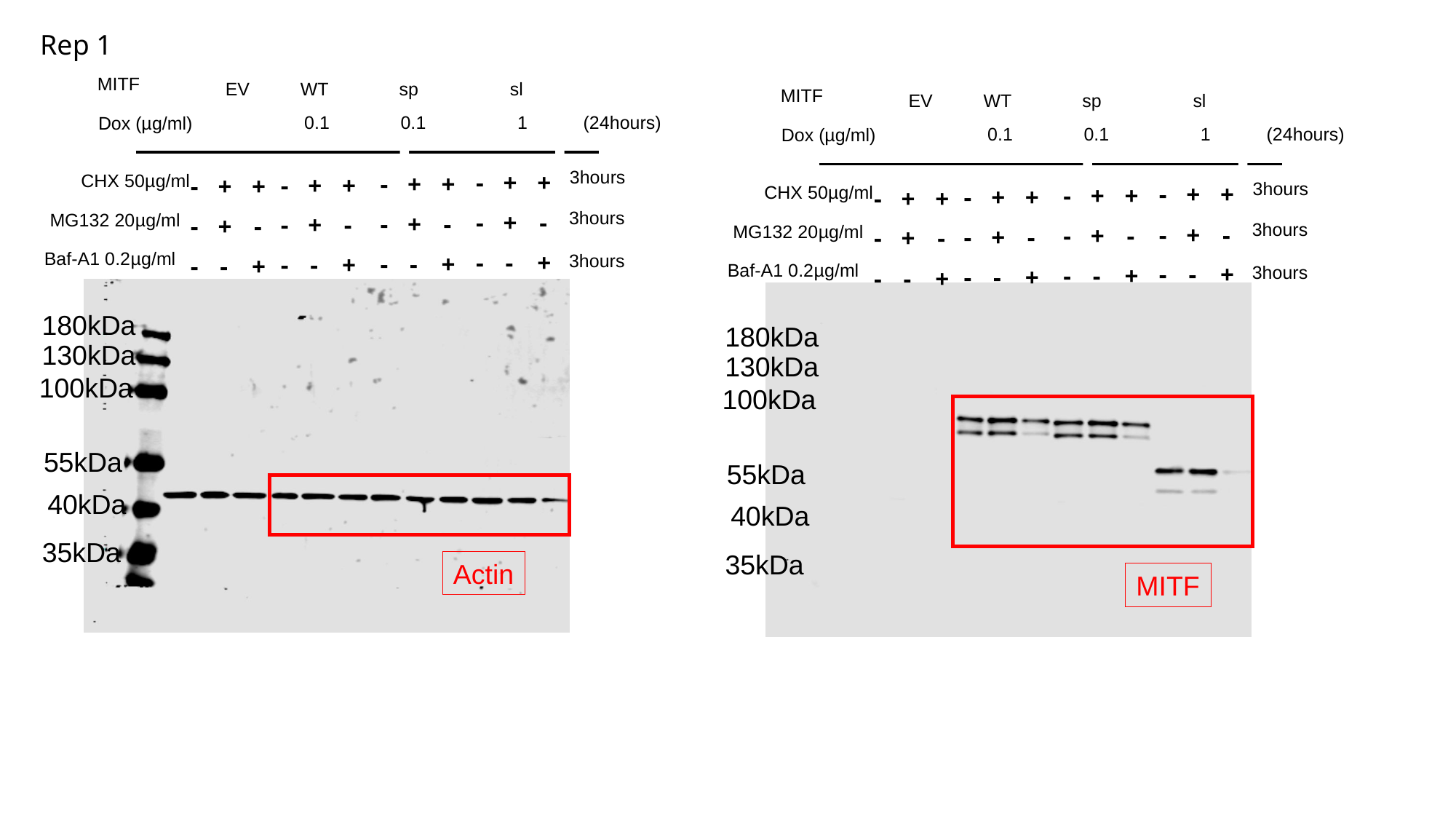

Rep 1
MITF
EV WT sp sl
MITF
EV WT sp sl
 0.1 0.1 1 (24hours)
Dox (µg/ml)
 0.1 0.1 1 (24hours)
Dox (µg/ml)
3hours
| - | + | + |
| --- | --- | --- |
| - | + | - |
| - | - | + |
| - | + | + |
| --- | --- | --- |
| - | + | - |
| - | - | + |
| - | + | + |
| --- | --- | --- |
| - | + | - |
| - | - | + |
CHX 50µg/ml
| - | + | + |
| --- | --- | --- |
| - | + | - |
| - | - | + |
3hours
| - | + | + |
| --- | --- | --- |
| - | + | - |
| - | - | + |
| - | + | + |
| --- | --- | --- |
| - | + | - |
| - | - | + |
| - | + | + |
| --- | --- | --- |
| - | + | - |
| - | - | + |
CHX 50µg/ml
| - | + | + |
| --- | --- | --- |
| - | + | - |
| - | - | + |
3hours
MG132 20µg/ml
3hours
MG132 20µg/ml
Baf-A1 0.2µg/ml
3hours
Baf-A1 0.2µg/ml
3hours
180kDa
180kDa
130kDa
130kDa
100kDa
100kDa
55kDa
55kDa
40kDa
40kDa
35kDa
35kDa
Actin
MITF

## Slide 2
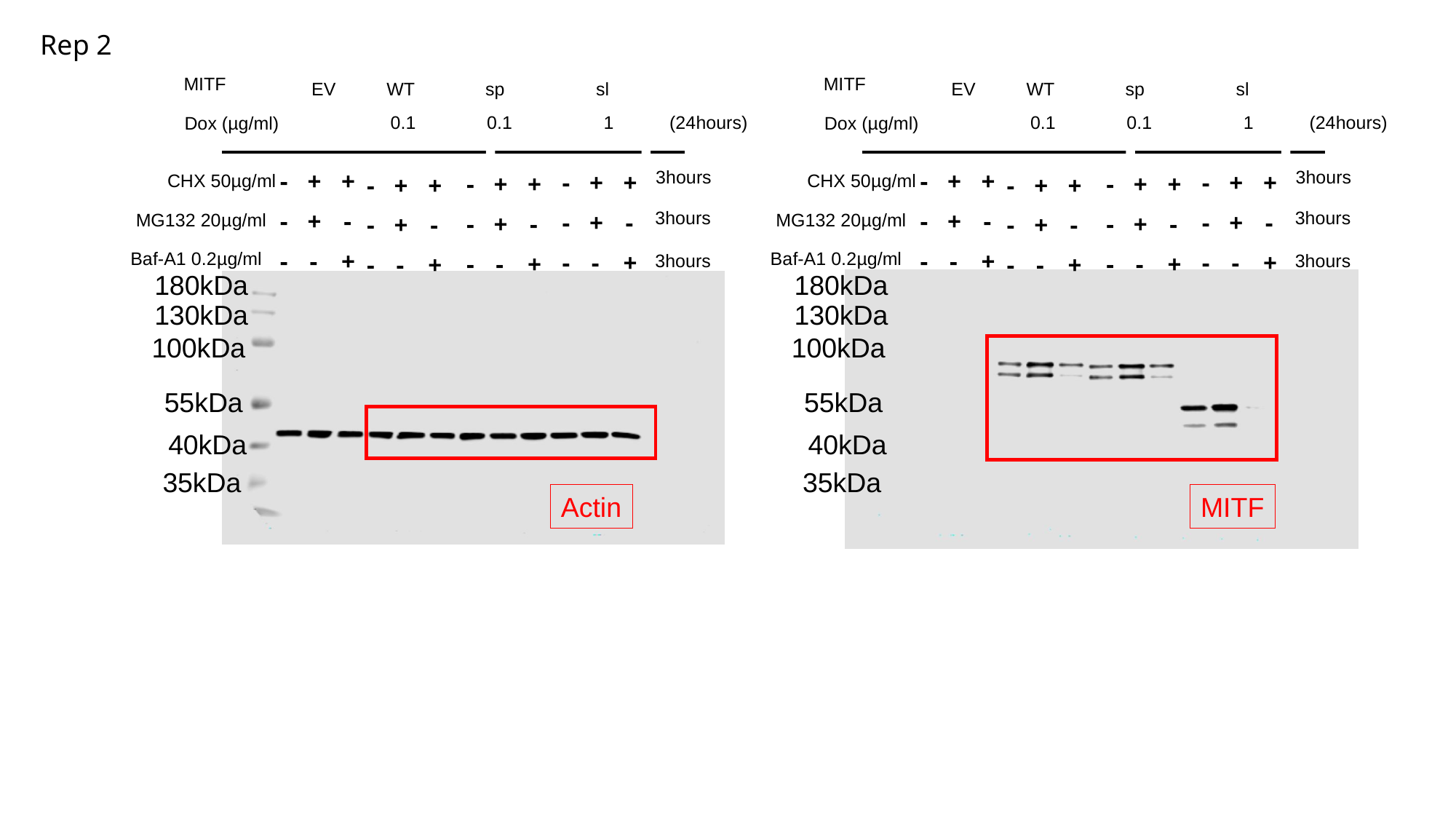

Rep 2
MITF
MITF
EV WT sp sl
EV WT sp sl
 0.1 0.1 1 (24hours)
 0.1 0.1 1 (24hours)
Dox (µg/ml)
Dox (µg/ml)
| - | + | + |
| --- | --- | --- |
| - | + | - |
| - | - | + |
| - | + | + |
| --- | --- | --- |
| - | + | - |
| - | - | + |
3hours
3hours
| - | + | + |
| --- | --- | --- |
| - | + | - |
| - | - | + |
| - | + | + |
| --- | --- | --- |
| - | + | - |
| - | - | + |
| - | + | + |
| --- | --- | --- |
| - | + | - |
| - | - | + |
| - | + | + |
| --- | --- | --- |
| - | + | - |
| - | - | + |
| - | + | + |
| --- | --- | --- |
| - | + | - |
| - | - | + |
| - | + | + |
| --- | --- | --- |
| - | + | - |
| - | - | + |
CHX 50µg/ml
CHX 50µg/ml
3hours
3hours
MG132 20µg/ml
MG132 20µg/ml
Baf-A1 0.2µg/ml
Baf-A1 0.2µg/ml
3hours
3hours
180kDa
180kDa
130kDa
130kDa
100kDa
100kDa
55kDa
55kDa
40kDa
40kDa
35kDa
35kDa
Actin
MITF

## Slide 3
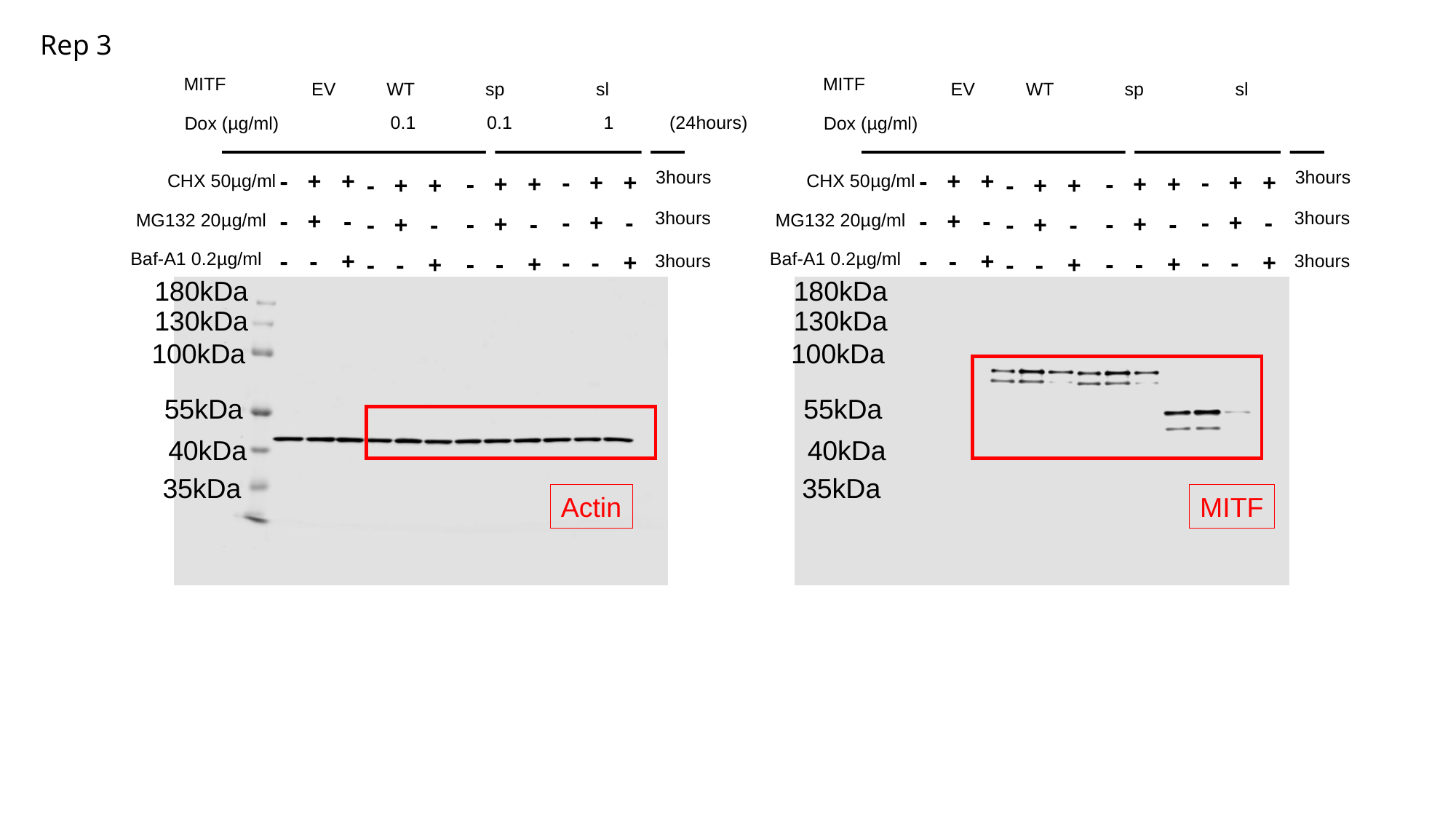

Rep 3
MITF
MITF
EV WT sp sl
EV WT sp sl
 0.1 0.1 1 (24hours)
Dox (µg/ml)
Dox (µg/ml)
| - | + | + |
| --- | --- | --- |
| - | + | - |
| - | - | + |
| - | + | + |
| --- | --- | --- |
| - | + | - |
| - | - | + |
3hours
3hours
| - | + | + |
| --- | --- | --- |
| - | + | - |
| - | - | + |
| - | + | + |
| --- | --- | --- |
| - | + | - |
| - | - | + |
| - | + | + |
| --- | --- | --- |
| - | + | - |
| - | - | + |
| - | + | + |
| --- | --- | --- |
| - | + | - |
| - | - | + |
| - | + | + |
| --- | --- | --- |
| - | + | - |
| - | - | + |
| - | + | + |
| --- | --- | --- |
| - | + | - |
| - | - | + |
CHX 50µg/ml
CHX 50µg/ml
3hours
3hours
MG132 20µg/ml
MG132 20µg/ml
Baf-A1 0.2µg/ml
Baf-A1 0.2µg/ml
3hours
3hours
180kDa
180kDa
130kDa
130kDa
100kDa
100kDa
55kDa
55kDa
40kDa
40kDa
35kDa
35kDa
Actin
MITF
