# Supplementary material for: Novel mechanisms of MITF regulation identified in a mouse suppressor screen
Source: EMBO Rep. 2024 Aug 21;25(10):4252–80. doi: 10.1038/s44319-024-00225-3 (PMC11467436; doi:10.1038/s44319-024-00225-3)
Supplement: Supplementary file 8 — Source data Fig. 5 [file 44319_2024_225_MOESM8_ESM.zip › 5C/Figure 5C.pptx]

## Slide 1
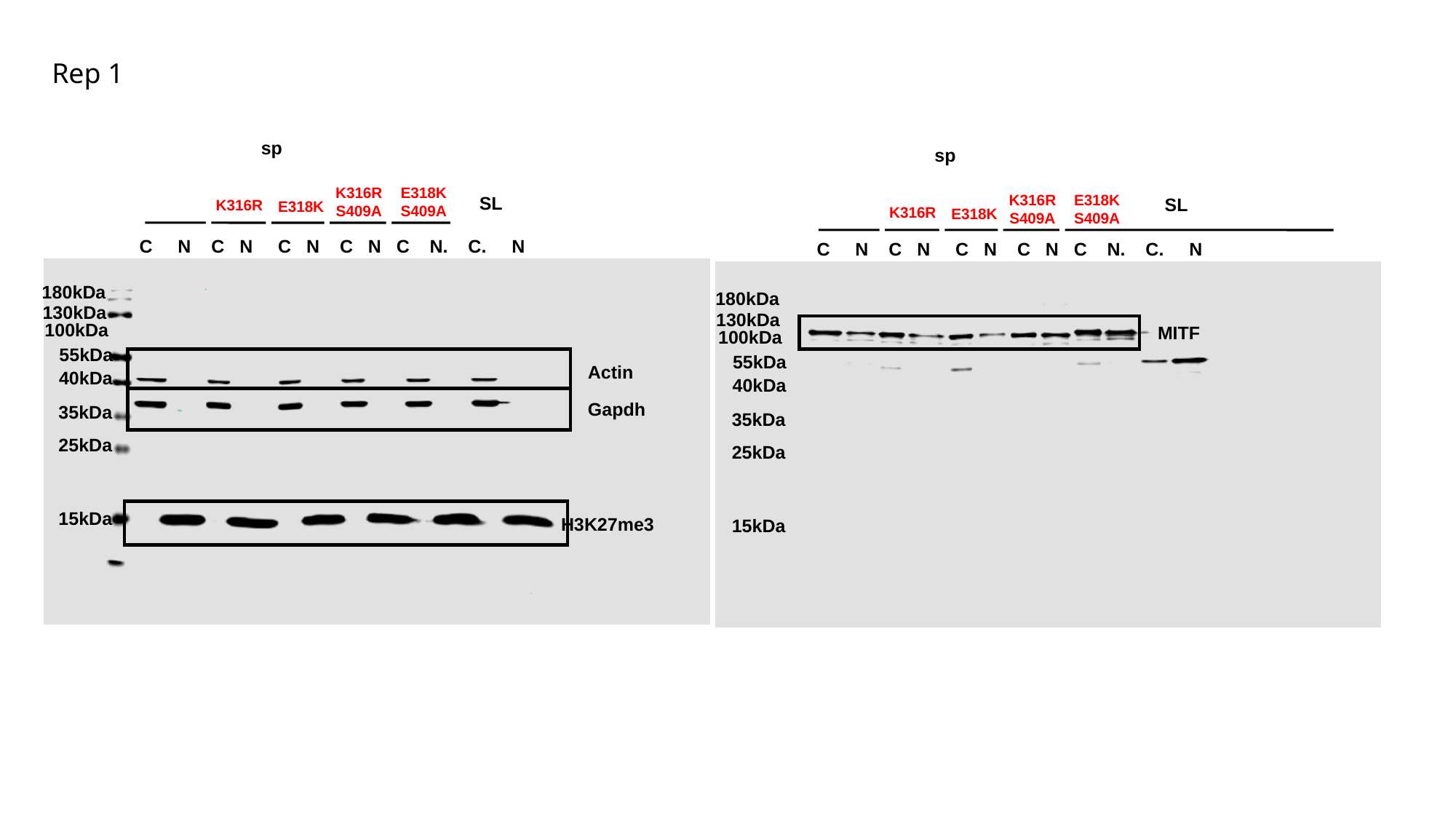

Rep 1
c
c
c
c
c
sp
c
c
sp
c
c
E318K
S409A
K316R
S409A
E318K
S409A
K316R
S409A
SL
SL
K316R
E318K
K316R
E318K
 C N C N C N C N C N. C. N
 C N C N C N C N C N. C. N
180kDa
180kDa
130kDa
130kDa
100kDa
MITF
100kDa
55kDa
55kDa
Actin
40kDa
40kDa
Gapdh
35kDa
35kDa
25kDa
25kDa
15kDa
H3K27me3
15kDa

## Slide 2
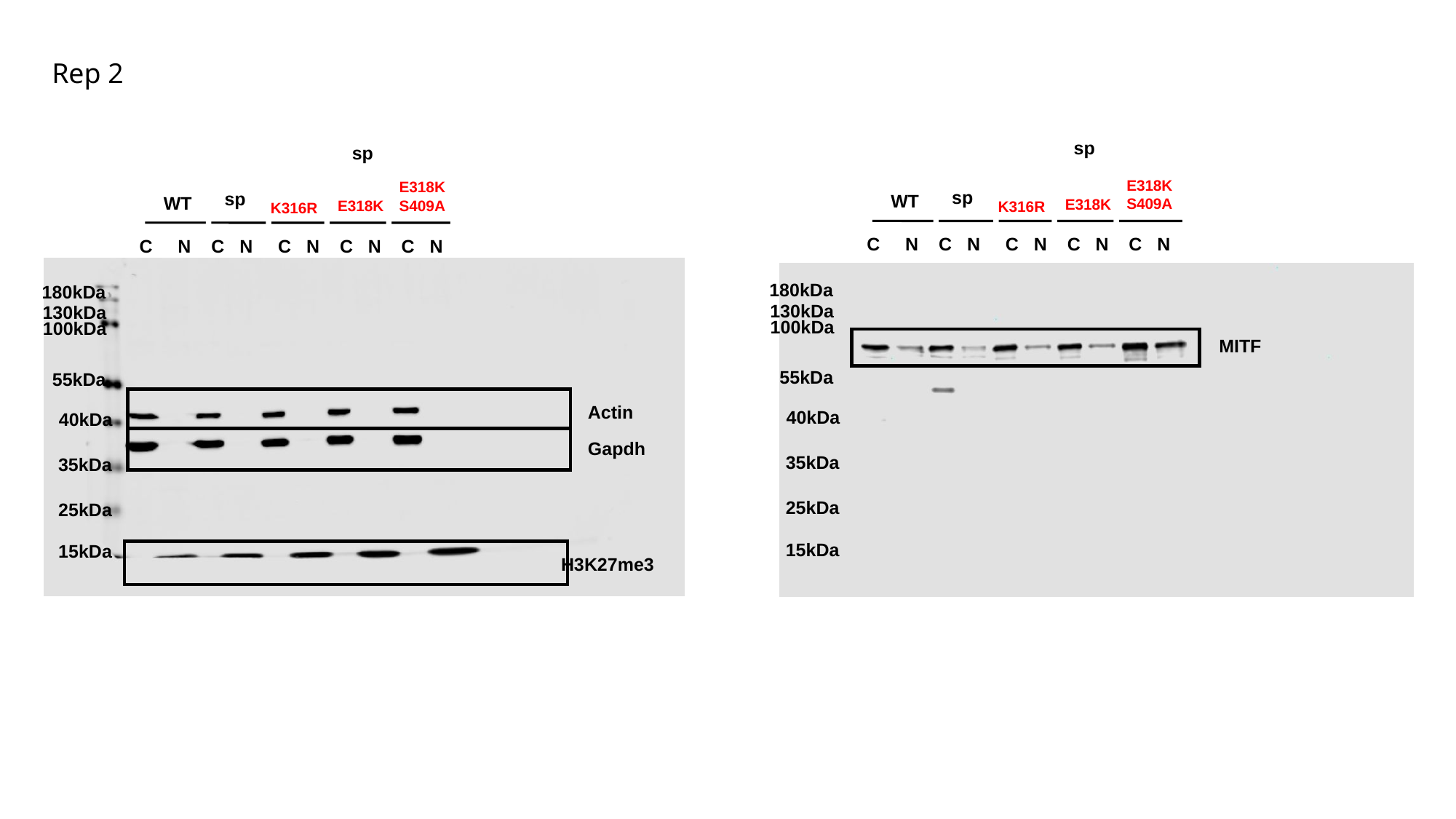

Rep 2
c
c
c
c
c
c
c
sp
c
c
sp
c
E318K
S409A
E318K
S409A
sp
sp
WT
WT
E318K
E318K
 K316R
 K316R
 C N C N C N C N C N
 C N C N C N C N C N
180kDa
180kDa
130kDa
130kDa
100kDa
100kDa
MITF
55kDa
55kDa
Actin
40kDa
40kDa
Gapdh
35kDa
35kDa
25kDa
25kDa
15kDa
15kDa
H3K27me3

## Slide 3
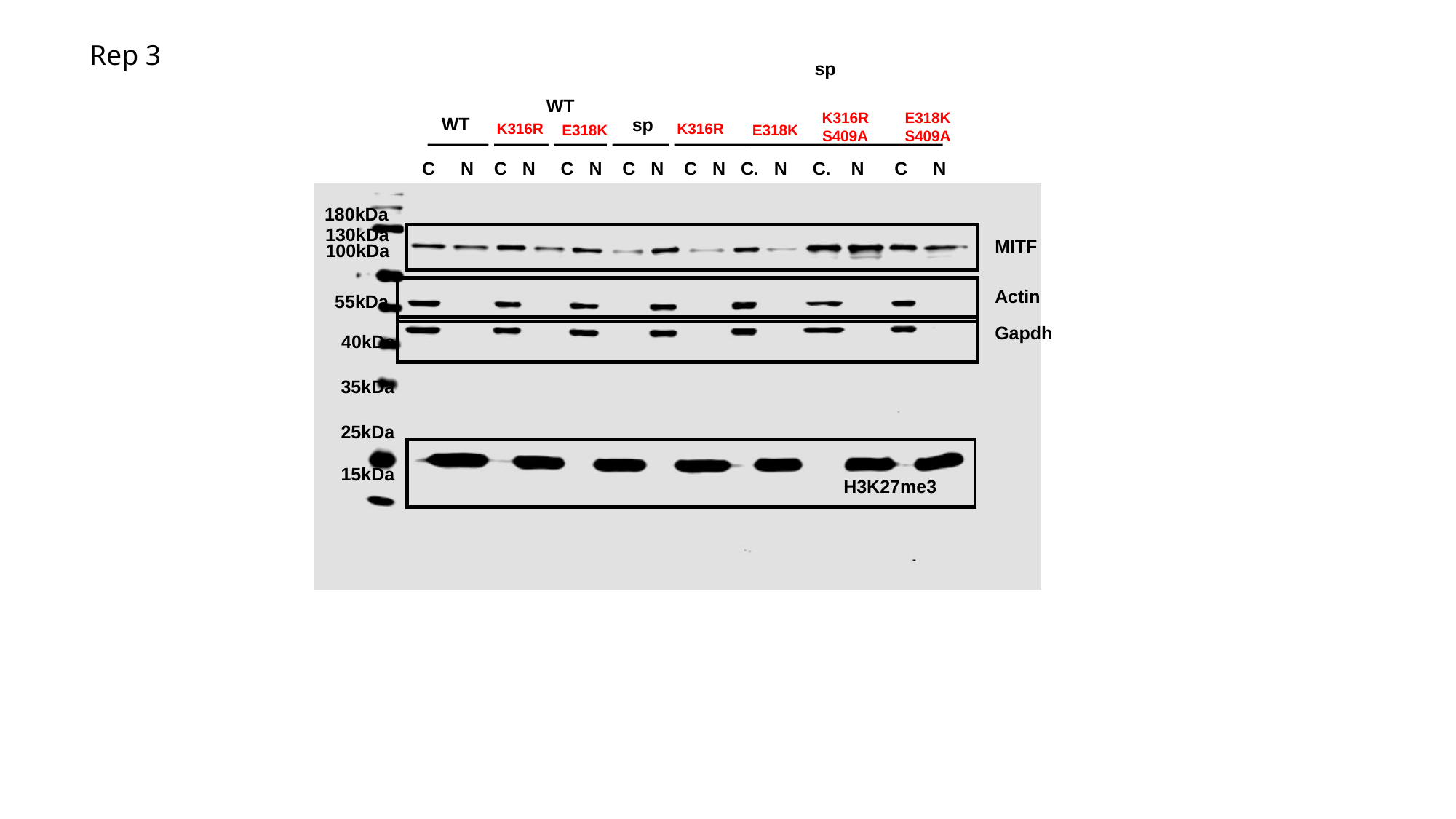

Rep 3
c
c
c
sp
c
c
WT
K316R
S409A
E318K
S409A
WT
sp
 K316R
 K316R
E318K
E318K
 C N C N C N C N C N C. N C. N C N
180kDa
130kDa
MITF
100kDa
Actin
55kDa
Gapdh
40kDa
35kDa
25kDa
15kDa
H3K27me3

## Slide 4
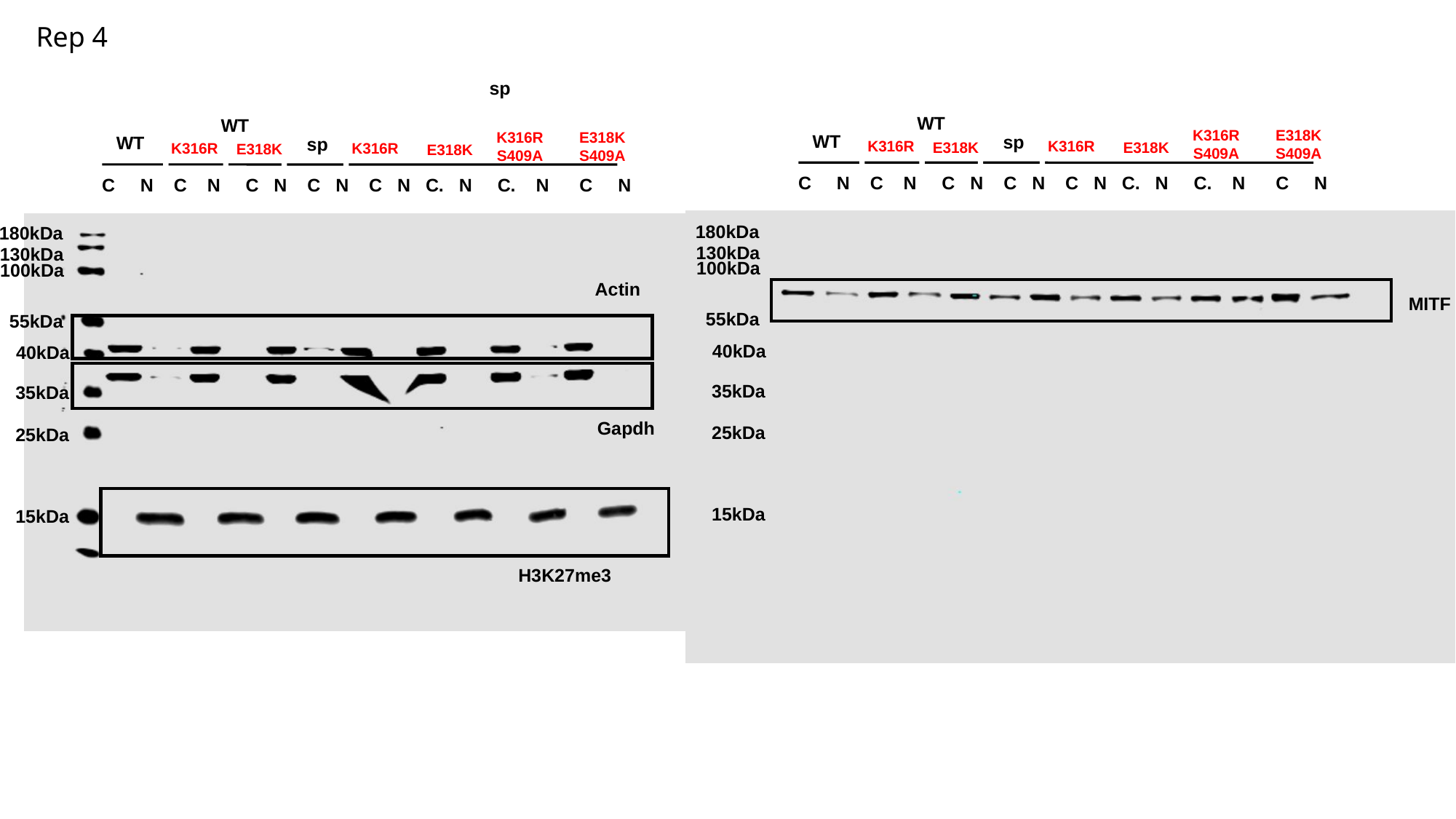

Rep 4
c
c
c
c
c
c
sp
c
c
c
WT
WT
K316R
S409A
E318K
S409A
K316R
S409A
E318K
S409A
WT
sp
WT
sp
 K316R
 K316R
E318K
E318K
 K316R
 K316R
E318K
E318K
 C N C N C N C N C N C. N C. N C N
 C N C N C N C N C N C. N C. N C N
180kDa
180kDa
130kDa
130kDa
100kDa
100kDa
Actin
MITF
55kDa
55kDa
40kDa
40kDa
35kDa
35kDa
Gapdh
25kDa
25kDa
15kDa
15kDa
H3K27me3
