# Supplementary material for: Novel mechanisms of MITF regulation identified in a mouse suppressor screen
Source: EMBO Rep. 2024 Aug 21;25(10):4252–80. doi: 10.1038/s44319-024-00225-3 (PMC11467436; doi:10.1038/s44319-024-00225-3)
Supplement: Supplementary file 8 — Source data Fig. 5 [file 44319_2024_225_MOESM8_ESM.zip › 5A/Figure 5A.pptx]

## Slide 1
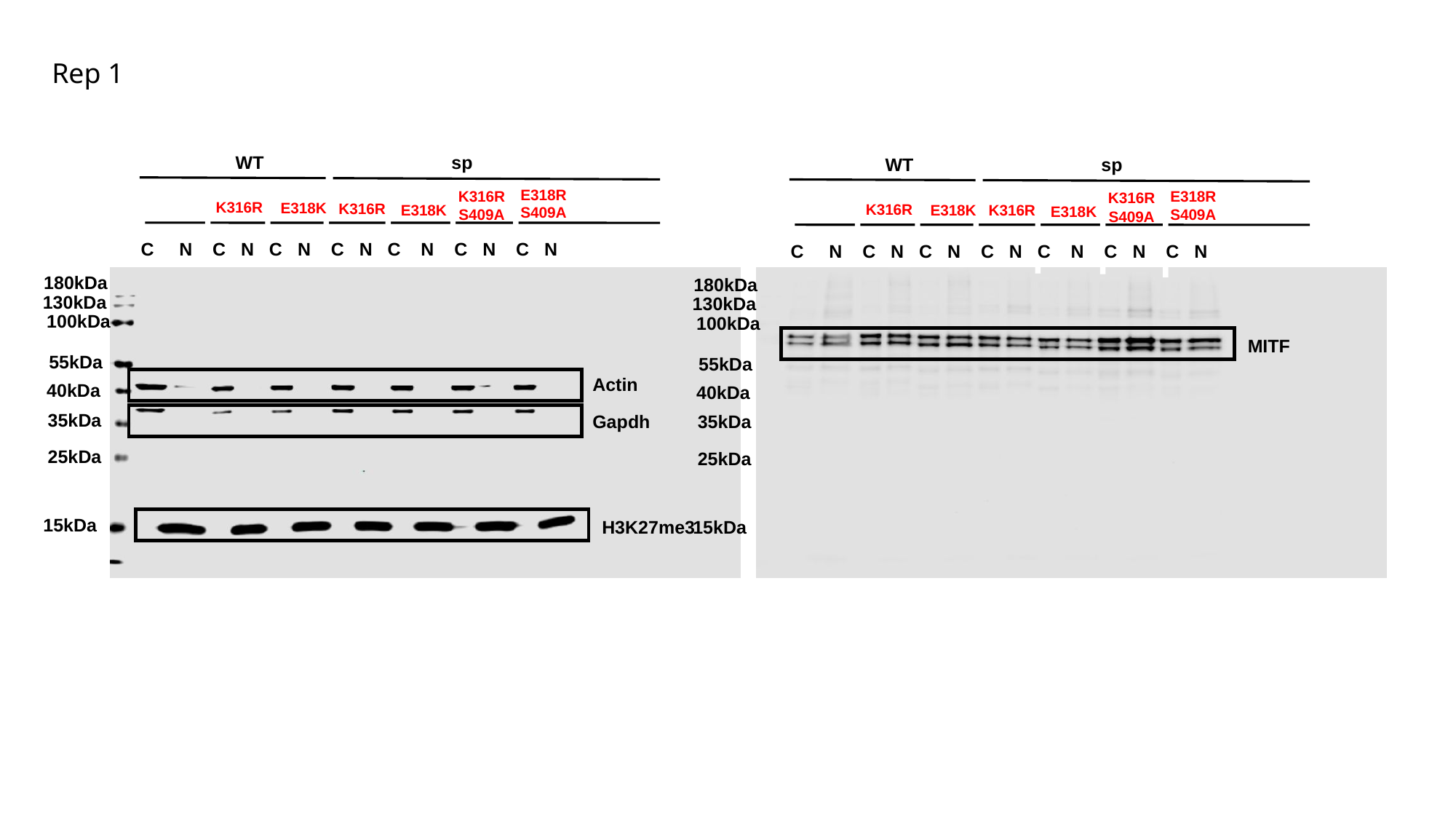

Rep 1
c
c
c
c
c
c
WT sp
WT sp
c
c
c
c
c
c
E318R
S409A
K316R
S409A
E318R
S409A
K316R
S409A
K316R
E318K
K316R
K316R
E318K
E318K
K316R
E318K
 C N C N C N C N C N C N C N
 C N C N C N C N C N C N C N
180kDa
180kDa
130kDa
130kDa
100kDa
100kDa
MITF
55kDa
55kDa
Actin
40kDa
40kDa
35kDa
Gapdh
35kDa
25kDa
25kDa
15kDa
H3K27me3
15kDa

## Slide 2
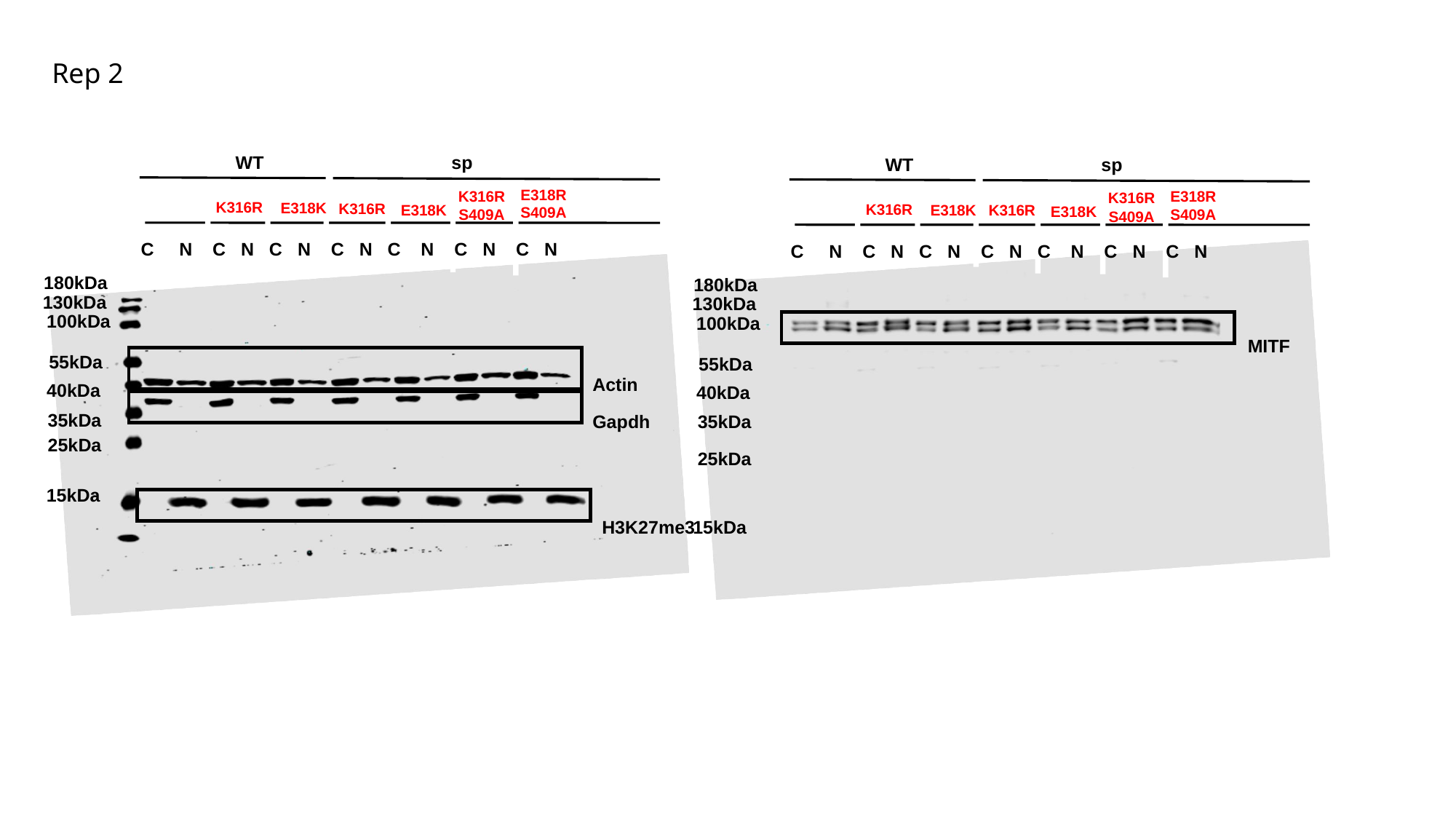

Rep 2
c
c
c
c
c
c
WT sp
WT sp
c
c
c
c
c
c
E318R
S409A
K316R
S409A
E318R
S409A
K316R
S409A
K316R
E318K
K316R
K316R
E318K
E318K
K316R
E318K
 C N C N C N C N C N C N C N
 C N C N C N C N C N C N C N
180kDa
180kDa
130kDa
130kDa
100kDa
100kDa
MITF
55kDa
55kDa
Actin
40kDa
40kDa
35kDa
Gapdh
35kDa
25kDa
25kDa
15kDa
H3K27me3
15kDa

## Slide 3
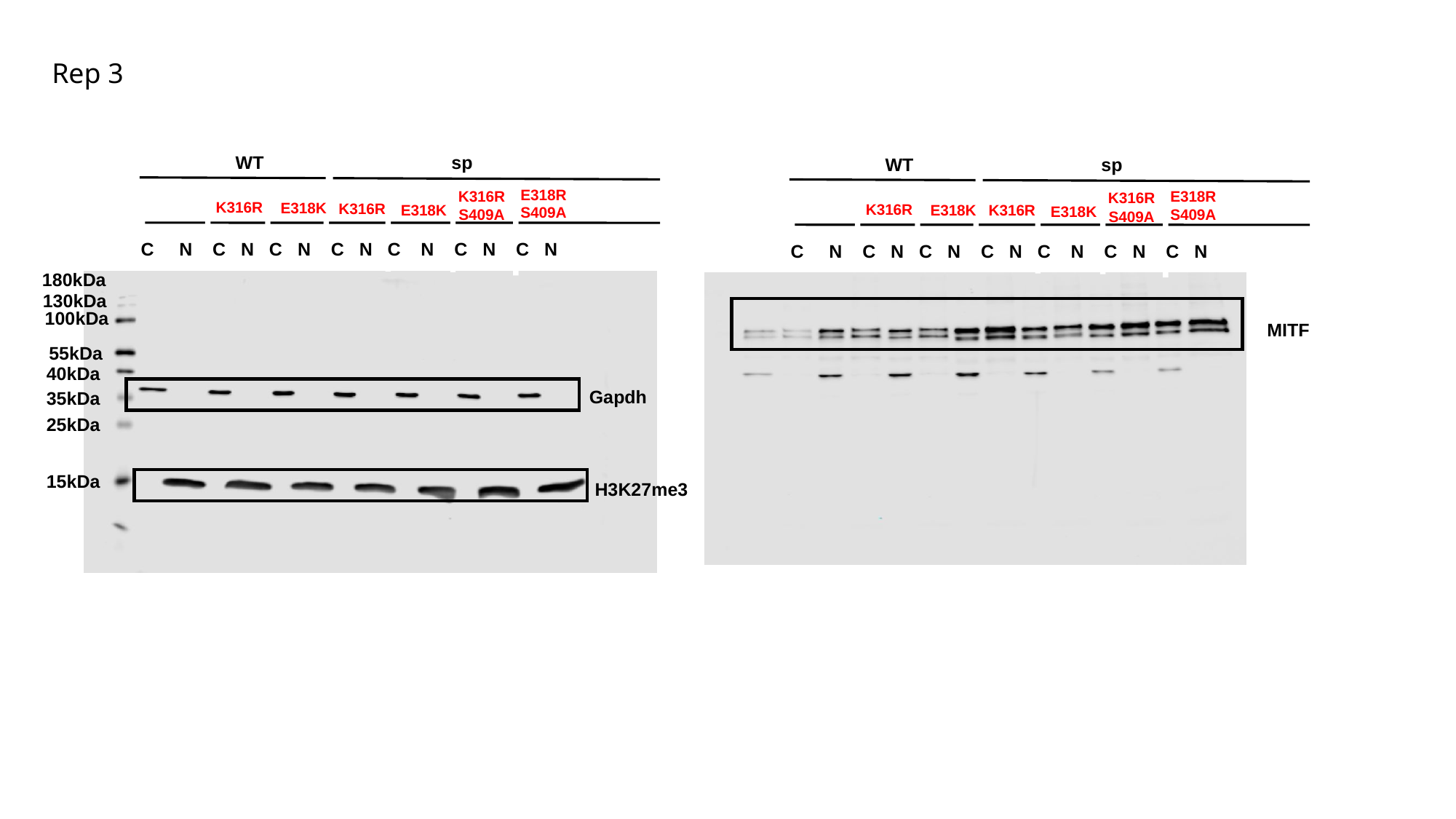

Rep 3
c
c
c
c
c
c
WT sp
WT sp
c
c
c
c
c
c
E318R
S409A
K316R
S409A
E318R
S409A
K316R
S409A
K316R
E318K
K316R
K316R
E318K
E318K
K316R
E318K
 C N C N C N C N C N C N C N
 C N C N C N C N C N C N C N
180kDa
130kDa
100kDa
MITF
55kDa
40kDa
Gapdh
35kDa
25kDa
15kDa
H3K27me3
