# Supplementary material for: Novel mechanisms of MITF regulation identified in a mouse suppressor screen
Source: EMBO Rep. 2024 Aug 21;25(10):4252–80. doi: 10.1038/s44319-024-00225-3 (PMC11467436; doi:10.1038/s44319-024-00225-3)
Supplement: Supplementary file 10 — Expanded View Figures [file 44319_2024_225_MOESM10_ESM.pdf]

Expanded View Figures

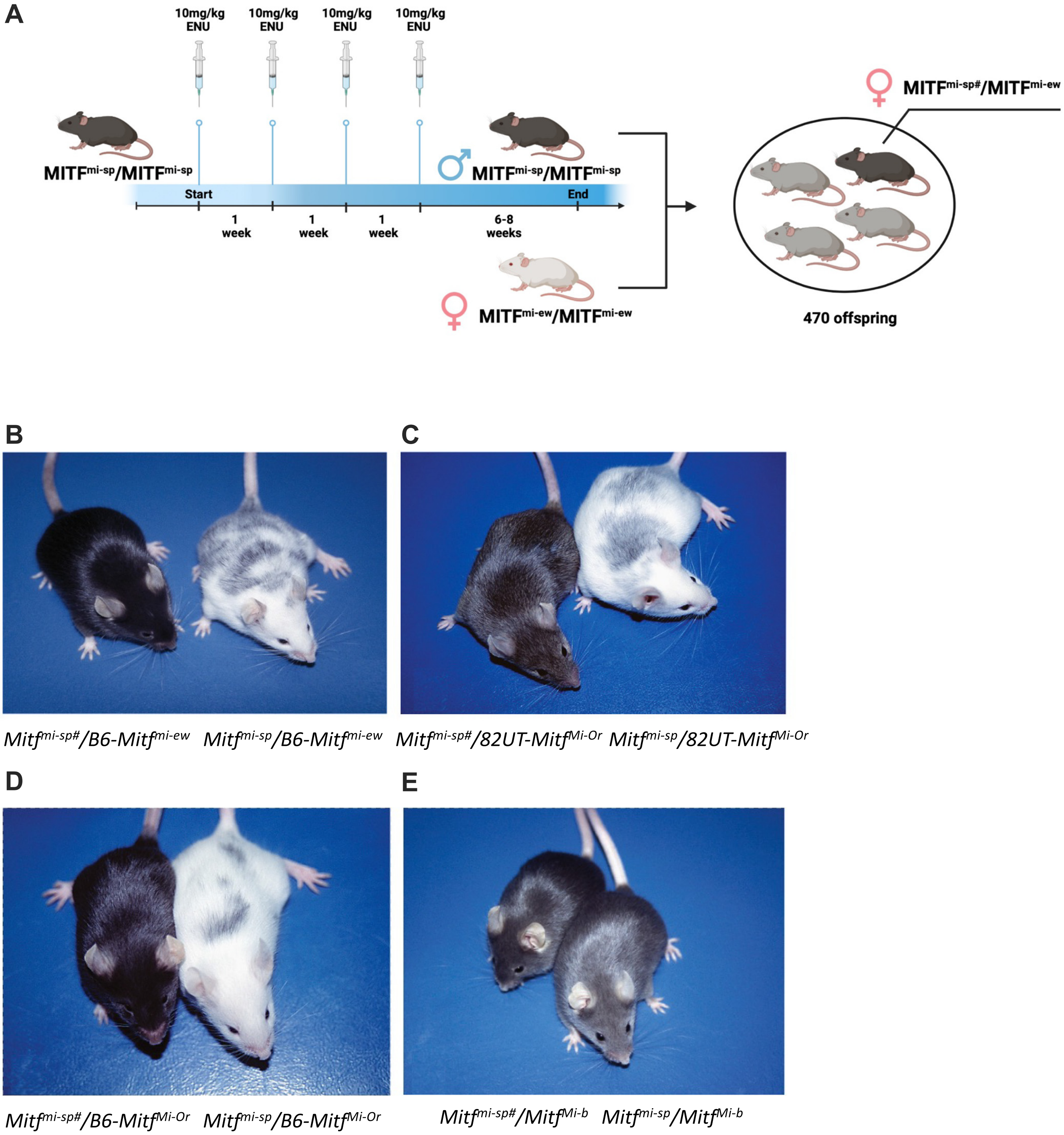

Figure EV1. Generation and phenotypic behavior of the induced *Mitf*$^{mi-sp\#}$ suppressor mutation.

(**A**) Schematic of generation of a *Mitf* suppressor mutation in mouse. (**B**) B6-*Mitf*$^{mi-ew}$/B6-*Mitf*$^{mi-sp\#}$ and B6-*Mitf*$^{mi-ew}$/B6-*Mitf*$^{mi-sp}$ compound heterozygotes. (**C**) 82UT-*Mitf*$^{Mi-Or}$/B6-*Mitf*$^{mi-sp\#}$ and 82UT-*Mitf*$^{Mi-Or}$/B6-*Mitf*$^{mi-sp}$ compound heterozygotes. (**D**) B6-Mitf$^{mi-sp\#}$/B6-Mitf^{Mi-Or} and B6-Mitf^{mi-sp}/B6-Mitf^{Mi-Or} compound heterozygotes. (**E**) B6-Mitf$^{mi-sp\#}$/B6-Mitf^{Mi-b} and B6-Mitf^{mi-sp}/B6-Mitf^{Mi-b} animals.

© The Author(s)

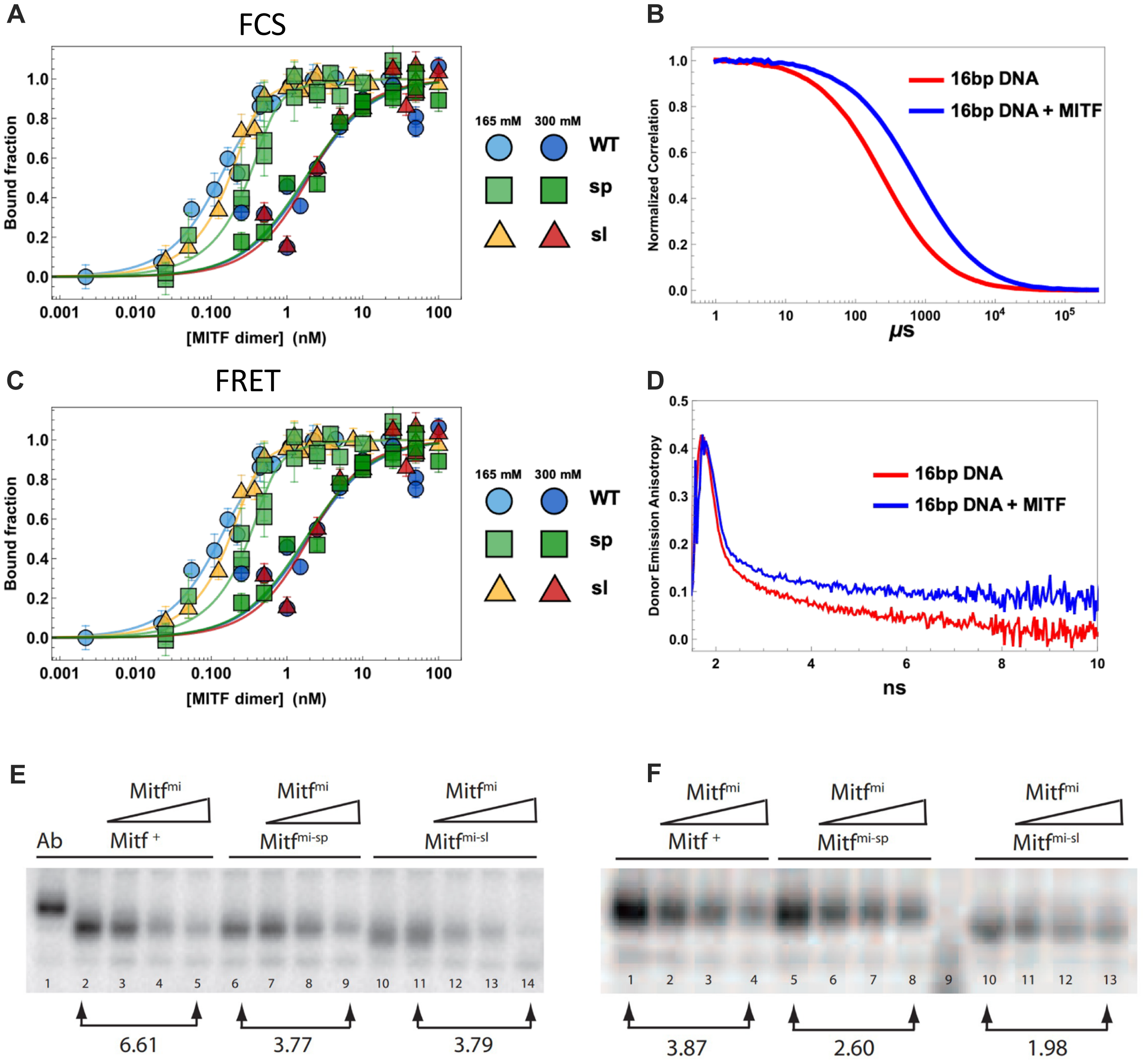

Figure EV2. The MITF-sl protein has similar DNA binding affinity, however, prefers to form dimers compared to MITF-WT and MITF-sp.

(**A**) DNA binding curves of recombinantly expressed human MITF-WT, MITF-sp, and MITF-sl protein to M-box probe measured by Fluorescence Correlation Spectroscopy (FCS) at 165 mM KCl (blue) and 300 mM KCl (yellow). MITF-WT protein in circles, MITF-sp square boxes, and MITF-sl in triangles. Error bars represent two standard deviations of fit error at each point. (**B**) Normalized donor-acceptor fluorescence cross-correlation curves of 16bp M-Box DNA alone (red) and with 100 nM WT MITF added (blue), both at 300 mM KCl. (**C**) DNA binding curves of recombinantly expressed human MITF-WT, MITF-sp, and MITF-sl protein to M-box probe measured by mean FRET at 165 mM KCl (blue) and 300 mM KCl (yellow). MITF-WT protein in circles, MITF-sp square boxes, and MITF-sl in triangles. Error bars represent two standard deviations of fit error at each point. (**D**) Inverted time-correlated donor emission anisotropy of 16 bp DNA alone (red) and in the presence of 100 nM MITF WT (blue), both at 300 mM KCl. (**E**, **F**) Electrophoretic mobility shift assays were performed using the M-box sequence (5′-AAAGTCAGTCATGTGCTTTTCAGA-3′) as a probe. (**E**) MITF-WT, MITF-sp, and MITF-sl proteins were expressed using the TNT (Promega) system alone (lanes 1, 2, 6, 10, and 11) or co-expressed with the dominant-negative MITF-mi protein (lanes 3–5, 7–9, and 12–14) and then incubated with the labeled probe. The binding is specific since the presence of the C5 monoclonal MITF antibody which recognizes the N-terminus of Mitf results in a supershift (Ab). (**F**) The same experiment as in (**E**), except that the proteins were translated separately and then incubated for 30 min in the presence of DNA to allow heterodimerization before performing the mobility shift experiment.

 © The Author(s)

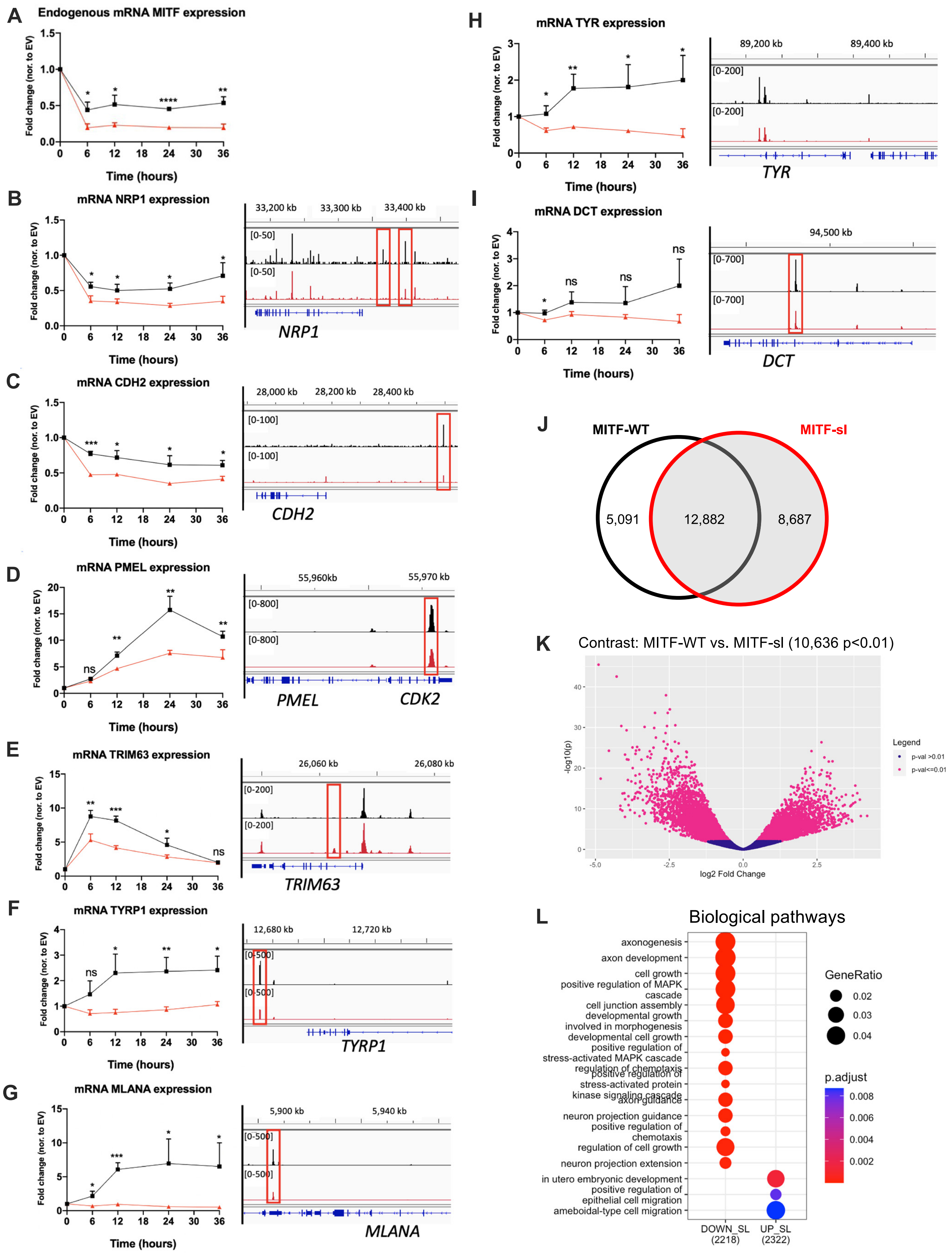
A
Endogenous mRNA MITF expression
B
mRNA NRP1 expression
NRP1
C
mRNA CDH2 expression
CDH2
D
mRNA PMEL expression
PMEL
CDK2
E
mRNA TRIM63 expression
TRIM63
F
mRNA TYRP1 expression
TYRP1
G
mRNA MLANA expression
MLANA
H
mRNA TYR expression
TYR
I
mRNA DCT expression
DCT
Fold change (nor. to EV)
Time (hours)
J
MITF-WT
MITF-sl
5,091
12,882
8,687
K
Contrast: MITF-WT vs. MITF-sl (10,636 p<0.01)
-log10(p)
log2 Fold Change
Legend
p-val >0.01
p-val<=0.01
L
Biological pathways
axonogenesis
axon development
cell growth
positive regulation of MAPK cascade
cell junction assembly
developmental growth involved in morphogenesis
developmental cell growth
positive regulation of stress-activated MAPK cascade
regulation of chemotaxis
positive regulation of stress-activated protein kinase signaling cascade
axon guidance
neuron projection guidance
positive regulation of chemotaxis
regulation of cell growth
neuron projection extension
in utero embryonic development
positive regulation of epithelial cell migration
ameboidal-type cell migration
DOWN_SL (2218)
UP_SL (2322)
GeneRatio
p.adjust

◀ **Figure EV3. MITF-sl protein is a less potent activator than MITF-WT.**

RT-qPCR analysis and the CUT&RUN peaks in indicated genes from dox-inducible A375P cells overexpressing either MITF-WT or MITF-sl of (**A**) endogenous mRNA MITF and mRNA MITF target genes: (**B**) NRP1, (**C**) CDH2, (**D**) PMEL, (**E**) TRIM63, (**F**) TYRP1, (**G**) MLANA, (**H**) TYR, and (**I**) DCT in the dox-inducible A375P overexpressing cells. The cells were treated with doxycycline for 6, 12, 24, and 36 h to induce MITF expression at the same level before harvesting for RNA isolation. Actin and hAPR was used as housekeeping genes. The fold change in target gene expression was assessed in cells overexpressing either MITF-WT or MITF-sl by comparing to those expressing EV-FLAG-HA followed by normalization to the proportion of MITF proteins retained in the nucleus. Error bars represent SEM of at least three independent experiments. Statistically significant differences (Student's *t* test) are indicated by $^{*}P < 0.05$, $^{**}P < 0.01$, $^{***}P < 0.001$, $^{****}P < 0.0001$, and ns not significant. (**J**) Venn Diagram showing the number of peaks shared and different between MITF-WT and the MITF-sl. (**K**) Peaks different between MITF-WT and MITF-sl mutant proteins shown in a Volcano plot ($P < 0.01$). (**L**) Gene ontology analysis of the 10,636 ($P < 0.01$) peaks that are different between MITF-WT and MITF-sl.

 © The Author(s)

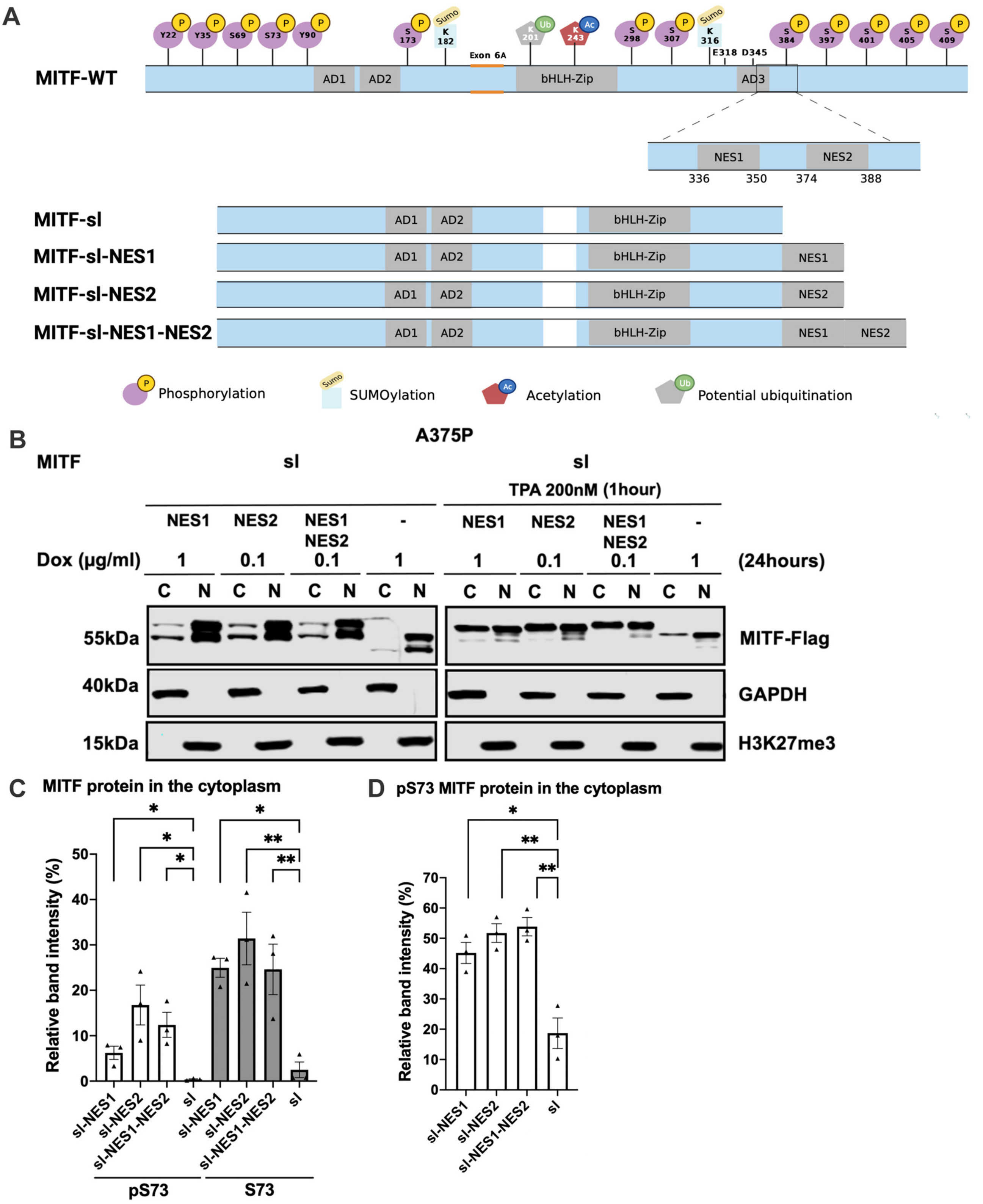
A
MITF-WT
Y22
Y35
S69
S73
Y90
S 173
K 182
Exon 6A
K 201
K 243
S 298
S 307
K 316
E318 D345
S 384
S 397
S 401
S 405
S 409
AD1
AD2
bHLH-Zip
AD3
NES1
NES2
336 350 374 388
MITF-sl
MITF-sl-NES1
MITF-sl-NES2
MITF-sl-NES1-NES2
Phosphorylation
SUMOylation
Acetylation
Potential ubiquitination
B
A375P
MITF
sl
sl
TPA 200nM (1hour)
NES1 NES2 NES1 NES2 -
Dox (µg/ml) 1 0.1 0.1 1
(24hours)
C N
55kDa
40kDa
15kDa
MITF-Flag
GAPDH
H3K27me3
C MITF protein in the cytoplasm
Relative band intensity (%)
sl-NES1
sl-NES2
sl-NES1-NES2
sl
pS73
S73
D pS73 MITF protein in the cytoplasm

Figure EV4. Identify two potential NES at the MITF C-terminus.

(**A**) Graphical depiction of the MITF-WT, MITF-sl, MITF-sl-NES1, MITF-sl-NES2, and MITF-sl-NES1-NES2 proteins. The location of the NES1 and NES2 sequences in MITF-WT are also shown. (**B**) Western blot analysis of cytoplasmic (C) and nuclear (N) fractions from A375P melanoma cells induced for 24 h to overexpress the indicated MITF mutant proteins with or without treatment with 200 nM TPA for 1 h. MITF was visualized using FLAG antibody. GAPDH and H3K27me3 were loading controls for cytoplasmic and nuclear fractions, respectively. (**C**, **D**) MITF band intensities in the cytoplasmic and nuclear fractions from western blot analysis (**B**) were quantified separately with *ImageJ* software and are depicted as percentages of the total amount of protein present in the two fractions. Error bars represent SEM of three independent experiments. Statistically significant differences (Student's *t* test) are indicated by $*P < 0.05$ and $**P < 0.01$.

 © The Author(s)

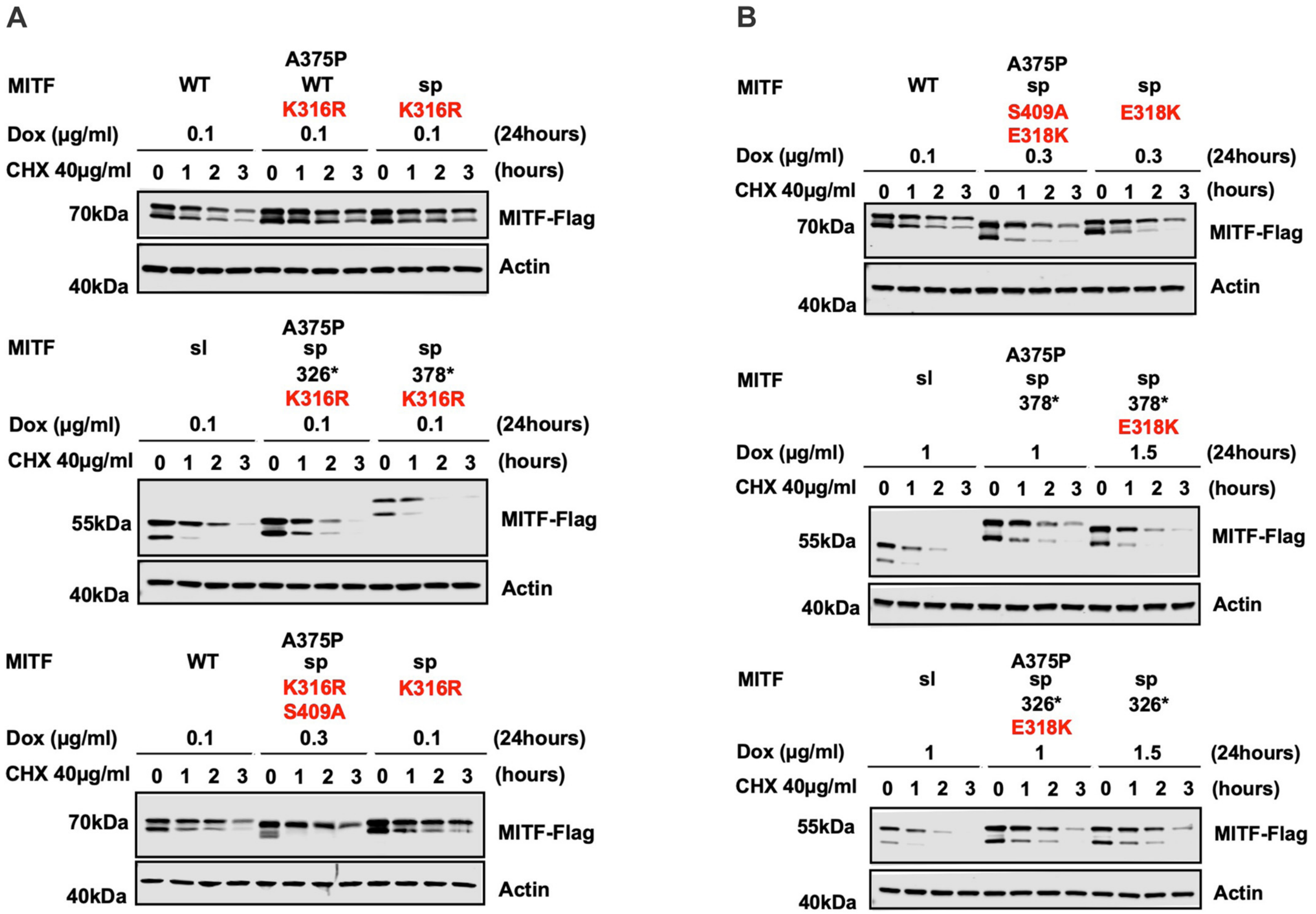

Figure EV5. K316R and E318K mutations effect stability of the MITF proteins.

(**A**, **B**) Western blot analysis of the stability of the MITF proteins. The inducible A375P cells were treated with doxycycline for 24 h to express the indicated mutant MITF proteins before treating them with 40 µg/ml CHX for 0, 1, 2, and 3 h. The MITF protein was then compared by western blot using FLAG antibody. Actin was used as a loading control. The band intensities were quantified using ImageJ software.

© The Author(s)
